# Variable post-release mortality in common shark species captured in Texas shore-based recreational fisheries

**DOI:** 10.1371/journal.pone.0281441

**Published:** 2023-02-13

**Authors:** Addie L. Binstock, Travis M. Richards, R. J. David Wells, J. Marcus Drymon, Kesley Gibson-Banks, Matthew K. Streich, Gregory W. Stunz, Connor F. White, Nicholas M. Whitney, John A. Mohan

**Affiliations:** 1 School of Marine and Environmental Programs, University of New England, Biddeford, Maine, United States of America; 2 Department of Marine Biology, Texas A&M University at Galveston, Galveston, Texas, United States of America; 3 Mississippi-Alabama Sea Grant Consortium, Mississippi State University, Biloxi, Mississippi, United States of America; 4 Harte Research Institute of Gulf of Mexico Studies, Texas A&M University–Corpus Christi, Corpus Christi, Texas, United States of America; 5 Department of Organismic and Evolutionary Biology, Harvard University, Cambridge, Massachusetts, United States of America; 6 Anderson Cabot Center for Ocean Life, New England Aquarium, Boston, Massachusetts, United States of America; Instituto Portugues do Mar e da Atmosfera, PORTUGAL

## Abstract

The practice of catch and release fishing is common among anglers but has been shown to cause unintended mortalities in some species. Current post-release mortality estimates used in coastal shark stock assessments are typically derived from boat-based shark fisheries, which differ from shore-based operations that expose sharks to potentially more stressful environmental and handling conditions. Recreational post-release mortality rates in shore-based fisheries must be quantified to improve stock assessment models and to create guidelines that protect species from overexploitation. Here, we partnered with experienced anglers acting as citizen scientists to deploy pop-up satellite archival transmitting tags (PSAT, n = 22) and acceleration data loggers (ADLs, n = 22). on four commonly caught sharks including the blacktip shark (*Carcharhinus limbatus*, n = 11), bull shark (*Carcharhinus leucas*, n = 14), tiger shark (*Galeocerdo cuvier*, n = 6), and great hammerheads (*Sphyrna mokarran*, n = 2). Mortality occurred within minutes to hours post-release. If evidence of mortality occurred after normal diving behavior had been re-established for 10 days, then the mortality was considered natural and not related to the catch-and-release process. Post-release mortality estimates ranged from 0% for bull and tiger sharks to 45.5% for blacktip sharks. Of the two great hammerheads, one died within 30 minutes post-release while the other exhibited mortality characteristics 14 days after release. Moribund blacktip sharks experienced on average 3.4–4.9°C warmer water compared with survivors. Recovery periods were estimated for survivors of each species and were highly variable, differing based on duration of tag deployment. High variability in responses to capture and release between species demonstrates the need for species-specific assessments of post-release mortality in shore-based recreational fisheries.

## Introduction

The increased popularity of recreational fishing has contributed to the over-exploitation of fish stocks [1–4]. To limit the deleterious effects on shark stocks, catch and release fishing has become a common technique that relies on the assumption that released fish survive and continue to reproduce and fulfill ecological roles [5–7]. However, research has demonstrated that the physiological stress and physical injury of catch and release fishing can cause post-release mortality (PRM) in released fish [4–6, 8–10]. This can happen directly following the event, or as the result of cumulative sub-lethal effects that lower fitness over time, such as tissue damage, blood loss, or metabolic and respiratory acidosis from exhaustive activity [8, 10, 11]. Extensive reviews on conditions contributing to PRM in teleost fish have demonstrated rates ranging from 0–95%, depending on species, fishing techniques, and environmental conditions [4, 5]. Identifying conditions that contribute to unintended fishing mortalities in elasmobranchs is critical to implementing effective guidelines and management strategies, such as gear regulations, species-specific limitations, and seasonal restrictions [12–16].

Post-release mortality rates vary considerably among elasmobranch species [5, 16, 17]. As commonly caught species in the Gulf of Mexico, blacktip shark (*Carcharhinus limbatus)*, bull shark (*Carcharhinus leucas)*, tiger shark (*Galeocerdo cuvier)*, and great hammerheads (*Sphyrna mokarran)* represent four species with variable sensitivity to capture stress [5, 16, 17]. Species-specific PRM rates from a commercial boat-based long-line fishery have been found to vary considerably, from 1.9% for tiger sharks, to 7.1% for bull sharks, and up to 41.9% for blacktip sharks [16]. Great hammerheads have been shown to have a particularly high risk of mortality in commercial long-line fisheries, with at-vessel mortality rates reaching 94% [18]. However, the effects of capture and risk of PRM associated with recreational angling remains understudied in sharks, particularly in shore-based fisheries [18, 19].

Shore-based recreational shark fishing has been expanding where sharks are abundant nearshore, outpacing commercial fishing in some coastal habitats [12, 20, 21]. The Gulf of Mexico attracts thousands of shark anglers yearly in popular shark fishing tournaments, such as the Texas Shark Rodeo which alone has seen over 8,000 sharks caught since 2014 [22]. Analysis of catch records from recreational fishermen has shown a decline in size and abundance of multiple species of large coastal sharks along the Texas coast from 1973 to 2015, suggesting shifts may have occurred in the nearshore assemblages of sharks following a time of increased exploitation [20]. Though recreational fishing introduces negative consequences for shark stocks, it also presents an opportunity to engage anglers in conservation and citizen science. Partnering with experienced shark anglers to deploy tags allows researchers access to critical data on the post-release fate of sharks under typical capture conditions and handling practices within recreational fisheries, which would otherwise be challenging to obtain [22].

This investigation cooperatively engaged recreational shore-based shark anglers to deploy two types of tags, pop-off satellite archival tags (PSATs) and acceleration data loggers (ADLs) on blacktip sharks, bull sharks, tiger sharks, and great hammerheads, four commonly caught species with known variability in capture sensitivity in boat-based fisheries. Partnering with anglers as citizens scientists provides a representative perspective of shark fates under normal fishing conditions in an actual recreational fishery, allowing researchers to make species-specific recommendations on best fishing practices. Electronic tags also provide information on environmental conditions, which can be used to identify conditions that may increase the risk of mortality. The goal of this study was to provide species-specific PRM estimates and identify environmental conditions that contribute to mortality in large coastal sharks in the Texas shore-based recreational fishery.

## Methods

This research was approved by the Texas A&M University–Corpus Christi Institutional Animal Care and Use Committee (IACUC) under protocols #08–15 and #08–18, and by Texas Parks and Wildlife (TPWD) Scientific permit numbers SPR-0303-279. For Texas A&M University–Galveston, research was approved by IACUC #2017–0056 and TWPD scientific permit #SPR-0912-981.

### Sampling location and design

**Fig 1:**
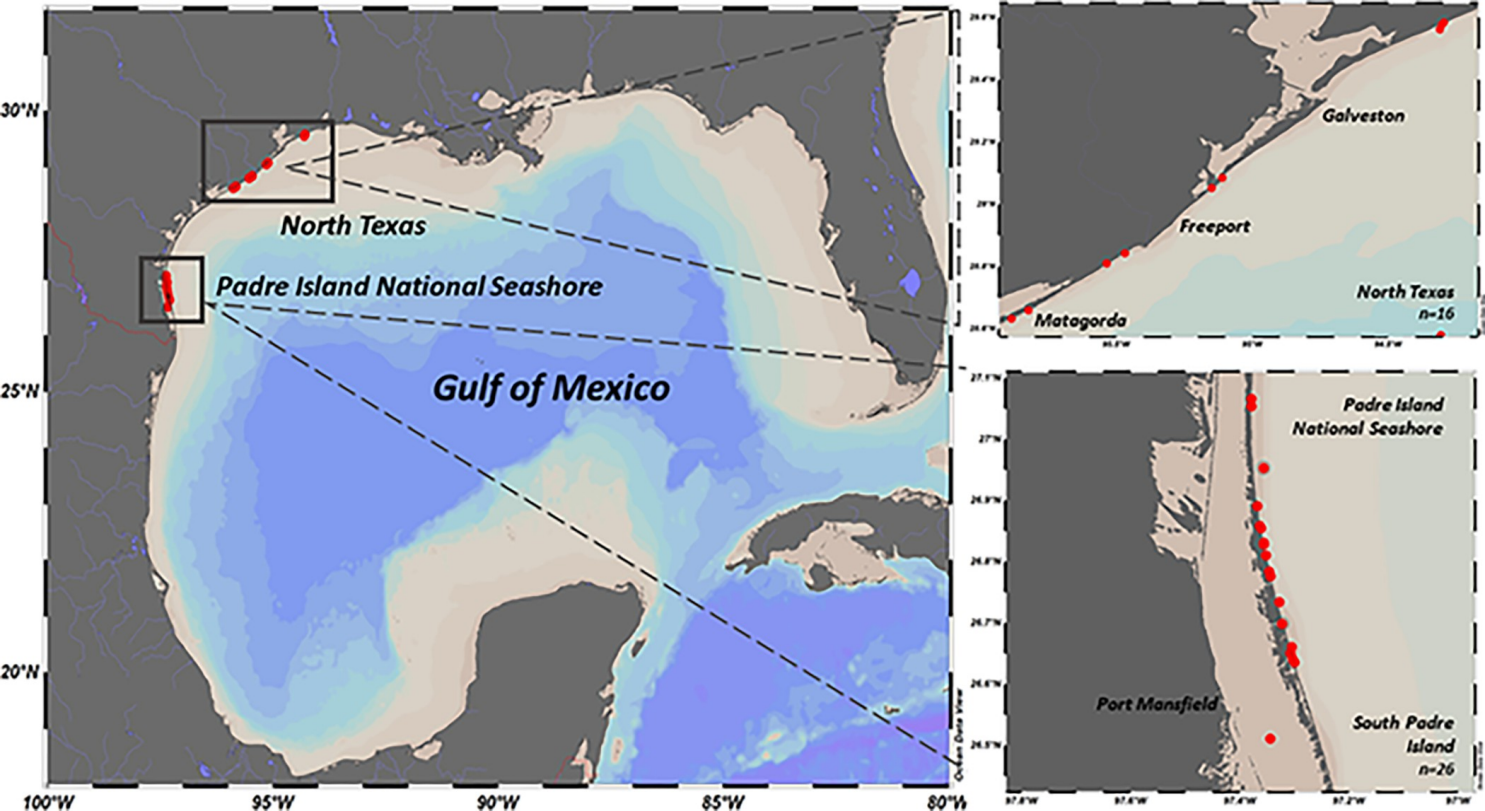
Map of deployment and pop-off locations for PSAT and ADL tagged sharks.

Experienced Texas shore-based anglers from Galveston Bay, Port Aransas, and Padre Island National Seashore (PINS) were recruited to deploy tags on sharks that they captured (Fig 1).

To ensure anglers practiced typical fishing and handling techniques and utilized conventional recreational tackle, no input on fishing or handling practices was provided by researchers other than training in data collection and tag deployment. After typical methods of angling to bring the shark up to the shoreline, anglers removed sharks from the water at their discretion for tagging. Anglers often dragged sharks with a tail rope or by grabbing the tail to beach them, and then recorded lengths, attached tags, and took photographs before release, as is common practice among these shore-based recreational fishermen (Banks et al., [Unpublished])). Fishing and tagging activities were conducted from August 2018 to December 2021. Metrics for data collection included fight time (min), handling time (min), fork length (FL), sex, and condition upon release. Fight time refers to the time from when the shark was hooked to the landing of the shark for measurement and tag deployment. Handling time refers to the time from when the shark is landed on the beach with gills partially or fully exposed to air, to the sharks release back into the water with the fish fully submerged. Release condition was determined using the vitality code from [23] and the indices suggested by [24], which are described as either good, fair, or poor, depending on the tail movement and speed of full submergence in water.

### Tag types and deployment

Four models of pop-off satellite archival tags were utilized, including PSATLIFE (n = 6, 131 mm × 42 mm 87g, Lotek Wireless Inc., Ontario, Canada), sPATs (n = 12, 124m ×38mm, 60g, Wildlife Computers, Redmond, WA, USA), miniPATS (n = 3, 124mm × 38mm, 60g, Wildlife Computers, Redmond, WA, USA), and X-tags (n = 1, 330 mm ×219 mm, 46g, Microwave Telemetry, Columbia, MD, USA).

After landing the shark, a hole was drilled in the leading edge of the first dorsal fin to attach the PSAT using a tether of monofilament or nylon coated stainless steel wire covered in Tygon tubing to reduce chaffing, then crimped with double barrel crimps as close to the fin as possible to reduce drag [11]. All PSAT tags recorded pressure as a proxy for depth, water temperature, and changes in light intensity at high-frequency intervals. Archived tag data were transmitted via satellite in low resolution bins, or downloaded in high-resolution when tags were physically recovered. PSATs were programmed to release after 30 days for PSATLIFE and X- tags, 60 days for sPATs, and 180 days for miniPATs. The PSATLIFE, sPAT and miniPAT tags were pre-programmed with a mortality clause triggering release when certain conditions were detected for 2–5 days. For these tags, the release sequence was initiated when the tag registered a constant depth within a variance of 2 m, or if the tag sank below a crush depth of 1500m [8, 25, 26]. The Microwave Telemetry X-tag did not have a preprogrammed conditional release setting in response to constant or crush depths, and thus released only when the deployment interval was met. Following release, data were transmitted via ARGOS satellites and when recovered, high resolution datasets were downloaded for interpretation. High-resolution datasets from recovered PSATs provided pressure as a proxy for depth, water temperature, and light intensity readings at 10s intervals throughout the entire deployment. Unrecovered PSATs reporting via satellite transmission provided summary data including daily minimum and maximum values for water temperature and depth, and daily changes in light intensity. For unrecovered sPAT tags, high-resolution datasets for only the final five days of deployment were provided via satellite transmission.

The ADL tags in this study recorded triaxial acceleration at a frequency of 25 Hz, and both depth and water temperature at a frequency of 1 Hz (TechnoSmArt, Rome, Italy; model: Axy4-depth). Following typical shore-based fishing practices, captured sharks were tagged by drilling two holes in the first dorsal fin and securing the package with custom plastic cable ties attached to a galvanic timed-release (International Fishing Devices Inc., Northland, New Zealand). The galvanic timed-release dissolves in seawater at various durations from 10 to 72 hours following deployment. The large amount of data collected by ADLs requires that tags be physically recovered. This was done by building custom float packages following the methodology of [27] and [28]. Each ADL was placed in a float package (101×65×45 mm in size, 150 g in air, 18 g positively buoyant in seawater) that also included a VHF transmitter (MM120B; Advanced Telemetry Systems, Isanti, MN, USA) and SPOT satellite transmitter (SPOT 386A; Wildlife Computers, Seattle, WA, USA) for package location and recovery. Tags were located using a hand-held VHF receiver (R410, Advanced Telemetry Systems) or recovered by the public and collaborators by searching for the last transmitted ARGOS position from the SPOT tag, which transmits position estimates via satellite when the tag is exposed to air at the surface.

### Post-release behavior and mortality analysis

High-resolution datasets for water temperature, depth, and light level from each PSAT tag were plotted as a time-series in Igor Pro (Wavemetrics version 9, Portland, OR, USA). When high-resolution data were unavailable, daily summaries of minimum and maximum values for water temperature, depth, and light level were plotted in PRISM (Graphpad, version 9.1.2, 225, San Diego, CA, USA).

Wildlife Computer’s PSAT tags (sPAT, miniPAT, and X-tags) are optimized for survivorship analyses, where pop-off reasons are described by the company and are used to infer instances of mortality. Fate is categorized as either meeting the interval (deployment complete), a “sinker” condition, where the animal sank to the crush depth of the tag (~1500m), a “floater”, where the tag released prematurely and is floating at the surface, or a “sitter”, where a constant depth (~2m variance, 2–5 days) shallower than the crush depth is recorded. Mortality is indicated for either the sinker or sitter condition, and floater condition can be interpreted with visual inspection of depth, light, and water temperature data. Upon visual inspection, mortality events were identified by periods of constant depth indicating a lack of swimming movements, since the four target species are obligate ram-ventilators, requiring constant motion to ventilate the gills for respiration [26, 29], which is reflected by consistent depth changes [30]. Ingestion events were determined by a period of constant depth, indicating a mortality occurred, followed by depth variations, low light levels, and stable temperature, suggesting the tag was inside a predator [11, 28, 31]. For tags hypothesized to have been ingested (n = 2), tag recovery revealed an intact tether (n = 1), indicating the release mechanism was not triggered. This supports the hypothesis that the tagged shark experienced mortality and was preyed upon before the mortality clause could be met to trigger release.

For ADLs, high-resolution datasets for triaxial acceleration, depth, and water temperature were plotted as a time-series in R (version 4.0.3, R Foundation for Statistical Computing) and Igor Pro and examined to determine the shark’s fate. Mortality was indicated when the depth trace was constant on the seafloor and acceleration data revealed a lack of body movement, with time of death estimated as the moment the shark reached the seafloor and stopped moving [30].

### Post-release survivorship behavior and recovery

Survivorship was indicated when the interval for the deployment had been met, indicating no mortality release clause was triggered and the tag was affixed to a moving animal for the entire planned duration of deployment [32]. Sharks were further confirmed to have survived if depth, water temperature, and light level fluctuations were consistent with normal shark behavior and ambient ocean conditions throughout the deployment. When high-resolution datasets were not available, plots were examined for conditions in which the daily minimum and maximum depths were significantly different, suggesting normal diving behavior continued to occur daily.

For sharks that survive capture and tagging, electronic tags can evaluate swimming performance metrics that may be related to behavioral changes following release. Irregular dive behavior consists of inconsistent or restricted depth use [33–35], increased tailbeat frequencies and elevated overall dynamic body acceleration (ODBA) [28], and reduced frequency and amplitude of dives resulting in a diminished dive variance [36, 37]. Quantifying these changes over time allows for a recovery period to be estimated as the amount of time it takes for an individual to restore normal values for these swimming performance metrics.

Using acceleration data from ADL-tagged sharks, tailbeat period (TBP) and overall dynamic body acceleration (ODBA) were derived using the methodology described in [28, 38]. The limited sample size within a species and lack of distinct recovery period within the recording duration prevented the use of nonlinear mixed modeling to quantify recovery period [28]. Therefore, generalized additive mixed models were used to estimate the relationship between swimming metrics (ODBA and TBP) and time post-release. Swimming metrics were summarized as 10-minute means for each surviving individual and individual was used as a random effect in the model to account for the non-independence of measurements. By examining the patterns in swimming metrics with respect to time post release we were able to qualitatively estimate recovery period as the time where swimming metrics appeared to reach an asymptotic plateau.

Dive variance was explored using the depth data for ADL and PSAT-tagged sharks that survived using a break-point analysis [39] as previously described for recovery analysis in pelagic sharks [36, 37]. Unrecovered tags provided low-resolution data and thus were not included. For recovered tags providing high resolution data, variance was calculated over each hour from depth readings recorded every 10s for PSAT tags and every 1s for ADL tags. A marked increase in variance occurred once individuals began normal oscillatory diving behavior where bathymetry permitted an increased range of depth utilization.

Break-point analyses have been used to discern the point of return to normal dive variance for pelagic shark species captured in boat-based fisheries; however, this study represents the first time this methodology has been employed for coastal shore-caught species and may be confounded by the impacts of shallow coastal bathymetry. This methodology was preferred due to variability between the tag types that prevented more sophisticated analyses, however, limited sample size within each species and high variability based on duration of tag deployment prevented species-specific generalizations to be made with confidence. Despite limited depth availability immediately following release for shore-caught species, the break-point analysis method allows relative dive variance to be assessed independently from discrete dive amplitudes, but interpretations should be made with caution.

Sharks that survived and recovered from capture and release were examined for mortality characteristics after the re-establishment of normal diving behaviors to explore instances of natural mortality. If mortality occurred after the individual met its recovery period and re-established normal diving behavior for a minimum of 10 days, it was classified as a natural mortality [37, 40–43]

### Statistical analysis

All statistical analyses were performed in R version 4.0.3 (R Foundation for Statistical Computing). Post-release fate was determined for each shark, and species-specific PRM rates were calculated as the percentage of the total number of recovered datasets that died after release for each species. The error associated with species-specific PRM rates was estimated in R using the Clopper-Pearson exact model to calculate the 95% binomial confidence interval [44]. Due to the small sample size, Fisher’s exact tests were used to compare species-specific mortality rates across each of the four species. Fight time, handling time, release condition, shark length, and sex were recorded at the time of capture as covariates related to capture. To establish covariates related to initial environmental conditions upon capture, average water temperature and average depth over the first 10 minutes immediately following release were calculated for each shark. Limitations in sample size prevented typical parametric analyses of the relationships between capture and environmental covariates and mortality, such as logarithmic regression or Kaplan-Meier survivorship analyses. Normality (Shapiro-Wilk’s test) and homogeny of variance (Bartlett’s test) were evaluated for each set of variables, and all were found to depart from normality. Therefore, non-parametric analyses were used to assess significant differences between surviving and moribund sharks for all capture and environmental covariates (Kruskal-Wallis test). Sufficient data existed to explore differences in environmental and capture covariates between sharks that survived and those that died only for blacktip sharks. To assess temperature distributions across each shark’s time at large, histograms comparing all temperature values for surviving blacktips to all moribund blacktips were created and a Kolmogorov-Smirnov two-sample test was used to evaluate significant differences in the overall temperature distribution between survivors and mortalities.

## Results

### Capture/Environmental covariates and tag deployment

Between August 2018 and December 2021, bull sharks (n = 20), blacktips (n = 15), tiger sharks (n = 6), and great hammerheads (n = 2) were captured by shore-based anglers, tagged, and released between Galveston Bay and Padre Island National Seashore along the Texas coast. All sharks carried only one type of tag, except for one great hammerhead fitted with both a PSAT and an ADL tag. Of the 43 sharks, 29 were female (67.4%), 12 were male (28.6%), and two were unknown (4.8%). For all sharks, average fight time was 15 ± 11 min (range 3–58 min), and handling time was 6 ± 1.73 min (range 3–9 min). Average initial temperature and initial depth over the first 10 minutes after release were 26.08 ± 3.82°C and 1.16 ± 0.83 m (mean ±SD), respectively. Species-specific averages for capture and environmental covariates were calculated for all tagged sharks, regardless of tag type (Table 1).

**Table 1:** Summary of catch information for each species, including total number of individuals, average ± standard deviation for fork length (cm), fight time (min), handling time (min), and water temperature (°C) calculated for blacktip, bull, and tiger sharks. Hammerheads are reported as raw values due to small sample size.

Species	Total	Fork Length (cm)	Fight Time (min)	Handling Time (min)	Average Water Temperature (°C)
Blacktip	15	133 ± 9	10 ± 4	6 ± 1	26.3±4.16
Bull	20	146 ± 26	18 ± 12	5 ± 1	25.5±4.02
Great Hammerhead	2	175, 168	18, 22	8, 7	25.5±2.62
Tiger	6	210 ± 26	33 ± 17	7 ± 2	29.1±0.601

Of the 22 PSAT tags deployed, six tags (28.6%) did not transmit sufficient data, leaving a total of 16 tags (72.7%) that could be used to determine shark status. Of these 16 tags, ten (62.5%) were recovered and six (37.5%) transmitted data via satellite. Both the archived and transmitted data from the 16 PSAT tags were used to determine post-release fate (Table 2).

**Table 2:** Results of 22 PSAT tags deployed on bull sharks, blacktip sharks, tiger sharks, and great hammerheads in the Gulf of Mexico by shore-based recreational anglers. The tags from Shark IDs in bold were recovered, providing high-resolution datasets. FL = fork length; ND = no data. Underlined individuals indicate instances of PRM, and individuals denoted with natural mortality (NM) indicate instances of mortality occurring ≥10 days post release.

Shark ID	Status	Date Capture	Fight (min)	Handle (min)	FL (cm)	condition	sex	days deployed	time to death
C_leu01	ND	3/25/2020	22	-	213	good	F	0	
C_leu02	ND	3/20/2020	19	-	150	good	F	0	
C_leu03	ND	3/25/2020	28	-	185	good	M	0	
**C_leu04**	survive	3/17/2020	11	6	114	good	F	10	
C_leu05	survive	8/17/2018	-	-	-	-	U	28	
**C_leu06**	survive	8/7/2018	-	-	140	-	M	28	
**C_leu07**	survive	7/20/2019	10	4	130	good	F	28	
C_leu08	survive	7/20/2019	10	5	153	good	F	28	
C_leu09	survive	4/5/2021	14	6	183	good	M	47	
C_leu10	ND	4/24/2021	17	6	157	good	M	0	
C_lim01	ND	12/5/2019	10	5	135	good	F	0	
**C_lim02**	mortality	8/15/2018	-	5	144	-	U	16	1.25hr
**C_lim03**	survive	5/12/2019	10	5	145	good	F	28	
C_lim04	ND	12/27/2019	10	5	150	good	F	0	
**C_lim05**	NM	12/22/2021	8	2	138	good	F	31	10 d
G_cuv01	NM	8/20/2020	49	5	231	good	M	57	40 d
G_cuv02	survive	8/22/2020	58	9	287	good	F	58	
**G_cuv03**	survive	6/14/2021	17	7	203	good	M	5	
G_cuv04	survive	7/3/2021	23	8	226	good	F	60	
**G_cuv05**	NM	8/26/2021	23	8	226	good	F	60	41 d
**S_mok01**	mortality	10/21/2020	18	8	175	fair	F	8	30 min
**S_mok02**	NM	4/12/2021	22	7	168	fair	F	16	14 d

Of 22 ADL tags deployed, one tag was lost and three malfunctioned, leaving 18 tags (81.8%) that provided high-resolution datasets of triaxial movement, depth, and temperature upon tag recovery (Table 3).

**Table 3:** Results of 22 ADL tags deployed on bull sharks, blacktip sharks tiger sharks, and great hammerheads in the Gulf of Mexico by shore-based recreational anglers. FL = fork length, ND = no data. Underlined individuals indicate instances of PRM.

Shark ID	Status	Date Capture	Fight (min)	Handle (min)	FL (cm)	condition	sex	hours deployed	time to death
C_leu11	survive	5/13/2020	8	5	129	fair	M	50.2	
C_leu12	survive	7/10/2020	7	7	130	good	F	15.4	
C_leu13	survive	10/17/2020	23	9	147	poor	F	13.9	
C_leu14	survive	10/13/2020	5	5	119	fair	F	10.9	
C_leu15	survive	10/13/2020	10	3	130	good	F	50.8	
C_leu16	ND	10/13/2020	7	4	135	fair	M	na	
C_leu17	survive	10/30/2020	8	4	130	good	M	35.4	
C_leu18	survive	10/30/2020	7	4	130	good	M	37.2	
C_leu19	survive	11/19/2020	11	5	137	good	F	16.1	
C_leu20	ND	11/22/2020	390	6	147	fair	F	na	
C_lim06	survive	7/2/2020	3	3	135	fair	F	10.1	
C_lim07	mortality	9/26/2020	10	6	126	fair	F	22	1 hr
C_lim08	mortality	10/1/2020	17	6	132	good	F	5.53	5 hr
C_lim09	survive	10/30/2020	16	7	130	good	F	69.2	
C_lim10	survive	11/1/2020	11	5	137	good	F	77.1	
C_lim11	ND	11/14/2020	12		135	good	F	na	
C_lim12	ND	5/20/2021	11		127	fair	F	na	
C_lim13	mortality	6/12/2021	6	8	135	good	F	0.62	immediate
C_lim14	mortality	6/16/2021	6	5	119	poor	F	25.8	1hr
C_lim15	survive	6/16/2021	4	8	114	good	F	5.28	
G_cuv06	survive	7/13/2021	25	3	89	good	M	14.3	
S_mok01	mortality	10/21/2020	18	9	175	good	M	23.6	30 min

### Post-release mortality rate estimates

Six of the 20 tags (30%) deployed on bull sharks did not provide sufficient data to determine shark fate. The remaining 14 reporting tags revealed all bull sharks survived the catch and release process. All six tags deployed on tiger sharks provided adequate data to determine recovery and survival in the period following the tagging process. For all species, survival was indicated by sustained depth changes indicating shark movement throughout the deployment and temperature and light conditions that were consistent with ambient ocean conditions. Two tiger sharks exhibited natural mortality over 40 days after tagging, where a re-establishment of normal diving behavior for over 10 days was followed by a period of constant depth that triggered tag release >40 days after the sharks were initially released (G_cuv01, G_cuv05). Of the two great hammerheads, one survived for 14 days post-release before exhibiting a period of constant depth of 12 hours (S_mok02). The tag then released prematurely and floated on the surface for 3 days before being recovered, where it was found that the attachment tether had pulled through the crimp. The other great hammerhead that was tagged with both a PSAT and an ADL (S_mok01) and gradually sank to the bottom over the first 30 minutes after release, experiencing immediate mortality. The deceased hammerhead then rolled around on the seafloor for about 11 hours before the tag was ingested by a predator, as indicated by a period of constant depth followed by a return to depth changes that are consistent with diving behavior in addition to a constant temperature and no change in light, suggesting the tag was inside a predator’s stomach. This tag stayed in the predator’s stomach for ~106 hours before being regurgitated, which was indicated by a depth change in which the tag shot to the surface, where it remained and began registering ambient ocean temperature and light levels.

**Fig 2:**
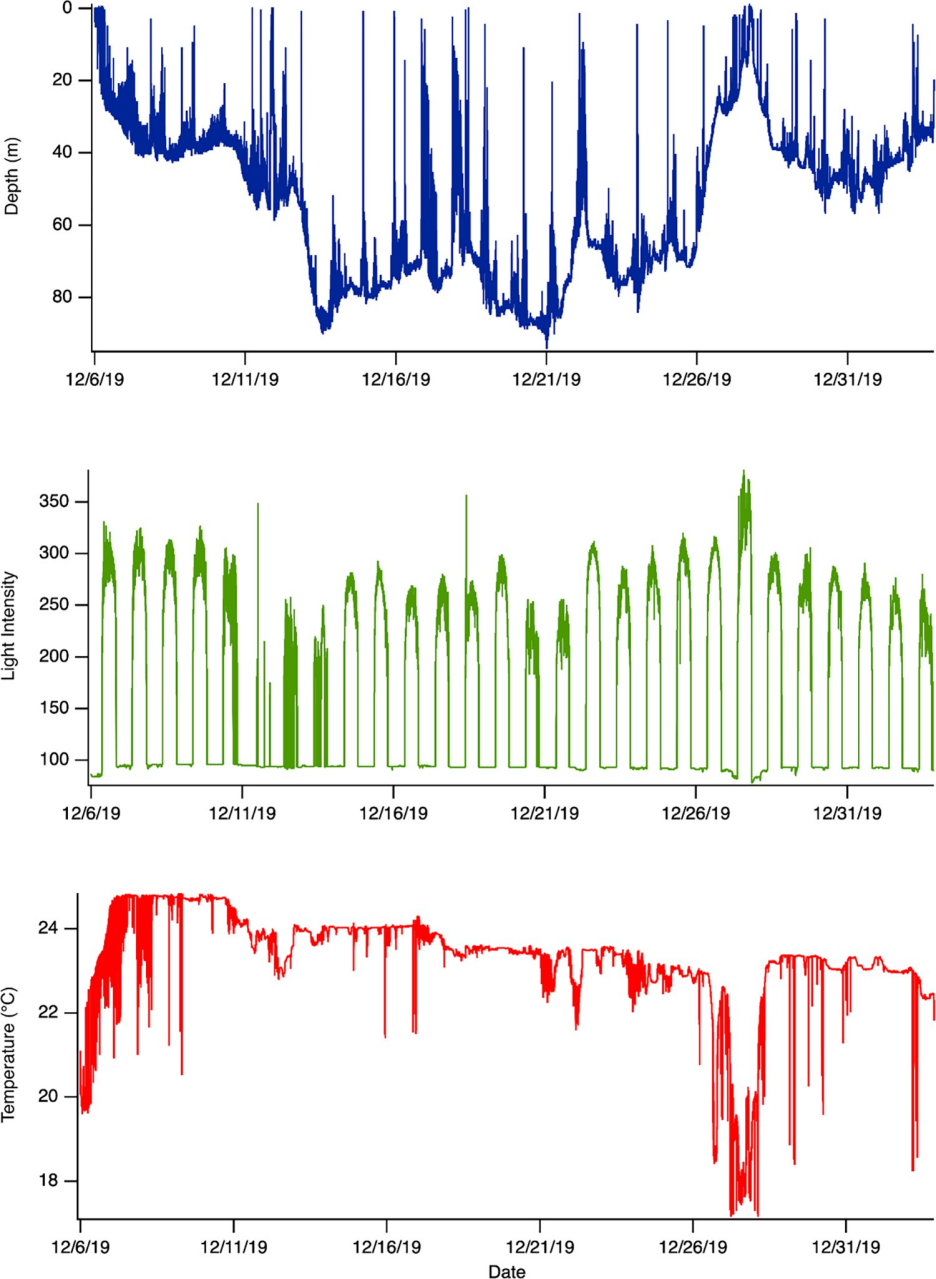
Example of recovered PSAT data from a blacktip shark (C_lim03). Consistent changes in depth, temperature, and light level with ambient ocean conditions indicate survival.

**Fig 3:**
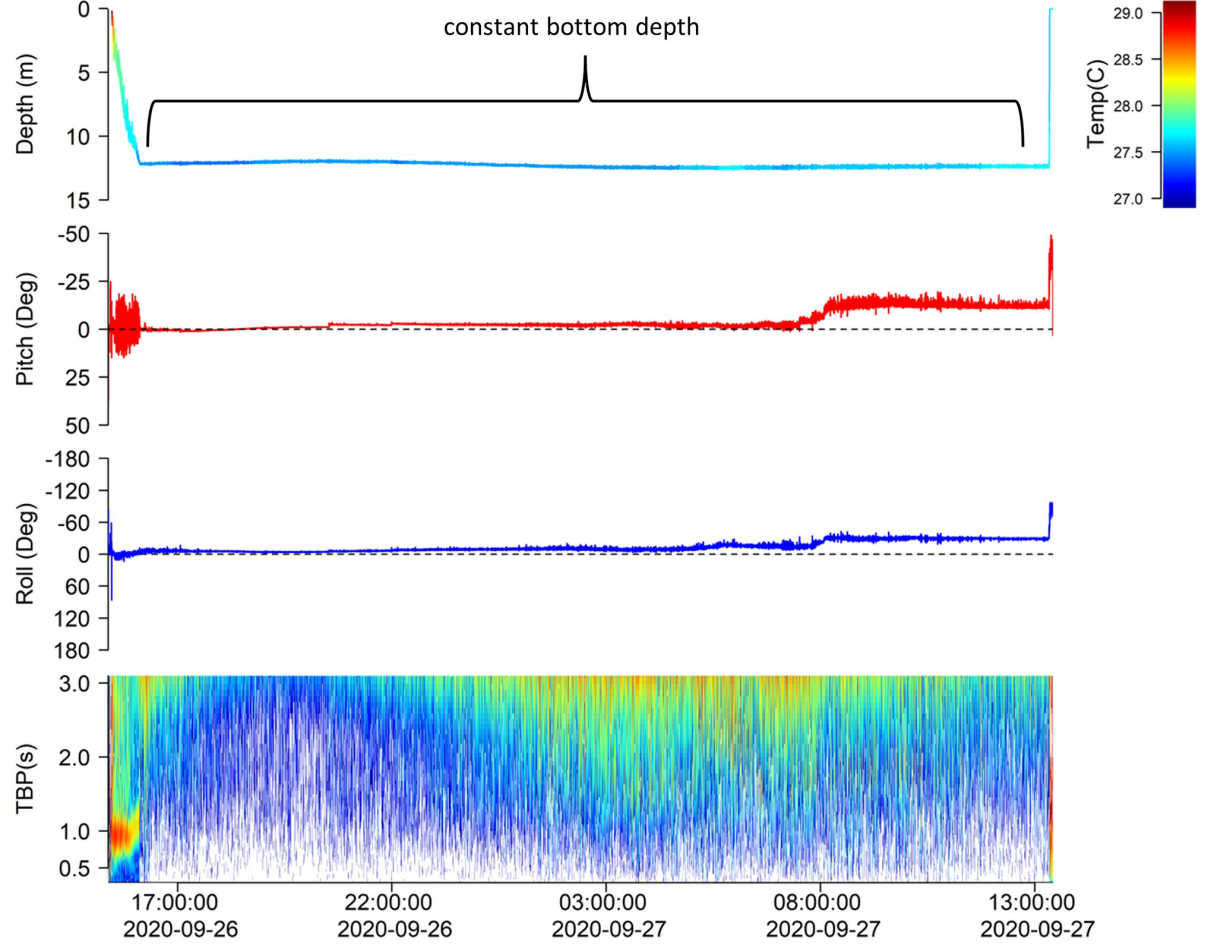
Example of recovered ADL data from a blacktip shark (C_lim07). After tagging, this shark swam away into increasing deeper water, with a tail beat frequency of ~1 Hz. After 40 minutes this shark landed on the seafloor in an upright (normal) posture and stopped displaying regular tailbeats. The shark remained on the seafloor without any tailbeats for a further 21 hours before the release mechanism triggered the tag to detach from the shark. This constant seafloor depth and lack of tailbeats on the seafloor indicates mortality.

Fifteen blacktip sharks were tagged, and 11 tags provided sufficient data for analysis (73%), indicating six blacktips survived (Fig 2) whereas five blacktips experienced mortality within minutes (32min) (Fig 3) to hours, as indicated by a cessation depth changes that would indicate normal shark movement (5h).

**Fig 4:**
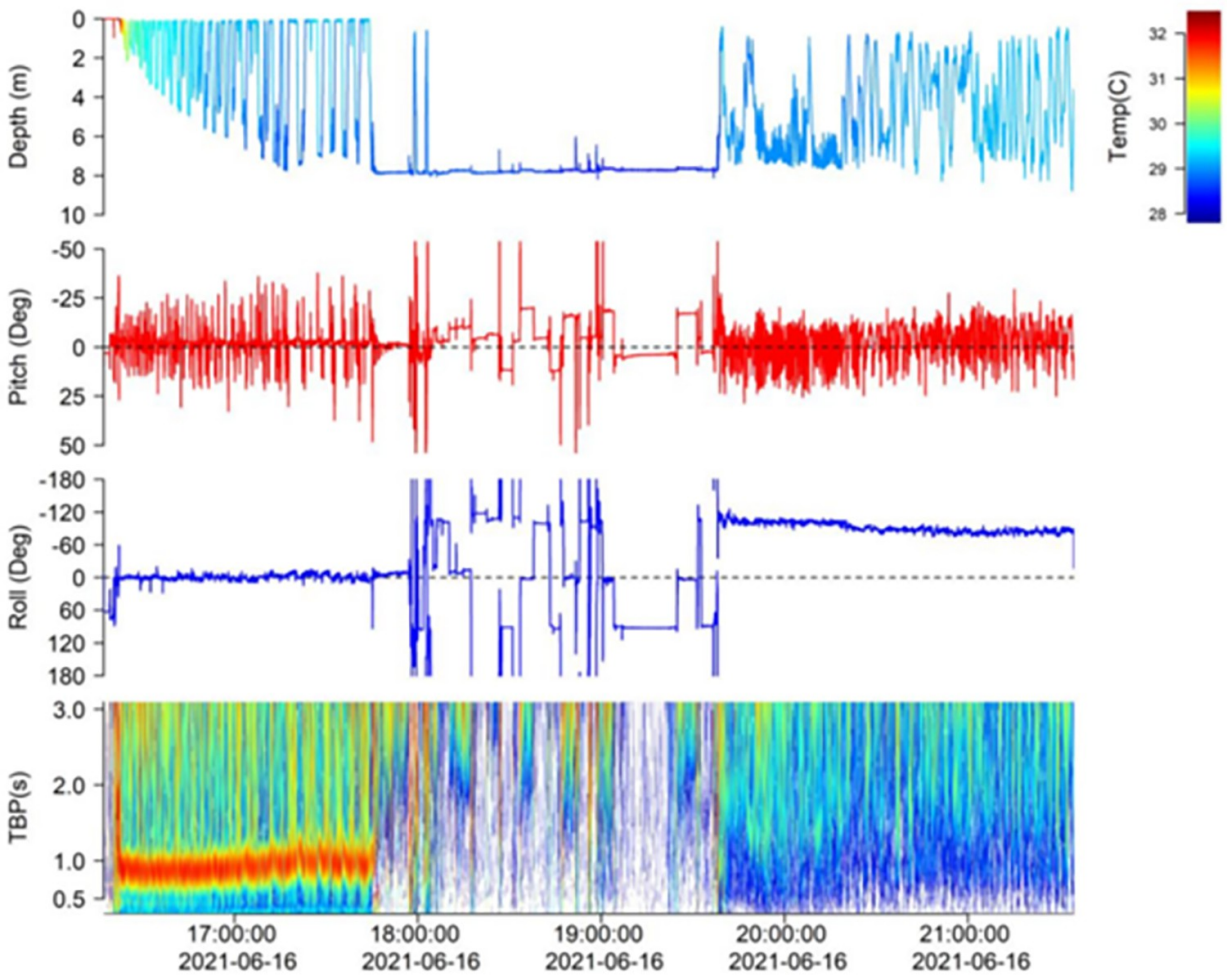
Example of ADL tag data from a blacktip shark (C_lim14). 1 hour after tagging, the shark descended to the seafloor, where it remained for 2 hours at constant depth, indicating mortality. In the first hour post release there are clear tail beats, some temperature stratification and the shark is level (Roll ~ 0). However, this shark then dies as evidence of a lack of tailbeats and laying on the seafloor. After ~10 mins on the seafloor it looks like the shark begins to get scavenged as the shark begins to roll into different positions. 1.5 hours after it initially rested on the seafloor the tag appears to be ingested by a predator. The tag is rolled 120 degrees in the predator’s stomach and because the tag is no longer aligned in the same way, we do not see a clear tailbeat signal in the frequency spectrum. Additionally, as this predator swims up and down in the water column we see no temperature stratification. This tag stayed in the predator’s stomach for ~50 Hours before being regurgitated.

One blacktip shark (C_lim13) was found dead in the surf after multiple attempts to revive and release it, 54 minutes after capture and 32 minutes after the initial release. Ingestion by a predator was indicated for one blacktip (C_lim14) when a period of motionlessness (2 hr) was followed by vertical variations and reduced temperature variability (Fig 4).

**Fig 5:**
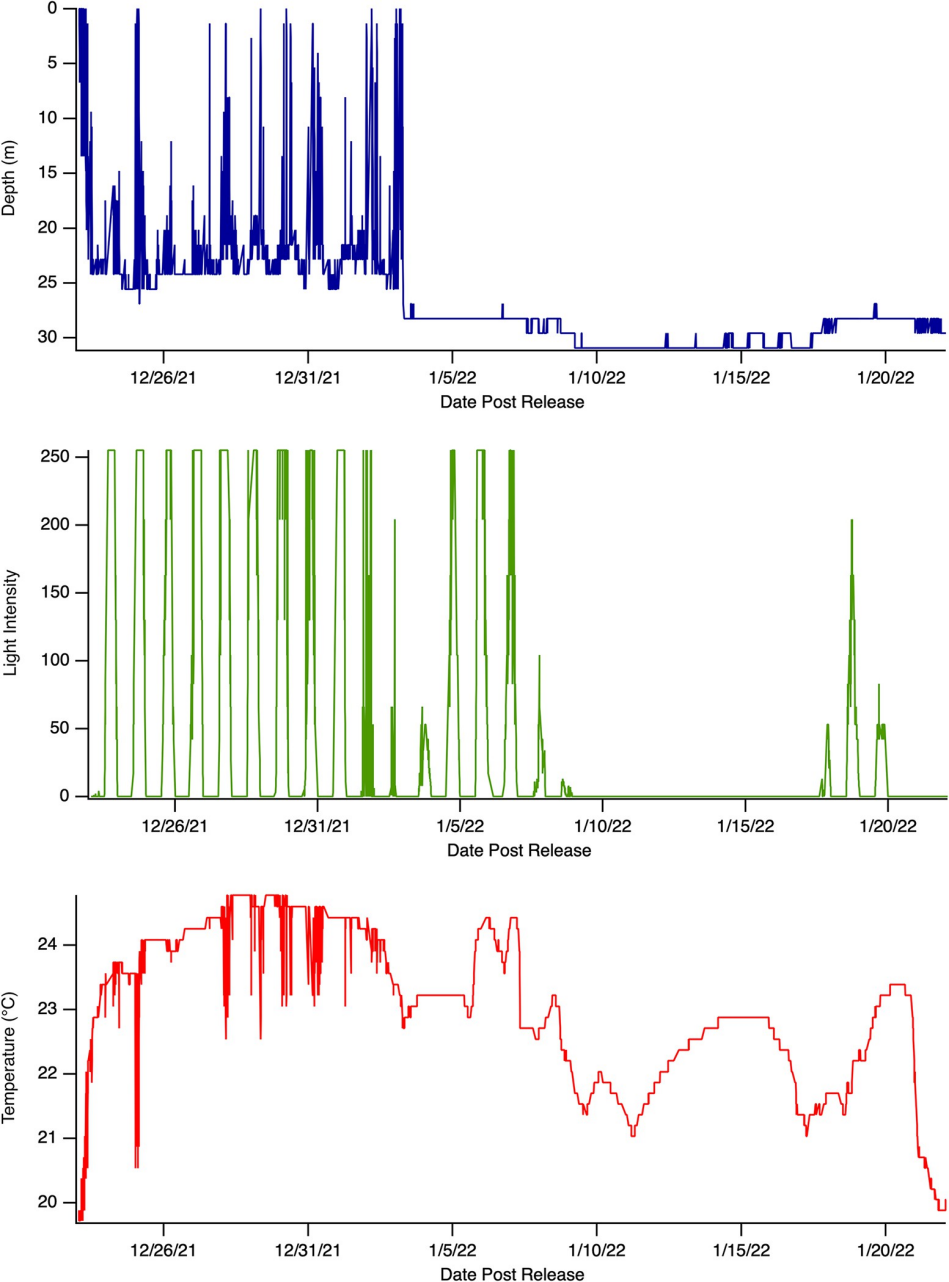
Example of PSAT tag data from a blacktip shark (C_lim05). This individual survived the tagging process and exhibited normal oscillatory diving behavior for 10 days following release. The tag then registers a constant depth at ~30m for five full days, suggesting a natural mortality event occurred, before fluctuating between 28-31m for two weeks and finally releasing at its programmed interval. This tag was not pre-programmed to release when constant depth was detected. This individual was ultimately considered a natural mortality and was not included in the overall PRM rate estimate.

One blacktip shark (C_lim05) re-established normal diving behavior for 10 days after the tagging event, followed by the cessation of depth changes for five days at ~30m, suggesting a mortality event had occurred (Fig 5). This tag was not programmed with a mortality clause to release prematurely in response to a period of constant depth, and therefore met its programmed interval and was recovered.

The overall post-release mortality rate estimates with 95% binomial confidence intervals were 0% (0–45.9%, n = 6) for tiger sharks, 0% (0–2.0%, n = 14) for bull sharks, and 45.5% (16.7–76.6%, n = 11) for blacktip sharks (Table 4).

**Table 4:** Number of mortalities, survivors, and total number of tags that provided sufficient data to determine fate for each species. Post-release mortality rate estimates are reported for blacktip sharks, bull sharks, and tiger sharks, but not for great hammerheads due to low sample size. 95%.

Species	Mortality	Survive	Total	PRM rate estimate	95% CI
Blacktip	5	6	11	45.5%	0.16 to 0.76
Bull	0	14	14	0%	0.00 to 0.02
Great Hammerhead	1	1	2	-	0.01 to 0.99
Tiger	0	6	6	0%	0.00 to 0.46

confidence intervals were calculated using the Clopper-Pearson exact method.

Our small sample size (n = 2, mortality = 1) for great hammerheads produced a confidence interval of 1.3–98.9% and thus was not used to estimate a rate of PRM. Fisher’s exact tests confirmed significant interspecific differences in PRM rates between blacktip sharks and bull sharks (p-value < 0.01) but not between blacktips and tiger sharks or between tiger and bull sharks, likely due to small sample sizes (all p-values > 0.05).

Non-parametric examination of environmental and capture covariates between groups (Kruskal-Wallis’ test) indicated only water temperature was significantly different between blacktip sharks that survived and those that died (p-value = 0.04). No other variable was found to differ significantly between groups (all p-values > 0.05) (Table 5).

**Table 5:** Results of Kruskal-Wallis’ test for significant differences in environmental and capture covariates between blacktip sharks that survived capture and release and blacktip sharks that experienced post-release mortality. Capture covariates included fight time, handling time, and shark fork length. Environmental covariates of initial temperature and initial depth were taken from an individual’s tag data as an average of the first 10 minutes immediately following release.

Covariate	χ2	*p* value	df
Temperature (°C)	4.03	0.045	1
Depth (m)	0.53	0.465	1
Fight Time (min)	0.13	0.712	1
Handling Time (min)	1.7	0.192	1
Fork Length (cm)	0.41	0.522	1

**Fig 6:**
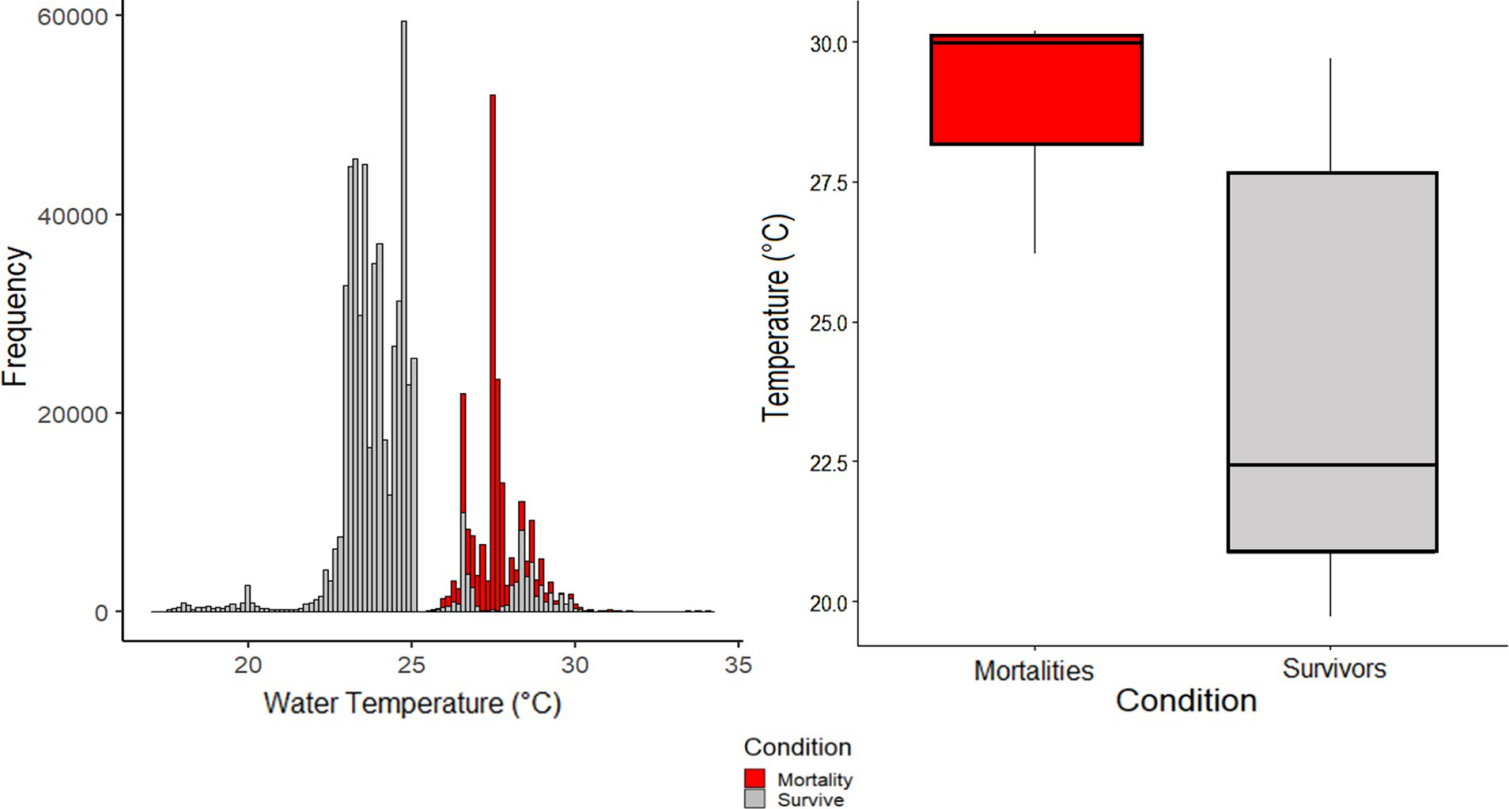
Temperature distributions for blacktip shark survivors (grey) and mortalities (red) across each groups time at large (Left, Kolmogorov-Smirnov two sample test, p<0.0001). (B) Boxplots comparing average initial temperature from the first 10 minutes immediately following release between blacktip sharks that survived capture and release and those that did not (Right, Kruskal-Wallis test, p = 0.045).

The average initial temperature (mean ±SD) was 24.01 ± 4.36°C for survivors, while those that died encountered 28.94 ± 1.72°C water initially, a difference of 4.93°C (Fig 5). Comparison of temperature distributions across the entire deployment were also significantly different (Kolmogorov-Smirnov test, p-value < 0.01) with moribund sharks showing an average temperature 3.35°C higher than those that survived (Fig 6).

### Post-release survivorship behavior and recovery period

ADL derived swimming metrics were calculated for surviving bull sharks (n = 8), blacktip sharks (n = 3), and tiger sharks (n = 1). Great hammerheads were unable to be assessed as no ADL tagged sharks survived. Generalized additive models showed that there were significant changes in swimming metrics over the hours post release. Blacktip sharks displayed increasing TBP which appeared to stabilize approximately 36 hours after release and a decrease in ODBA over the initial 20 hours post release before stabilizing. Trends in bull shark behavior post release were more variable without clear patterns of a recovery period; TBP increased while ODBA decreased over the initial 12–16 hour period after release. After this period, there was a decrease in the number of available datasets and an increase in the displayed variability in behavior. The only tiger shark tagged with an ADL showed an increase in TBP and a decrease in ODBA over the first 12 hours post release (S1 File).

Break-point analysis for all recovered tags on surviving sharks allowed recovery period to be estimated for bull sharks (n = 11), blacktip sharks (n = 6), tiger sharks (n = 2), and one great hammerhead. For bull sharks, dive variance increased after an average of 8 hours ± 7.93 (range 1h-23h). Blacktip sharks had an average recovery of 29.6h ± 35.4 (range 1h-74h). The tiger sharks were seen to exhibit recovery behavior after an average of 14.5h ± 7.8 (range 9h-20h), and low sample size prevented statistical analysis between tag types. The hammerhead increased dive variance significantly after 9 hours (S1 File).

Four instances occurred in which sharks that survived and recovered from capture and tagging were later seen to have exhibit mortality characteristics while still tagged. The great hammerhead S_mok02 was tagged with an sPAT on 4/13/2021 and was seen to re-establish normal diving behavior after 9 hours, which was sustained for 13 days until 4/26/2021. The tag on S_mok02 then shows settles at ~9m for >12 hours, then fluctuates between 8-9m for the following 24 hours before releasing to the surface prematurely. The tag then floated at the surface for 3 days, where it was recovered with the tether pulled through the crimp.

sPAT tagged tiger shark G_cuv01 transmitted low-resolution daily data revealed normal changes in depth, temperature, and light levels from initial release on 8/21/2020 until 9/29/2020, indicating over one month of normal diving behavior. The final five days of deployment are transmitted as high-resolution data, where the tag can be seen to settle at ~73m on 9/29/2020, where it remains until the tag released prematurely at depth on 10/1/2020 with the pin intact.

The tiger shark G_cuv05 was tagged with a mini-PAT on 8/26/2021 and exhibited characteristics of recovery within 20 hours of release. On 10/22/2021, the tag registers a depth of ~10.5m, where it remains for 3 days until 10/25/2021, meeting the mortality clause for premature release. The tag can then be seen rising to the surface, where it was recovered with the pin intact.

C_lim05 was tagged on 12/22/2021 with a microwave X-tag and exhibited recovery characteristics within 8 hours of release. The tag reveals normal diving behavior for ~11 days before settling at ~28m on 1/3/2022, where it remains fluctuating between 27-31m until shutting off on 1/22/2022. This tag was not pre-programmed with a mortality clause to release with the detection of constant depth over multiple days.

Given the normal diving behavior for >10 days in all instances, recovery from the tagging event is indicated, suggesting these sharks all survived the direct physical damage and physiological stress of capture and release. If the subsequent lack of depth variation in these cases was indicative of true mortality events, the cause of death could be related to sublethal mechanisms related to capture stress but cannot be confirmed to be a direct result of fishing pressure. These events were therefore classified as natural mortality events as opposed to post-release mortality events and were not included in overall PRM estimates. If any natural mortalities occurred for ADL tagged sharks, the programmed deployment of 24–48 hours was not long enough to capture delayed indicators of mortality.

## Discussion

The practice of catch and release in recreational shark fisheries is intended to promote conservation; however, information on PRM and individual responses to capture stress in shore-based fisheries is needed to refine stock assessment models and promote sustainable fisheries. Substantial variation among species responses to capture is demonstrated here and supported by a large body of previous work [8, 16, 23, 29, 30, 44–48].

Risk of mortality can also be exacerbated when certain environmental conditions prevent an efficient and effective recovery from the stress of capture, and here we support a large body of work pointing towards increased temperatures as a significant factor reducing survival [16, 23, 49–54]. Many elasmobranch studies have demonstrated that the physiological stress and physical damage from capture and restraint may elicit biochemical responses that affect both short- and long-term fitness following release[4, 8–10, 55–57].

### Post-release mortality rate estimates

Tiger sharks and bull sharks caught and released by shore-based recreational anglers in this investigation had an overall PRM rate of 0%. Although no published studies explore PRM for these species in shore-based fisheries, [16] found a low PRM rate of 2% (n = 51) for tiger sharks in a long-line fishery, which was consistent with the trends seen here in that tiger sharks were among the least sensitive of the multiple species studied. [58] found similar trends for tiger sharks in a longline fishery in the South Atlantic; Their study demonstrated a PRM rate of 0% for tiger sharks (n = 19), but also found natural mortality occurred for one individual after 45 days at liberty. For the six tiger sharks tagged in our study, no instances of PRM were identified, but two individuals were believed to have died following the reestablishment of normal diving behavior for over a month. These trends may suggest that tiger sharks are very resilient to the initial physical damage of capture, but aggregative sub-lethal impacts of physiological stress may compound over time to result in mortality and should be further explored.

In contrast, previous studies on bull sharks have indicated higher PRM rates than seen here. [16] found a PRM rate of 7.1% (n = 14) for longline caught bull sharks, which had significantly longer hook times (average 245 min) compared to our study (average 34 min). Increased hook times may be a mechanism behind the different PRM rates for bull sharks between these studies, indicating further research should explore the effects of hook time on post-release survival in bull sharks.

Great hammerheads were the least tolerant to capture stress in this study, with one hammerhead experiencing immediate mortality, and the other exhibiting mortality characteristics 14 days after release. Despite low sample size for great hammerheads, our results are consistent with other studies in boat-based fisheries that demonstrate the species’ sensitivity to capture stress and high likelihood of PRM [17, 18, 59]. That said, [60] found great hammerheads in a Florida fishery to have a substantially lower PRM rate estimate than most other *Sphyrna* studies at 7.7% PRM [60]. This discrepancy can be explained by inherent differences between the Texas and Florida fisheries; Florida law requires anglers to keep all hammerheads submerged to prevent beaching, whereas Texas anglers often remove sharks from the water, prolonging air exposure (Banks et al., [Unpublished data])). Further investigation is needed to determine the extent to which limiting gill ventilation during handling contributes to mortality.

Blacktip sharks also displayed low resilience to capture stress here, with a PRM rate of 45.5%. Blacktip sharks are one of the most sought-after species of shark by recreational anglers due to their strong fighting characteristics [1]. Despite their high catch rate, blacktip sharks experience a high degree of physiological disturbance in response to the stress of capture [16, 19, 46, 47, 54]. Here, shore-caught blacktip sharks had the highest PRM rate (45.5%) compared with other more resilient species (tiger and bull sharks PRM = 0%). This finding is in accordance with other studies in boat-based fisheries that consistently rank blacktips as one of the more sensitive species to capture stress due to high physiological disturbances [11, 16, 19, 54]

Interestingly, the estimated blacktip PRM rate in this study (45.5%) is substantially higher than most other studies, apart from [16], that explore the fate of blacktips after catch and release. The rate demonstrated here is more than double that reported in [19] for blacktips caught in a shore-based fishery (17.1%). This discrepancy may be attributed to the significantly extended fight and handling times experienced by our blacktips compared to those in [19], which could be due to differences in gear, handling techniques, and the time required to attach different tag types once a shark has been landed. Though statistical analysis did not reveal a significant correlation to handling time in our study, extended air exposure and physical trauma resulting from landing, photographs, and hook removal can damage gill lamellae, further impeding respiration and recovery [52, 61]. As our fight and handling times were 10 and 6 minutes, respectively, compared to [19] with 5.09 and 3.33 minutes respectively, almost doubling the time fish spent fighting and exposed to air may have been a primary factor contributing to our increased PRM rates.

Studies that use rod-and-reel boat-based angling, which is a similar method to shore-based angling, have estimated PRM rates ranging from 9.7% when sharks were handled in the water [54] and up to 22.7% when sharks were handled on the deck of a boat [11]. The difference in handling technique between the two studies highlights the impact of increased air exposure on the ability of blacktips to recover from the stress of handling, which is a key characteristic of shore-based fishing in which anglers often beach sharks. Analysis of angler techniques at the Texas Shark Rodeo revealed only 3.3% of sharks landed were allowed to remain with their gills underwater and aerated during the catch-photo-release process, indicating removal from the water is common practice among Texas anglers (Banks et al., [Unpublished]) To mitigate the effects of air exposure that increase the risk of mortality, fisheries managers can consider suggesting landed sharks should remain with their gills aerated during the handling process.

The other major discrepancy between boat-based and shore-based angling is the increased temperatures sharks experience when caught and landed in the shallow, warm surf. Consistent with other studies that explore the effects of temperature on stress and mortality [16, 23, 49–54], our study suggests that increased temperatures significantly impact the ability of some species to recover and survive following capture and release. Statistical comparison of environmental and capture covariates between blacktip sharks that survived and those that died revealed moribund blacktip sharks experienced significantly higher temperatures both in the initial 10 minutes following release and for the entire time at liberty until mortality occurred compared with those that did survive. Though our sample size in either group was small, this finding is consistent with our understanding of shark stress physiology [16, 23, 46, 49, 50].

Warmer water temperatures have been demonstrated to exacerbate the sublethal physiological disturbances experienced by elasmobranchs [16, 23, 53], resulting in hypoxia, reduced myocardial function, and acidosis [62, 63] In two separate studies, [16, 54] found that higher water temperatures intensified the stress response in blacktip sharks, resulting in substantially higher rates of PRM. For species that tend to fight harder when hooked, the increased oxygen demand in the face of hypoxic conditions due to higher temperatures leads to increased stress and risk of mortality [64]. [52] demonstrated that the interactive effects of both warmer temperatures and longer air exposure strengthens the negative relationship with ventilation and contributes to higher rates of short-term mortality in teleosts. Fisheries managers should consider how these conditions compound to inhibit recovery in the shore-based fishery when developing guidelines to mitigate PRM and the need to consider boat-based operations separately as there are important differences between the two techniques. When the water is warm, seasonal restrictions could promote the overall survival rate for species that are more sensitive to the physical damage and physiological stress associated with being caught and beached in a shore-based fishery.

### Post-release survivorship behavior and natural mortalities

Sharks that are subjected to shore-based capture and release undergo exhaustive exercise while fighting on the line that ultimately results in hypoxia, a condition causing physiological stress that is exacerbated by increased temperatures [8, 16, 52, 57, 65] During this period of physiological stress, individuals have a diminished capacity to engage in their normal activities, which can be visualized as departures from normal swimming performance metrics following release [10] These normal diving behaviors are crucial to negatively buoyant fish to engage in predation while optimizing efficiency and minimizing energy expenditure through movement [34, 66–70]. Therefore, a reduced ability to optimize swimming performance could reduce a fish’s overall fitness by affecting cost-efficient movement, foraging strategies, and predator avoidance [45, 71].

Across all metrics and tag types, blacktip sharks had the longest average recovery period, followed by tiger sharks, with hammerheads and bull sharks exhibiting the shortest recovery period. This is consistent with previous work indicating blacktips have the highest physiological disturbance in response to capture stress of these three species [16, 21]. Overall, our findings are consistent with the generalization that physiological stress is resolved in the first one to three days following capture if an individual survives the event [8, 9, 54, 65, 72].

High intraspecific variability in time to recovery was concluded for all species. This high spread of recovery both within and between tags could be due to inconsistent duration of tag recording, where some individuals were at liberty for 11 hours while others of the same species with the same tag type were at liberty for up to 78 hours. ADL tags are generally deployed for shorter periods of time compared to PSAT tags; however, premature shedding occurred for both tag types leading to some instances of shortened recording periods. The inconsistent recording periods could be a mechanism behind this high variability; therefore, future studies should standardize sampling frequencies and lengths to elucidate physiological trends related to recovery.

Released individuals who are resilient to capture stress are still at risk of mortality from natural causes, including disease and predation, in addition to the aggregative sub-lethal effects of tagging that may reduce overall fitness [8, 57]. When mortality is delayed weeks to months following release, it is typically considered “natural mortality” as it cannot be directly attributed to the stress of capture, as PRM is thought to occur within the hours or immediate days following release [54, 73]. If the natural mortalities seen in this study exceed the general natural mortality rate for a species, it may indicate extenuating circumstances exist that have increased the risk of mortality due to the compounding sub-lethal impacts inherent to capture stress. Further investigations into the sub-lethal impacts of catch and release fishing may seek to extend the window in which a mortality would be considered attributable to fishing to improve the accuracy of PRM rates.

## Conclusions

This study represents one of the first comprehensive analyses combining different tag styles to assess and compare post-release mortality in multiple shark species caught and released in a shore-based recreational fishery. Our findings reveal that capture stress contributes to PRM in certain species, while other species can be sustainably caught and released without substantially increasing the risk of mortality. For species with high sensitivity to stress and low reproductive output, recreational shore-based shark fishing can have negative consequences on overall stocks. The types of data collected during this study allow for the development of species-specific PRM estimates, which can then be used to fine tune stock assessment models and improve protections for sensitive species. Further studies can improve upon this by increasing sample size, allowing for stronger comparison between species-specific responses to shore-based capture under different environmental and capture covariates. For more sensitive species that are known to put up a strong fight, we found temperature to be a contributing factor to the likelihood of survival. For surviving individuals, estimating recovery can be highly influenced by tag duration, highlighting the need for temporal standardization and increased sample sizes to improve accuracy when assessing recovery. The trends revealed here can be used to improve guidelines for shore-based fishermen and provide accurate information for stock assessment models to ensure unintended fishing mortalities do not contribute to the overfishing of sensitive species.

## Supporting information

S1 File Recovery period. Includes all relevant figures with respect to recovery period analysis, including species specific tailbeat frequency and overall dynamic body acceleration calculated from ADL tags, hourly dive variance for all sharks that survived capture and release.
(PDF)

S2 File PSAT and ADL time series. Includes all time series for all datasets obtained from tagged sharks.
(PDF)
