# Supplementary material for: Variable post-release mortality in common shark species captured in Texas shore-based recreational fisheries
Source: PLoS One. 2023 Feb 13;18(2):e0281441. doi: 10.1371/journal.pone.0281441 (PMC9925081; doi:10.1371/journal.pone.0281441)

Supplemental Information II

Recovery Period

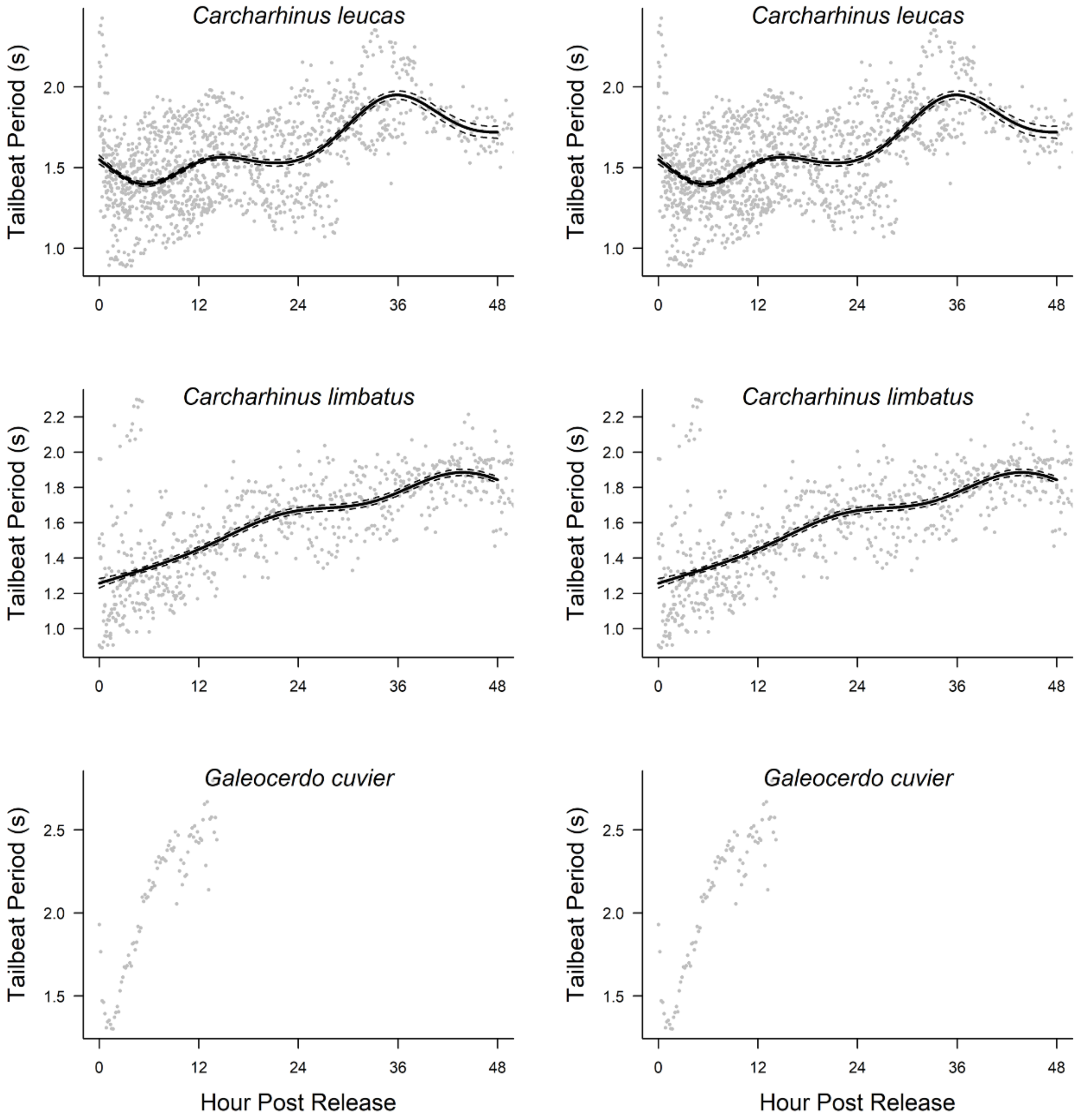

Tail beat frequency (left) and overall dynamic body acceleration (right) calculated from the sway axis of the accelerometer data. Generalized additive models were used to estimate the relationship between ODBA and TBP and time post release over 10-minute means for each surviving individual.

ADL Hourly Dive Variance

C_leu12: 2 hr

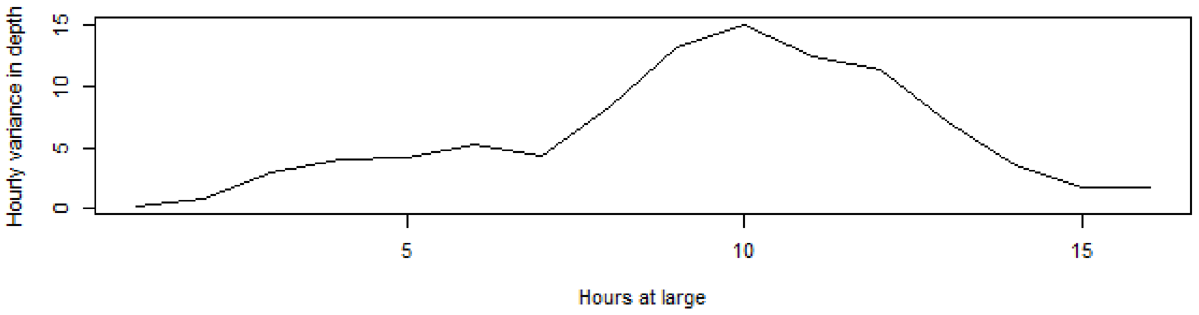

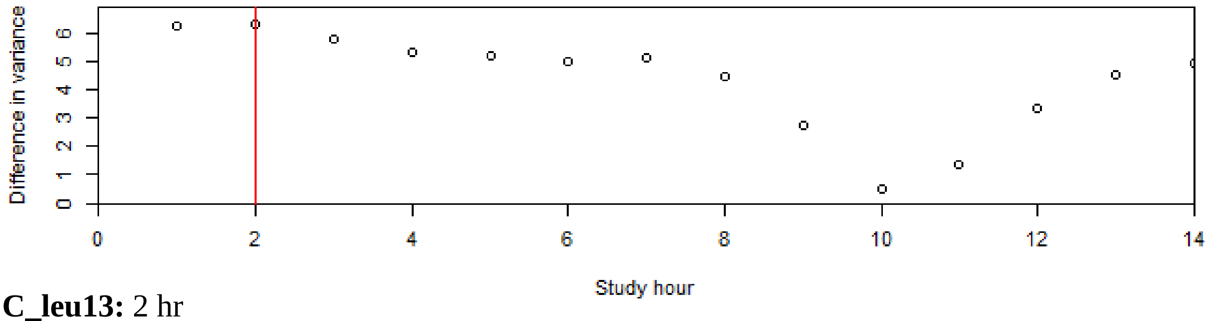

C_leu13: 2 hr

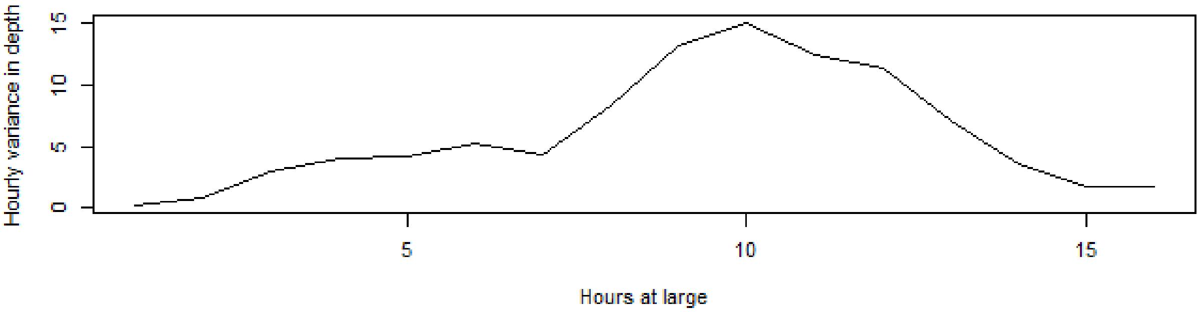

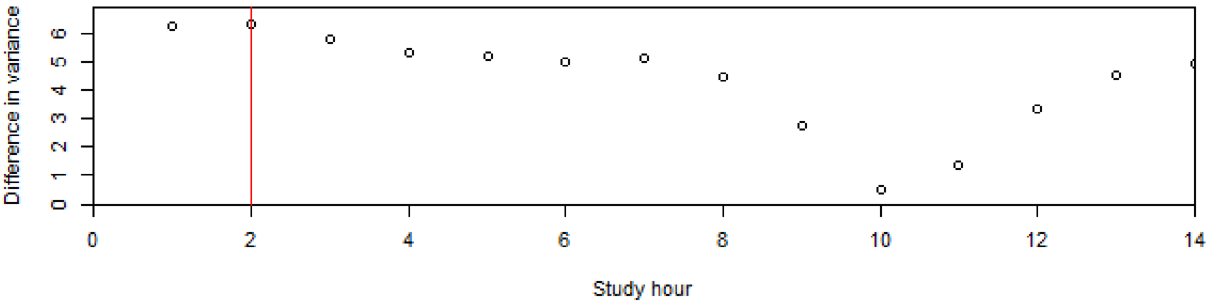

C_leu14: 1 hr

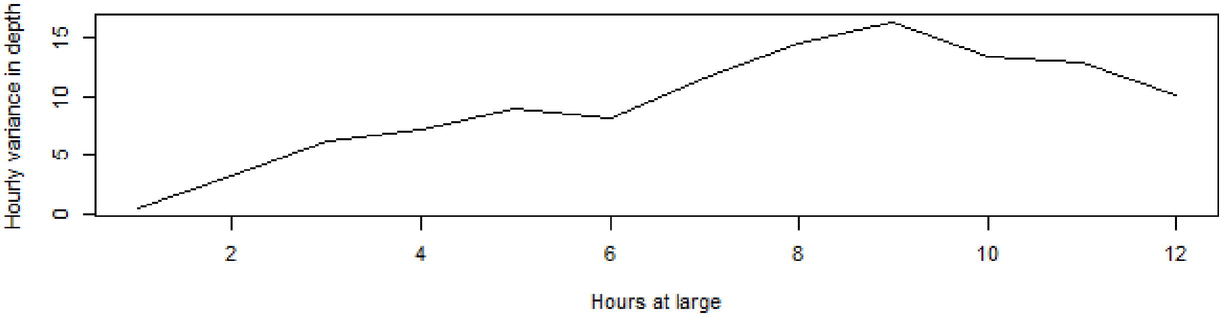

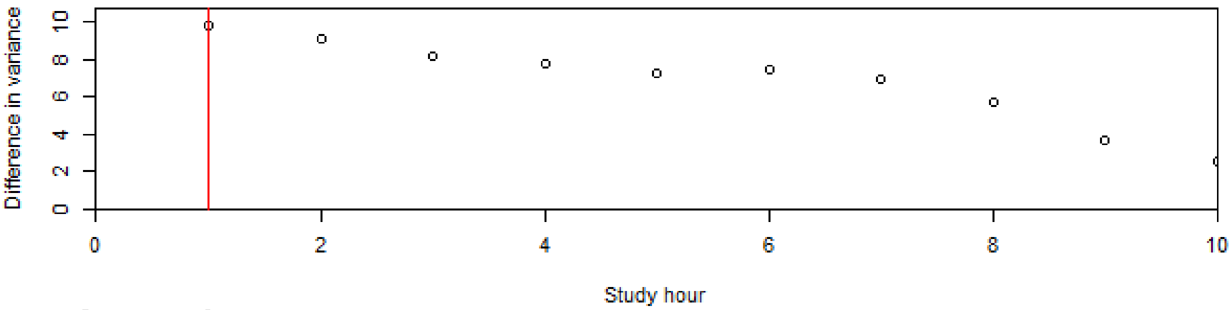

C_leu15: 12hr

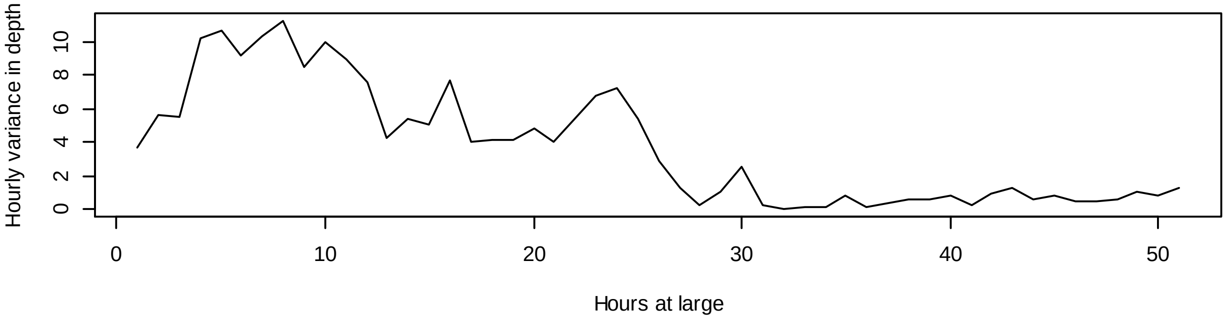

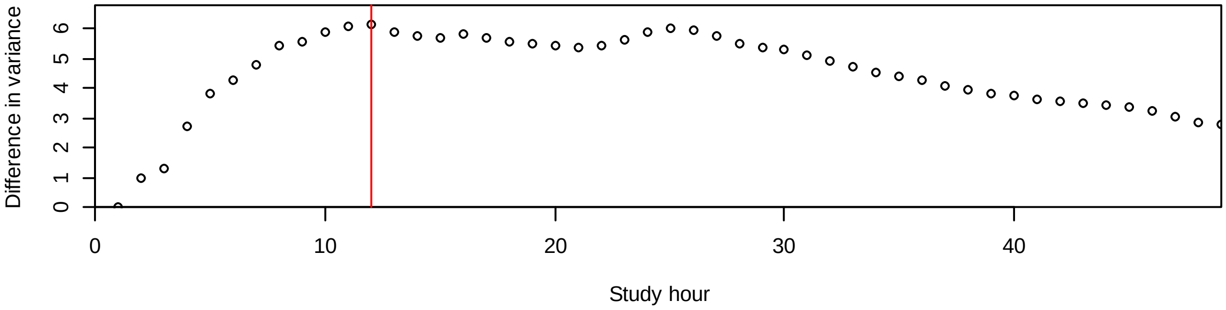

C_leu17: 21 hr

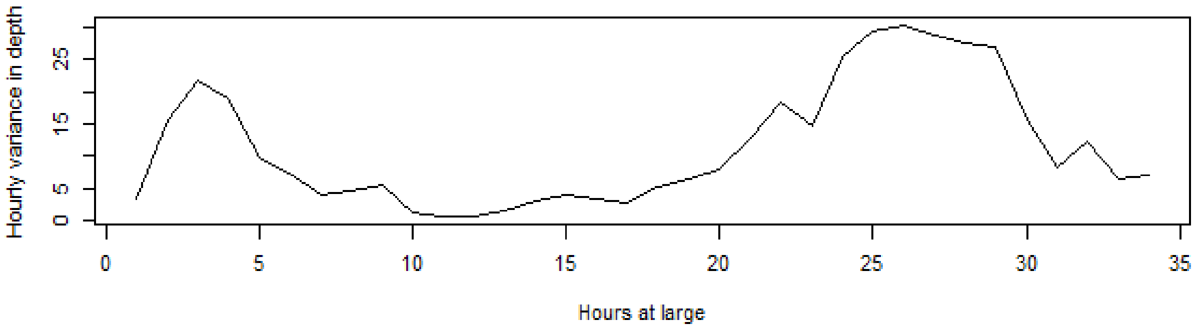

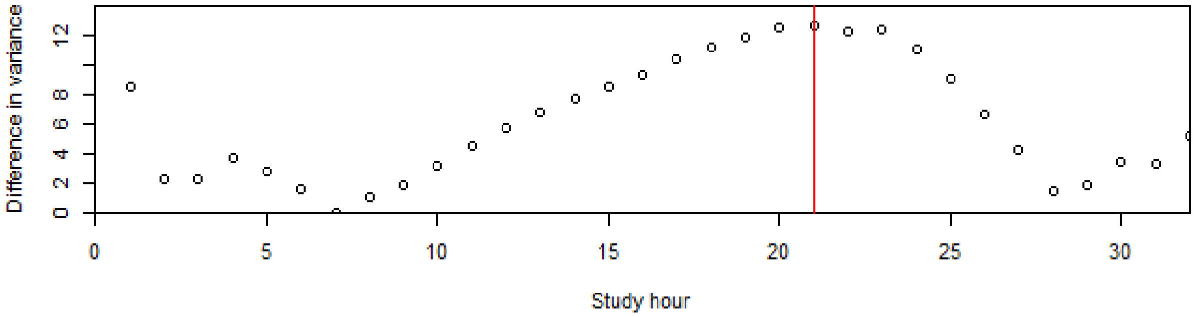

C_leu18: 4 hr

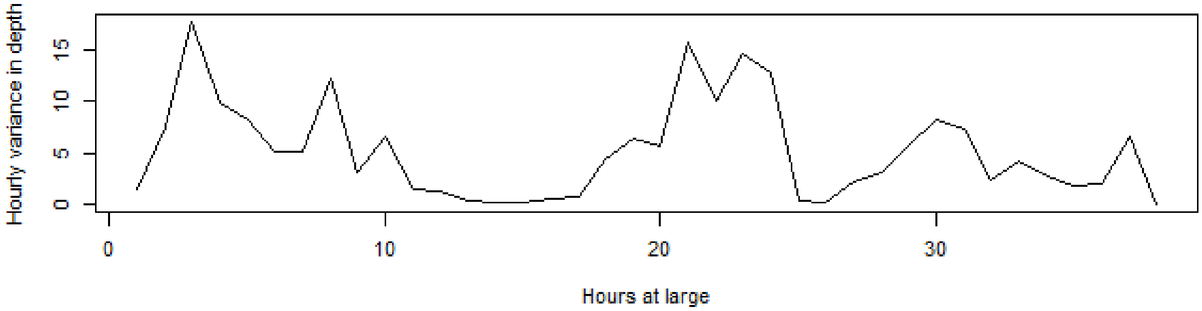

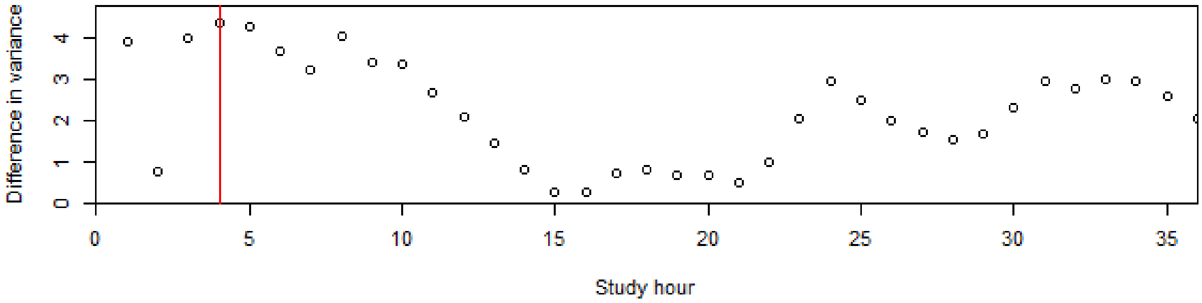

C_leu19: 7 hr

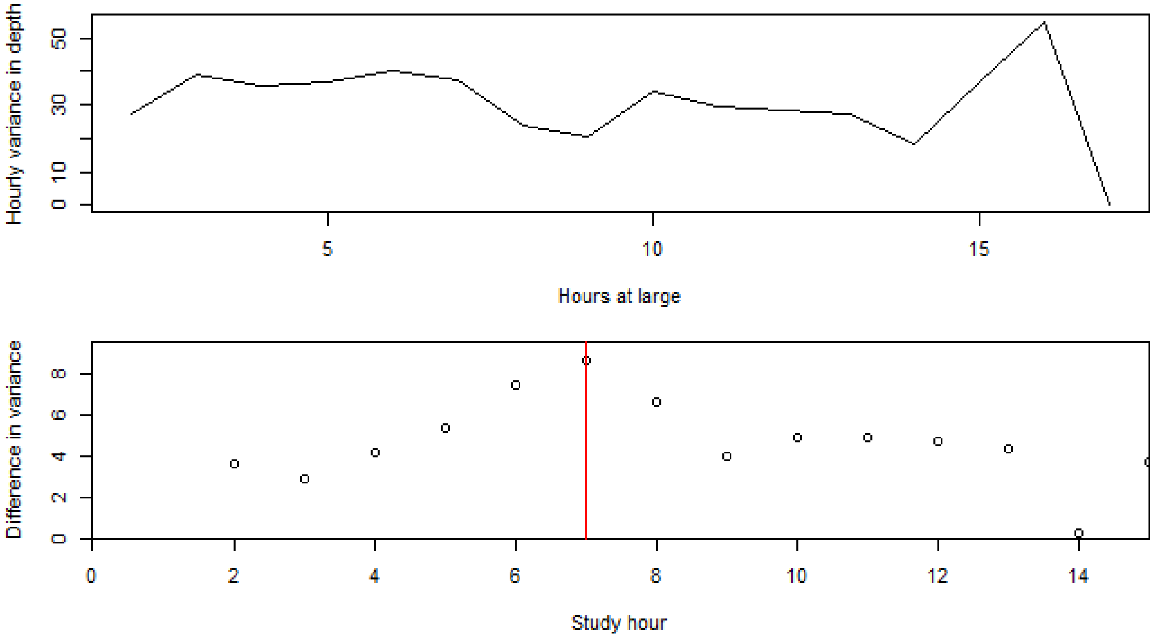

C_lim06: 1 hr

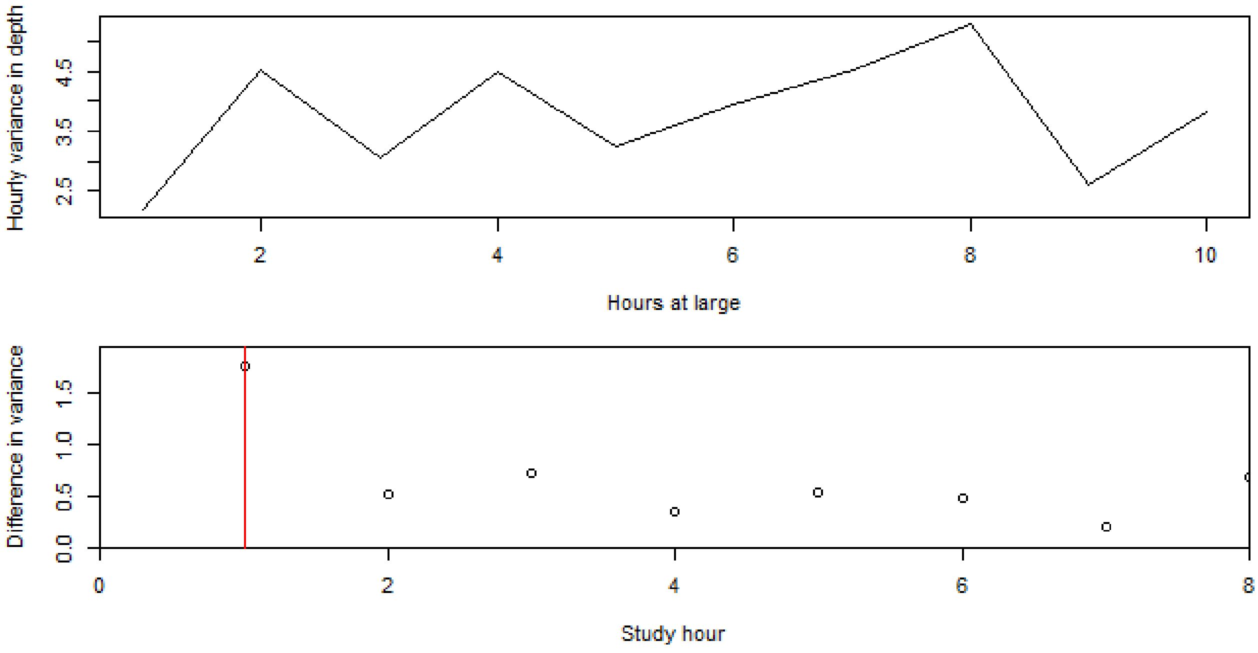

C_lim09: 62 hr

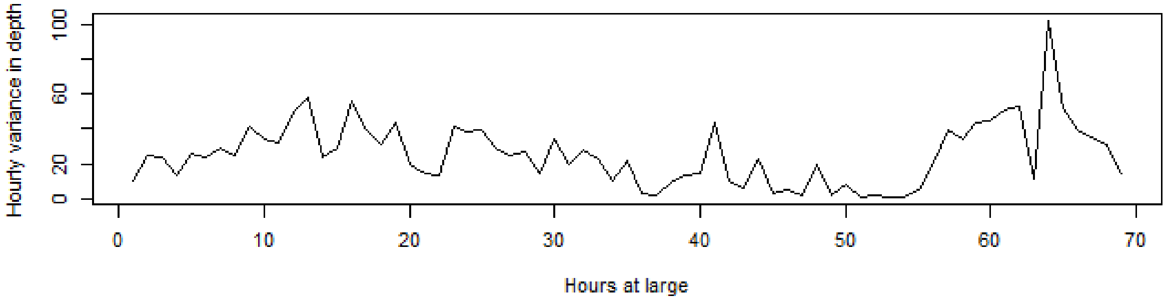

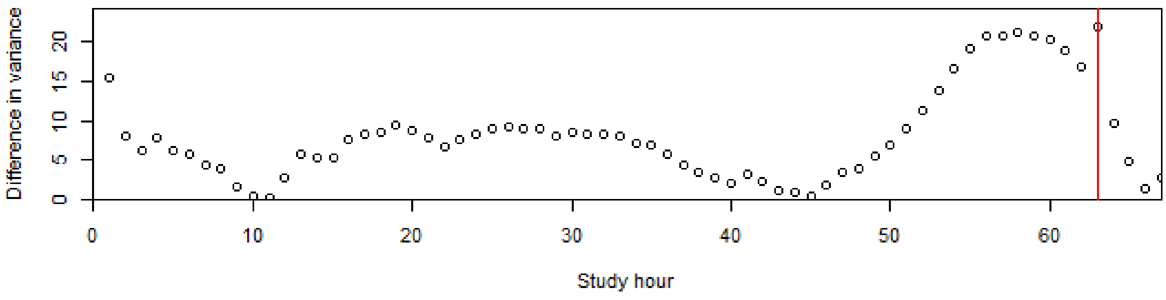

C_lim10: 74hr

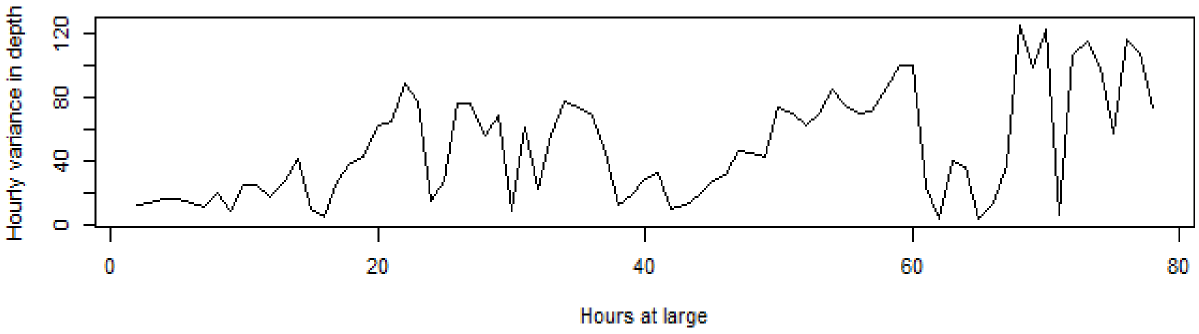

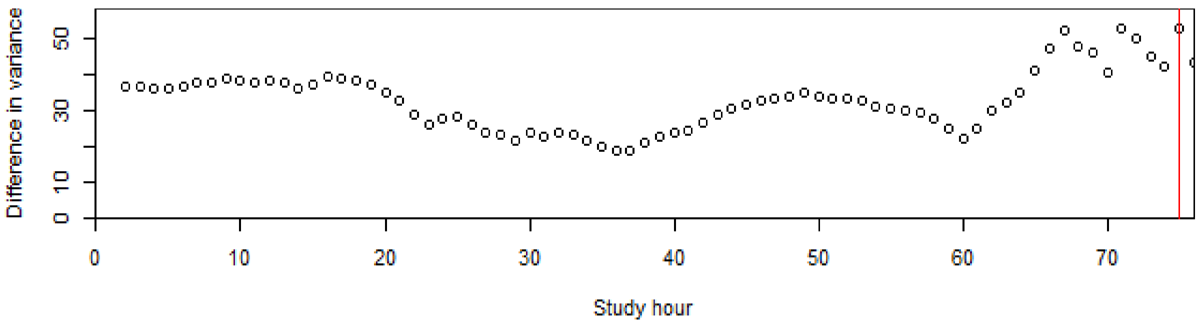

C_lim15: 2 hr

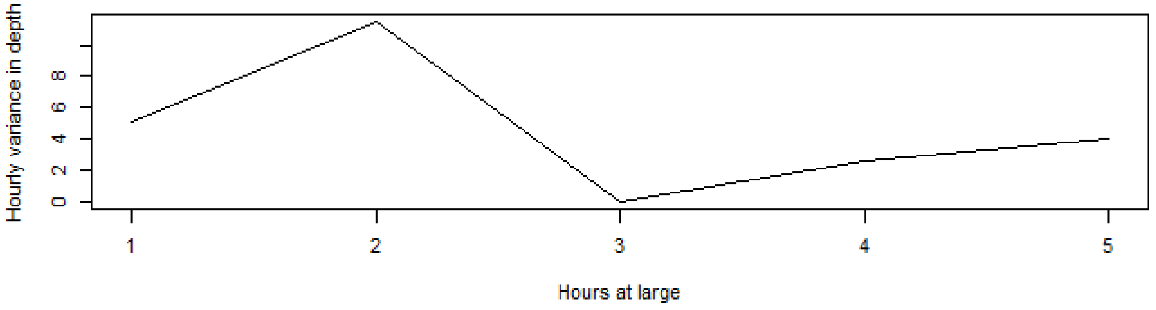

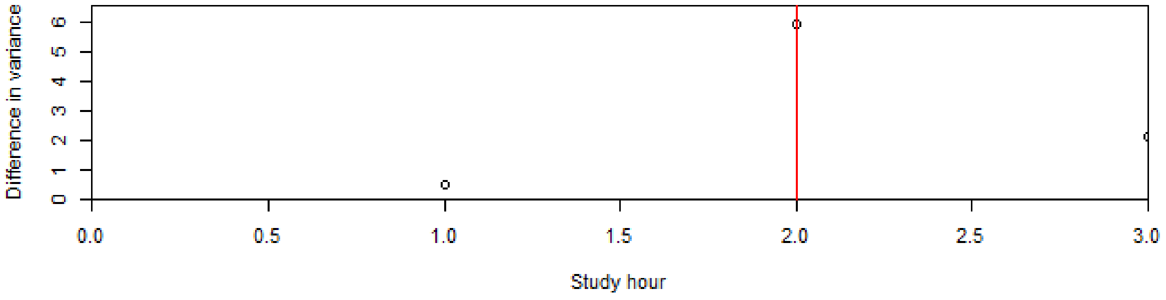

G_cuv06: 9 hr

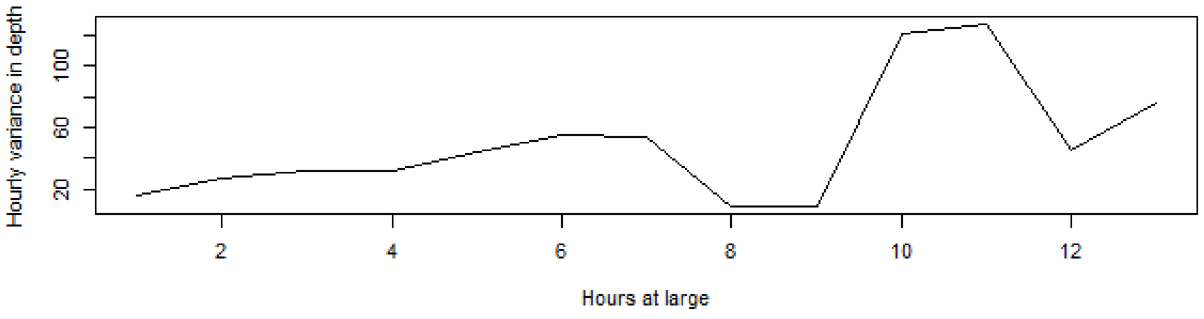

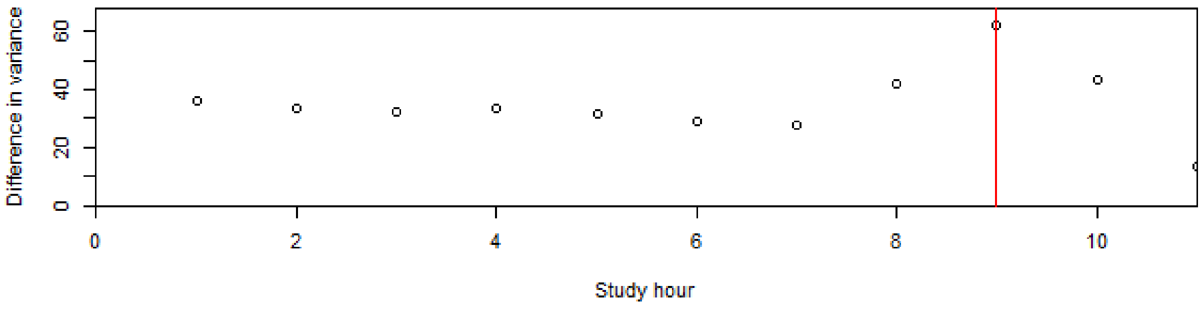

PSAT Hourly Dive Variance

G_cuv05: 20 hr

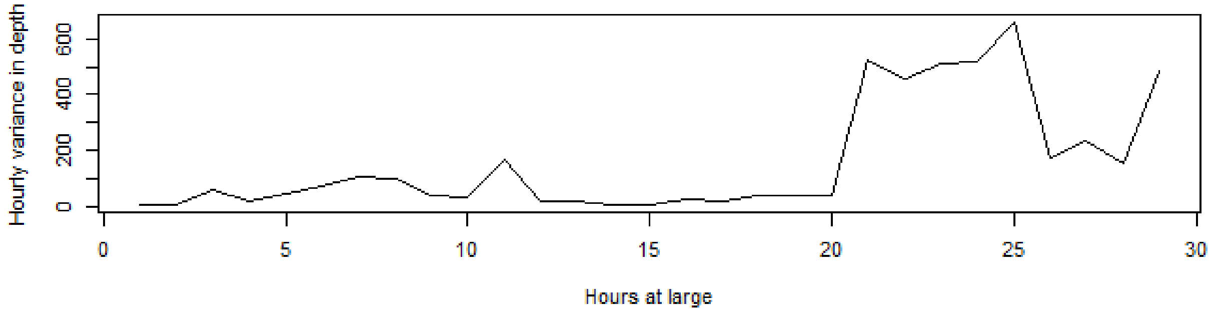

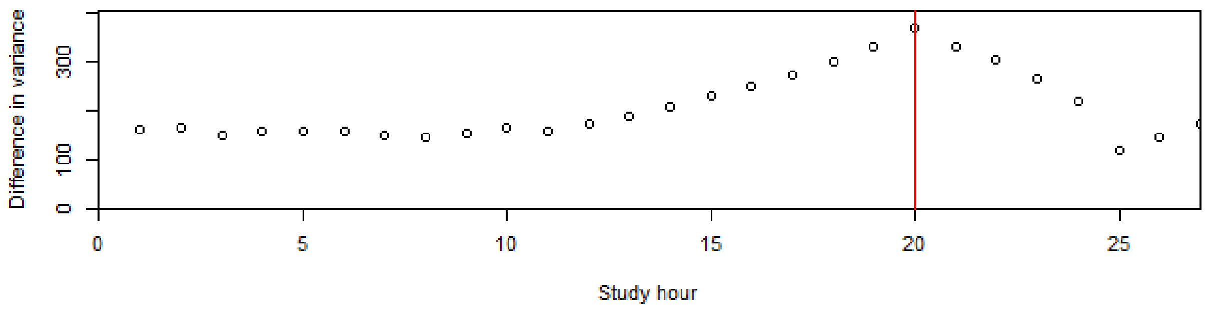

S_mok02: 9hr

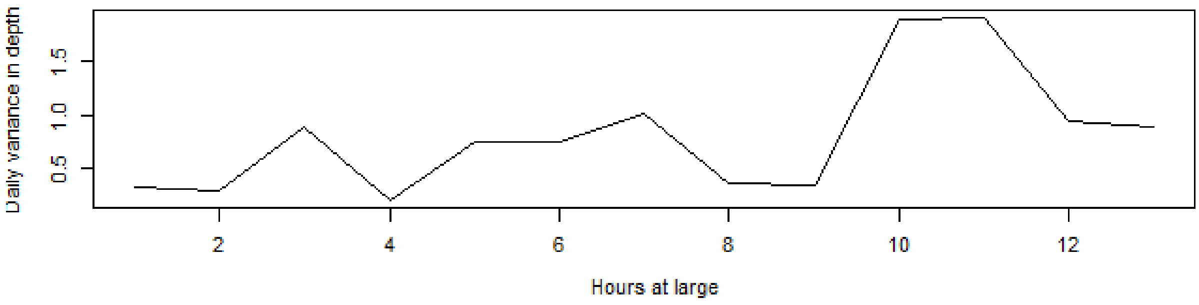

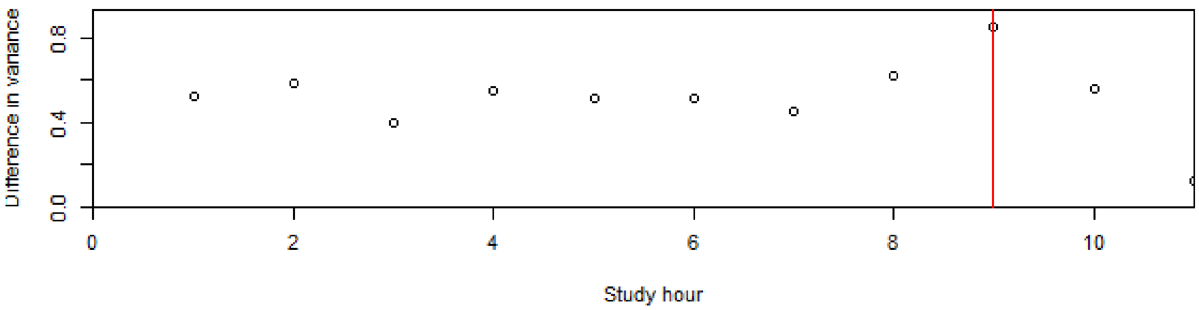

C_leu04: 4 hr

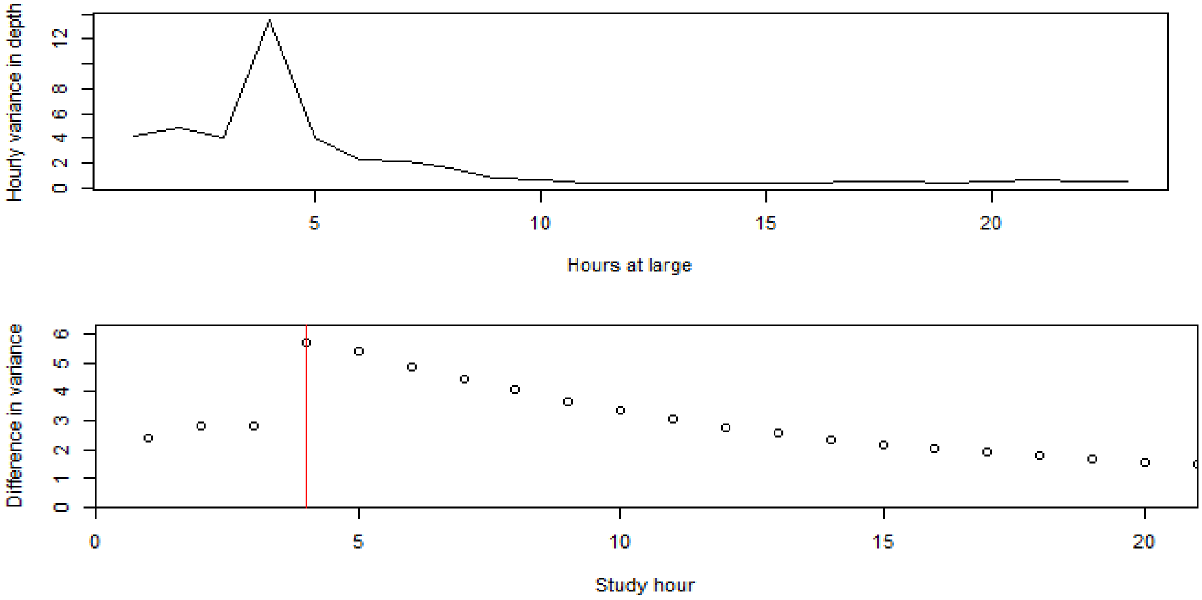

C_leu06: 23 hr

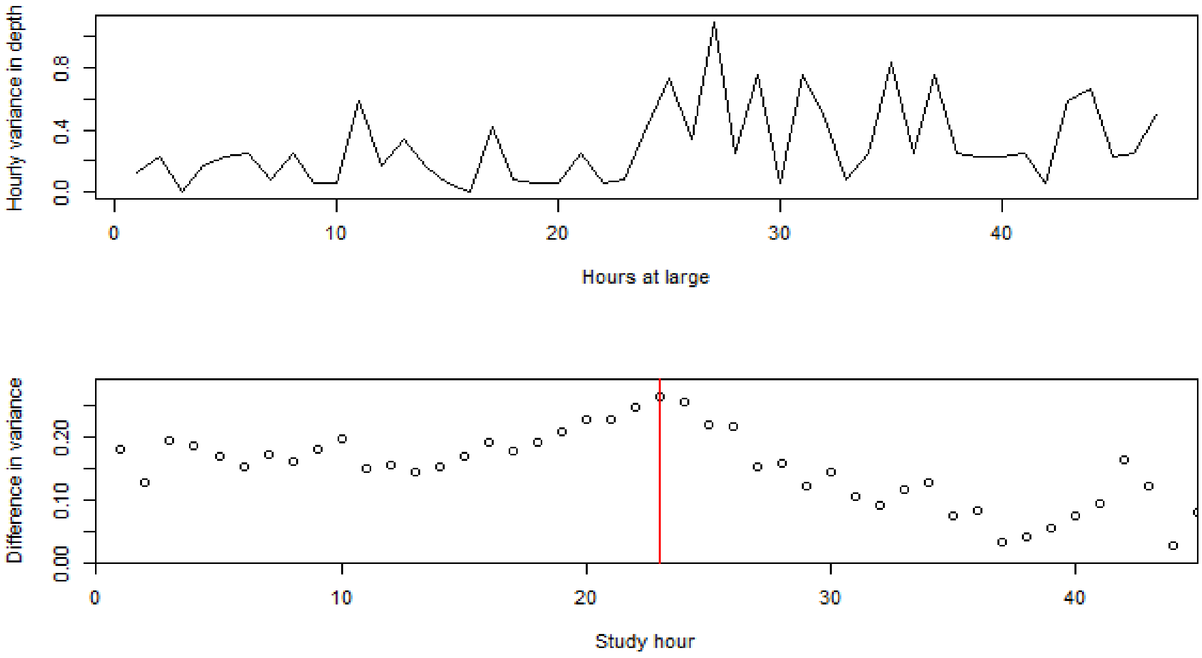

C_leu07: 12 hrs

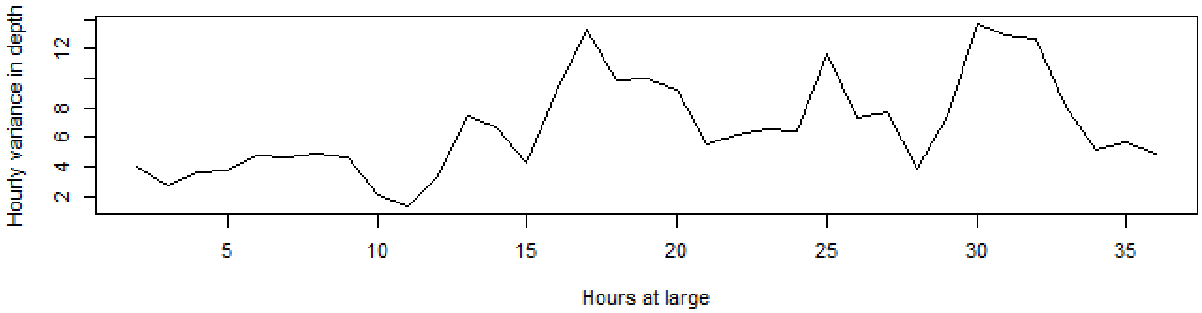

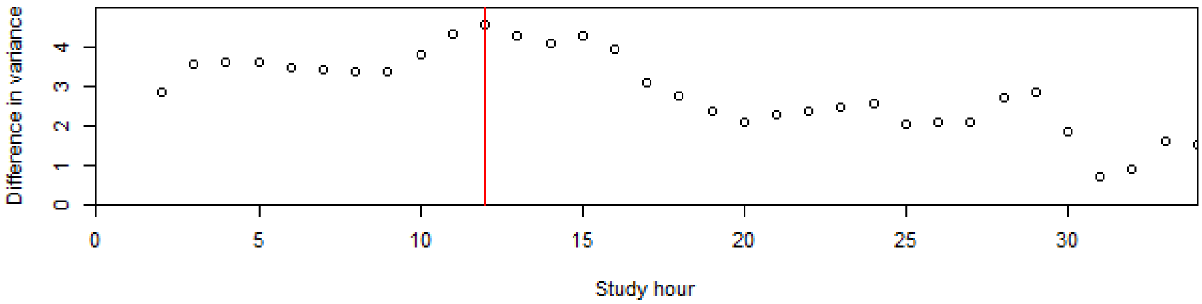

C_leu08: 1 hr

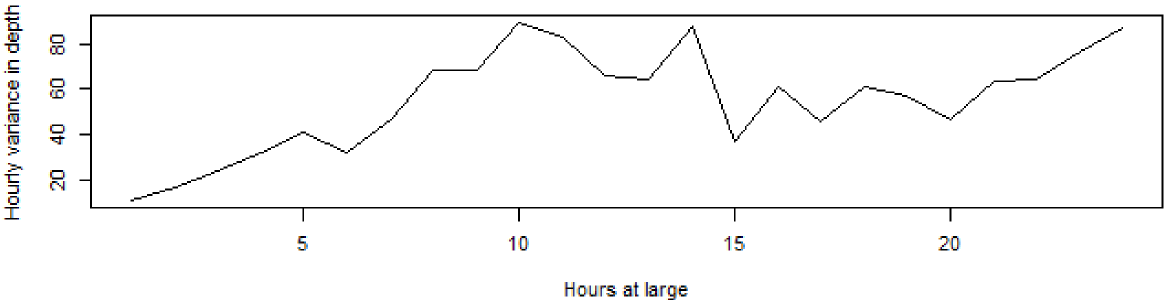

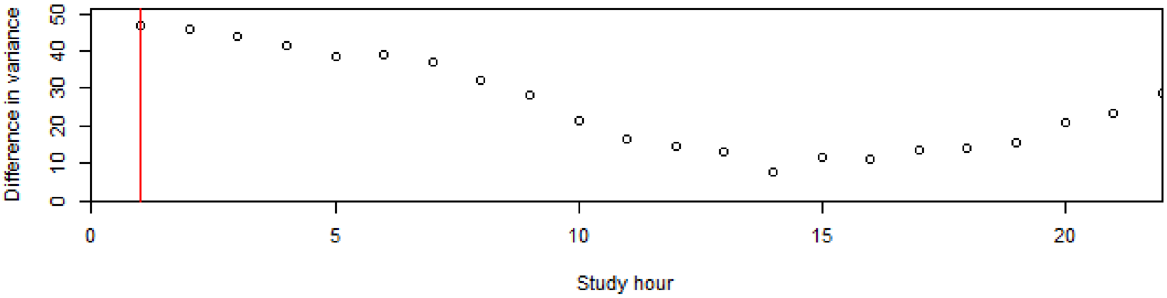

C_lim03: 9 hr

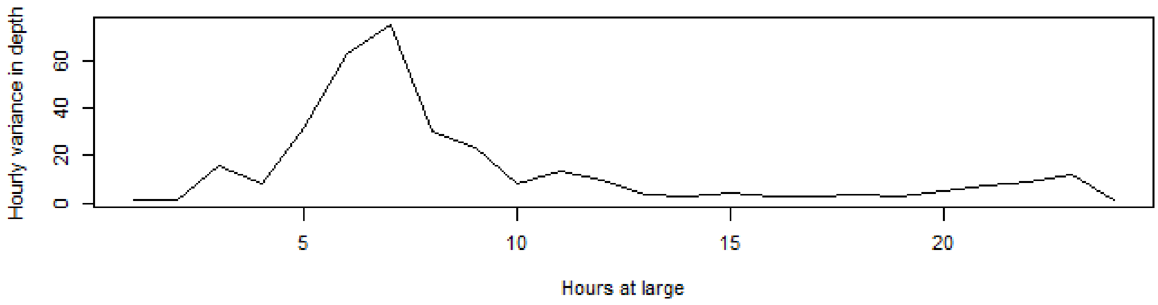

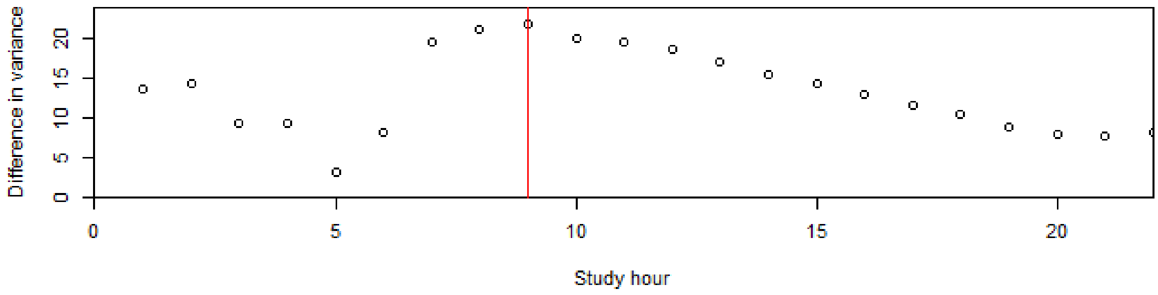

C_lim05: 8 hr

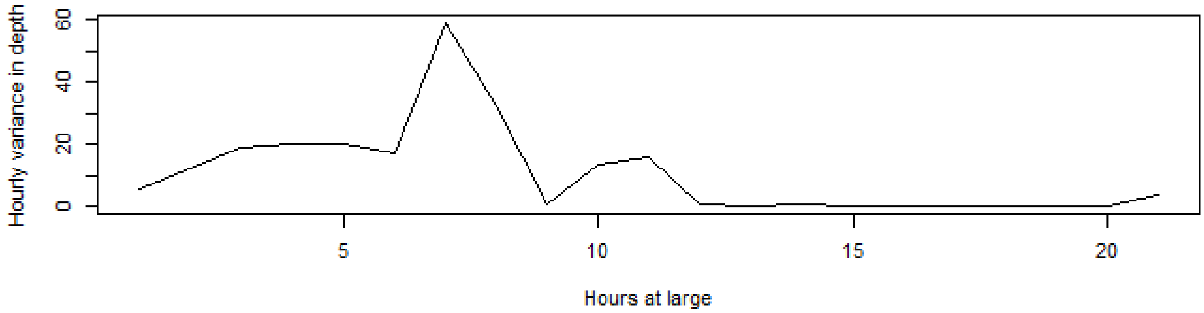

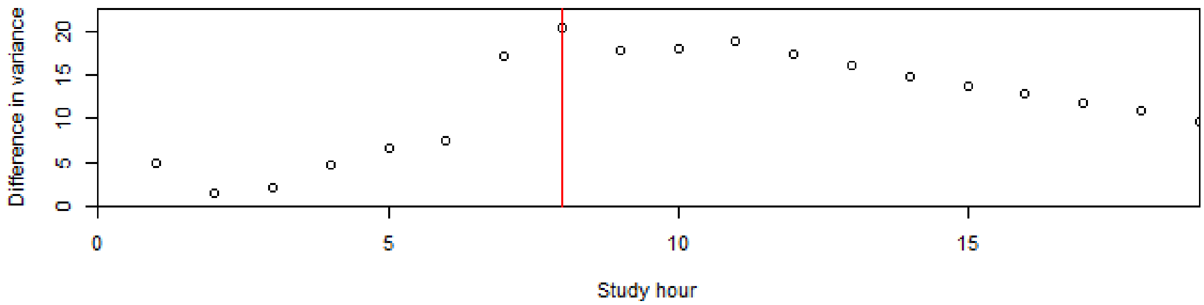

Supplement: S1 File — Includes all relevant figures with respect to recovery period analysis, including species specific tailbeat frequency and overall dynamic body acceleration calculated from ADL tags, hourly dive variance for all sharks that survived capture and release. (PDF) [file pone.0281441.s001.pdf]
