# Supplementary material for: Variable post-release mortality in common shark species captured in Texas shore-based recreational fisheries
Source: PLoS One. 2023 Feb 13;18(2):e0281441. doi: 10.1371/journal.pone.0281441 (PMC9925081; doi:10.1371/journal.pone.0281441)

Supplemental Information 1: PSAT + ADL Time Series

PSAT

Time series from confirmed mortalities

Temperature, depth, a light level plots of shark post-release mortalities. Method of data acquisition and detailed descriptions of each profile and animal fate explained above graph.

S_mok01: Immediately after being tagged, shark went to bottom in 30 minutes where it remained for 11 hours at constant depth, indicating **mortality.** The tag was then ingested, indicated by low light level and temperature variation for 5 consecutive days while depth changed. The tag was regurgitated on 26 October, indicated by large light and temperature changes with minimal depth variation around 0-1.5m, where it floated on the surface for the following 2 days

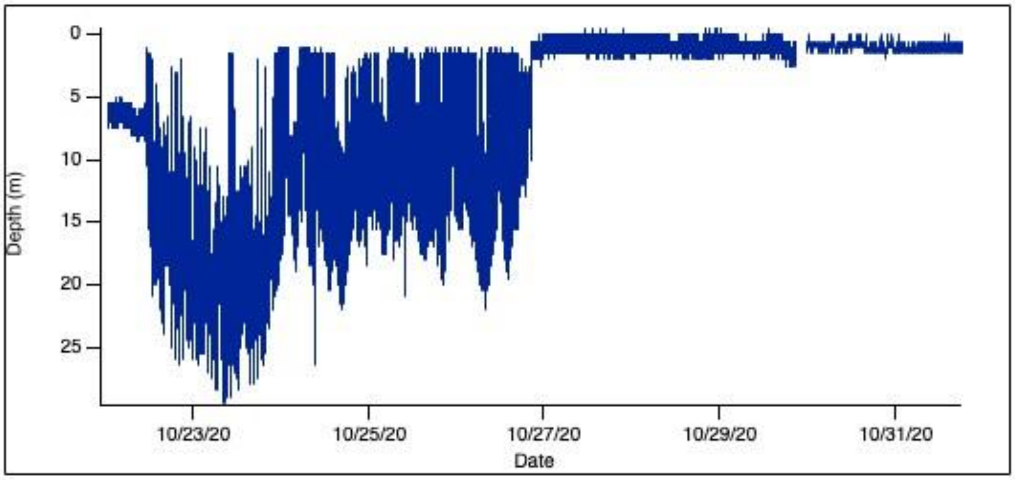

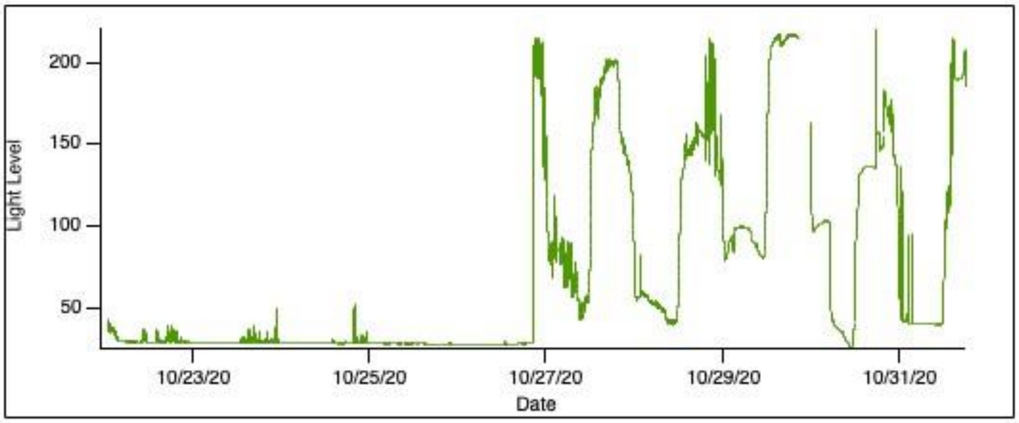

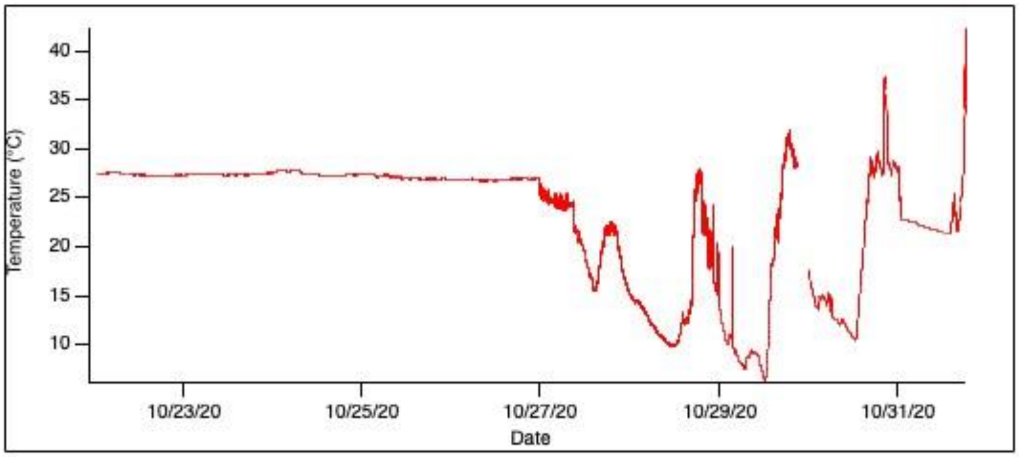

S_mok01: sPAT tag transmitted daily changes in light intensity, and daily min and maximum temperatures and depth values for the full deployment.

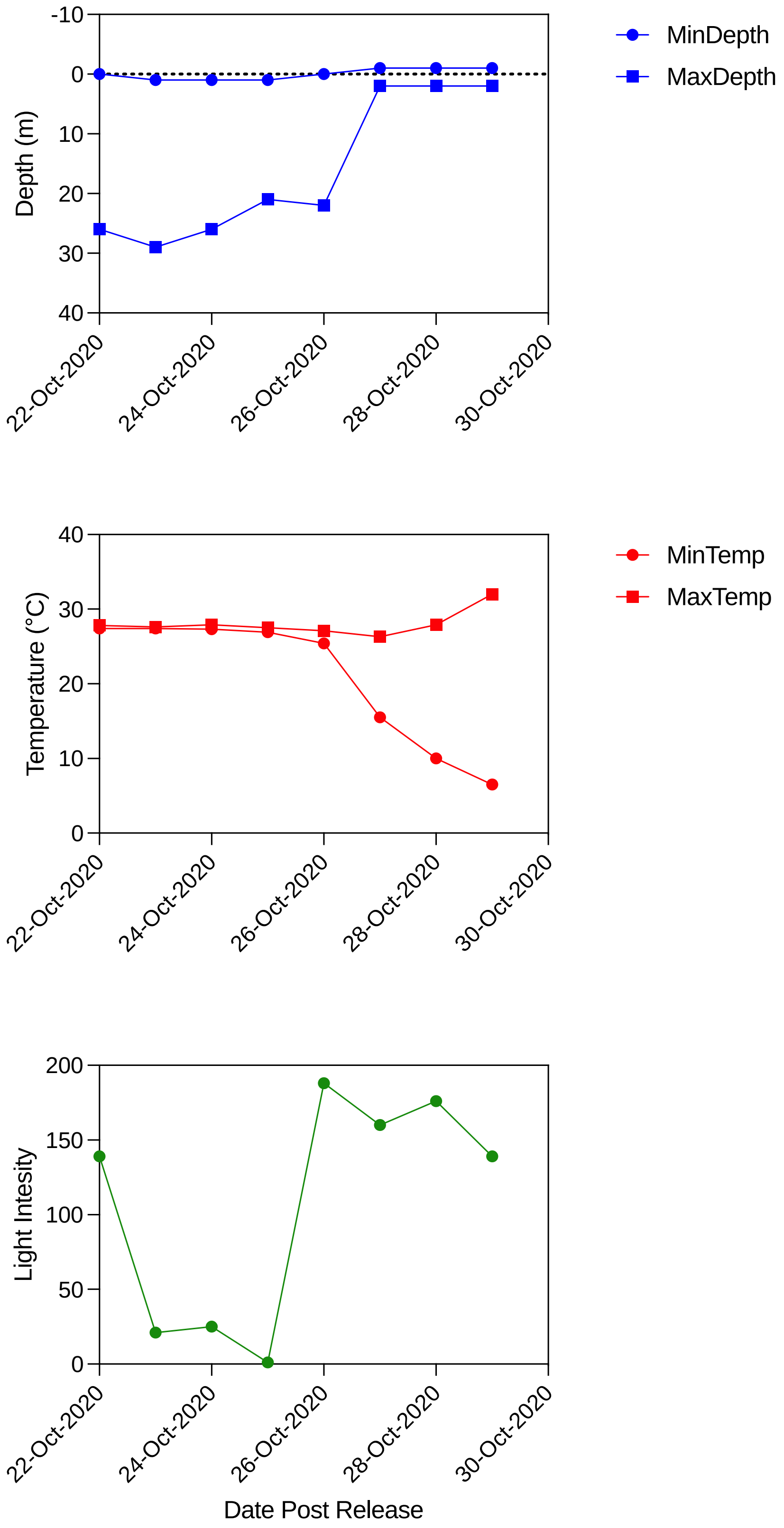

C_lim02: PSATLIFE tag recovered with 16 days of archived data reveal a post-release **mortality** 1.25 hours after tagging. The shark spent 75 minutes yo-yoing from the surface to ~4m before settling at 6m for 5 minutes. The tag then popped off the shark and floated to the surface, where it remained going in and out with the tides for 15 days before shutting off. This is indicated by large temperature and light level changes in conjunction with depth showing the tag 2m above sea level for the period following release from the shark.

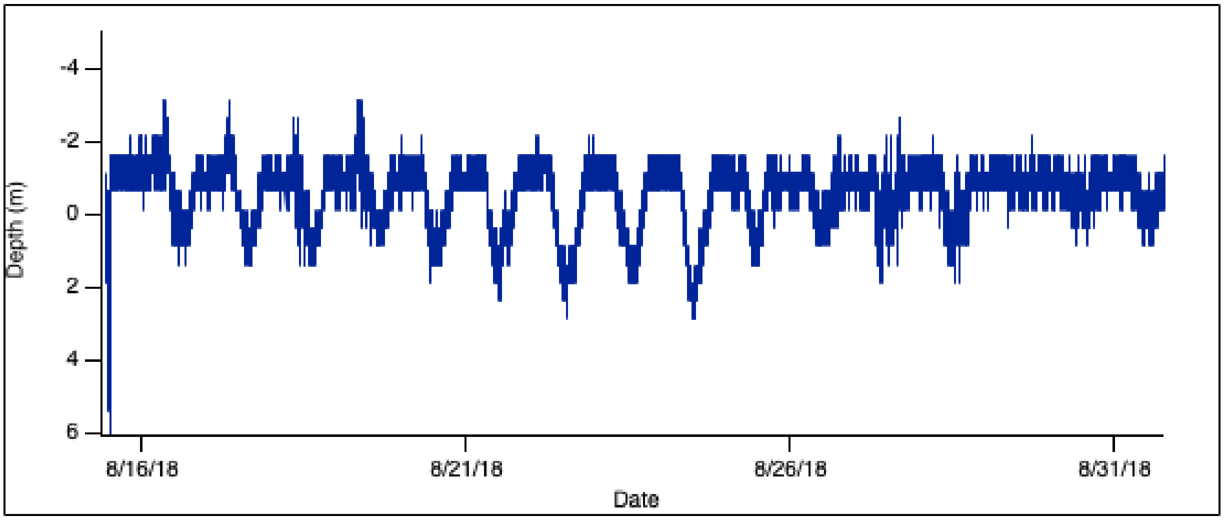

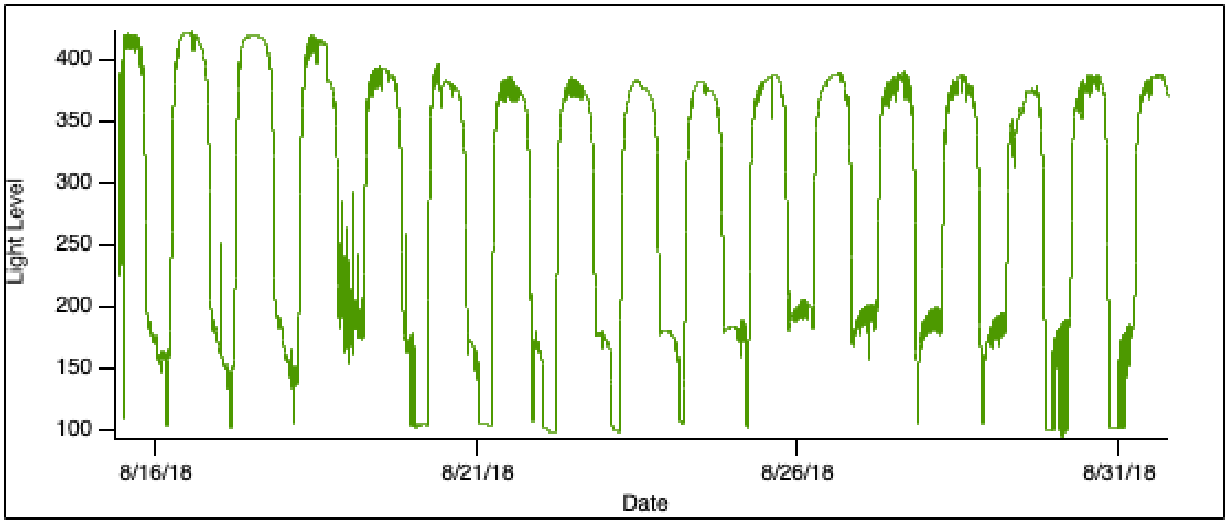

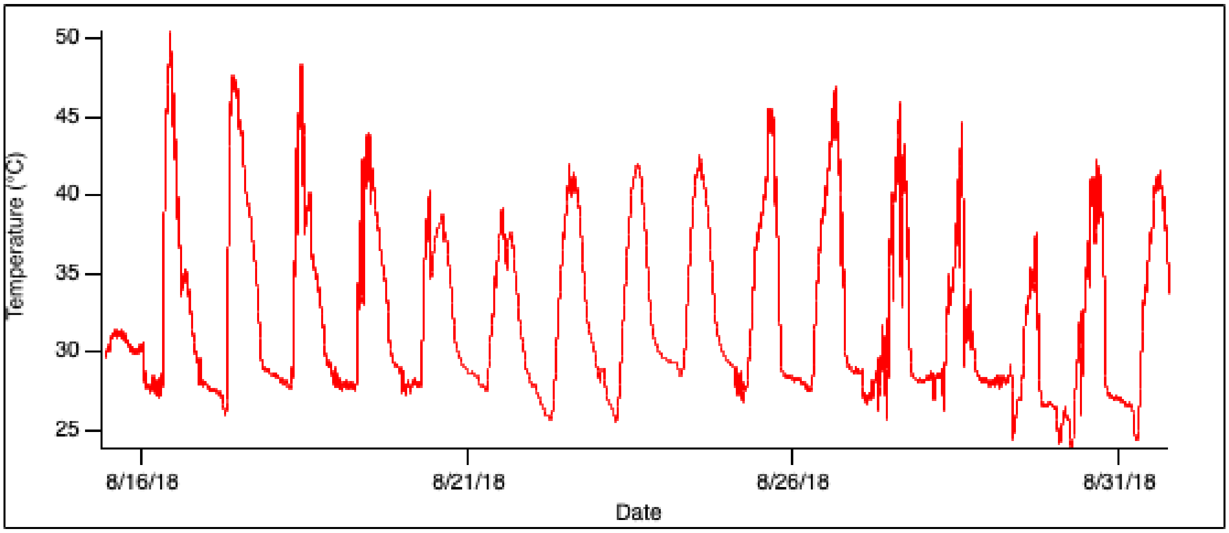

C_lim02: Zoomed in view of the first 1.5 hour of data recording. The shark spent 1.25 hours making its way to the bottom, where it settled at 6m for 5 minutes before the tag released. The length of time spent at depth for an obligate ram ventilating species suggests mortality occurred.

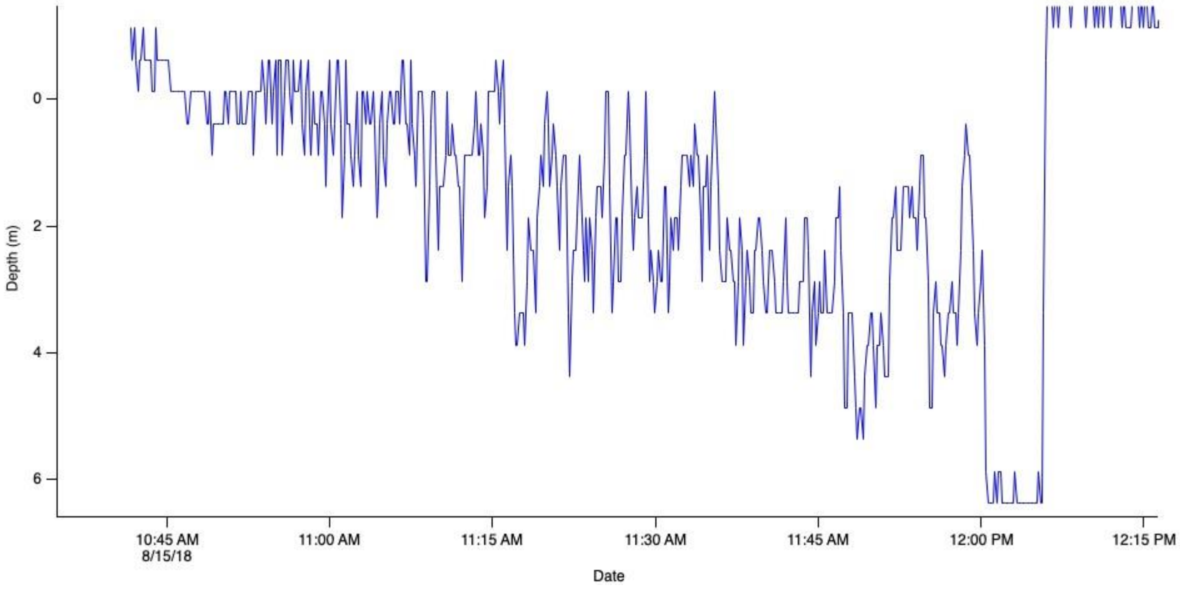

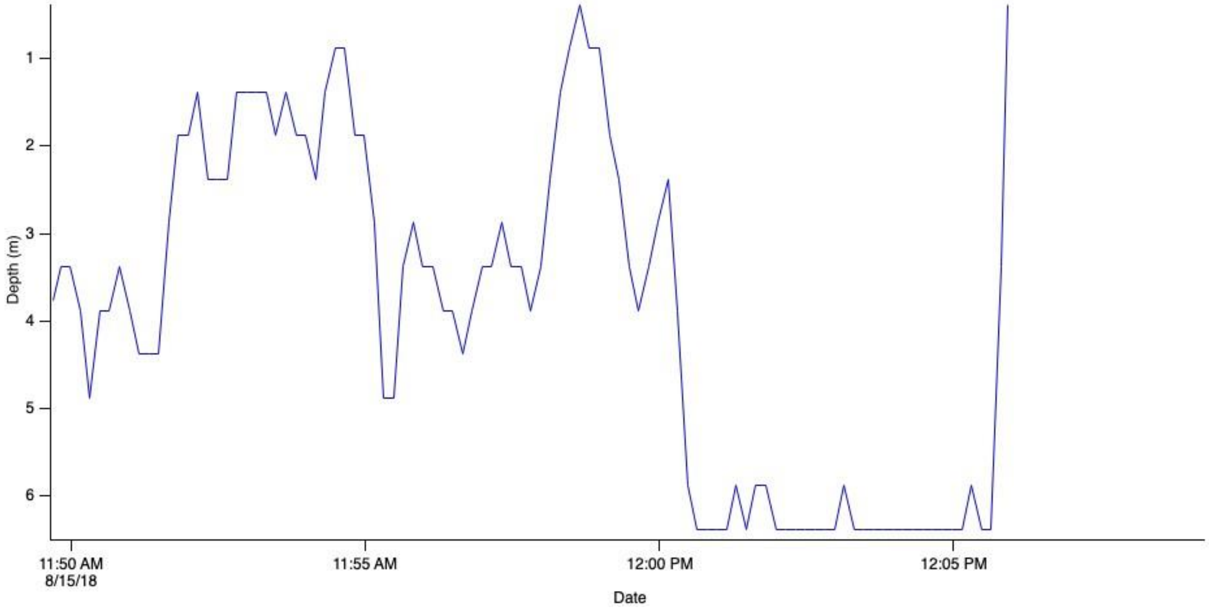

PSAT time series data from survivors that later exhibited mortality characteristics (n=4)

Temperature, depth, a light level plots of sharks that survived capture and release, reestablished normal diving behavior for greater than 10 days, and then exhibited mortality characteristics.

S_mok02: sPAT tag recovered with 16 days of archived data reveal consistent changes in depth, temperature, and light level, indicating **survival.** It is possible the last few days of reduced depth variation could be related to mortality characteristics, resulting in the tag release mechanism being enacted. 14 days of normal diving behavior indicate that even if this period of reduced depth variation was indicative of mortality, it cannot be directly attributed to the stress of capture.

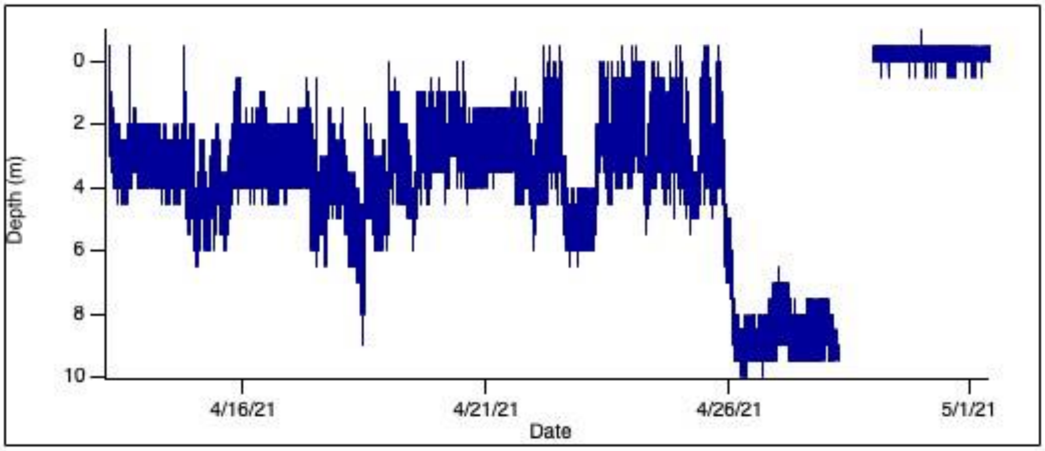

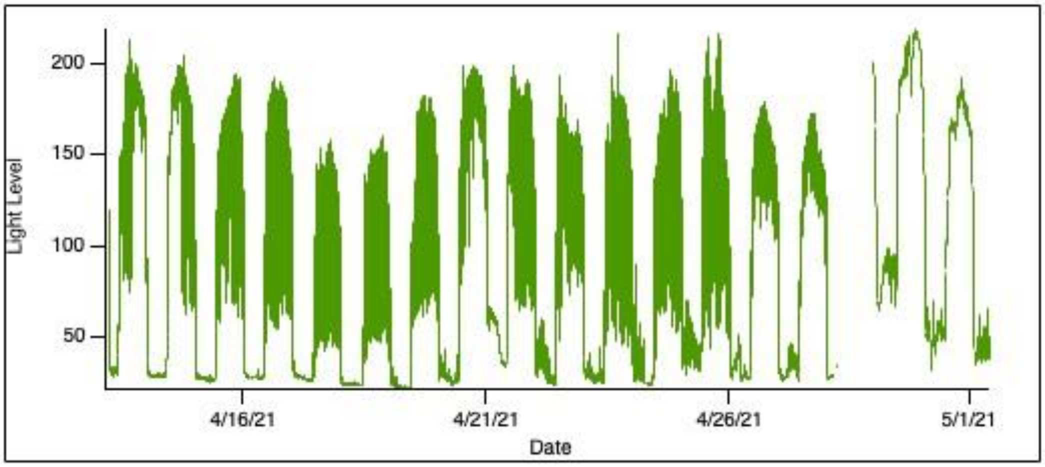

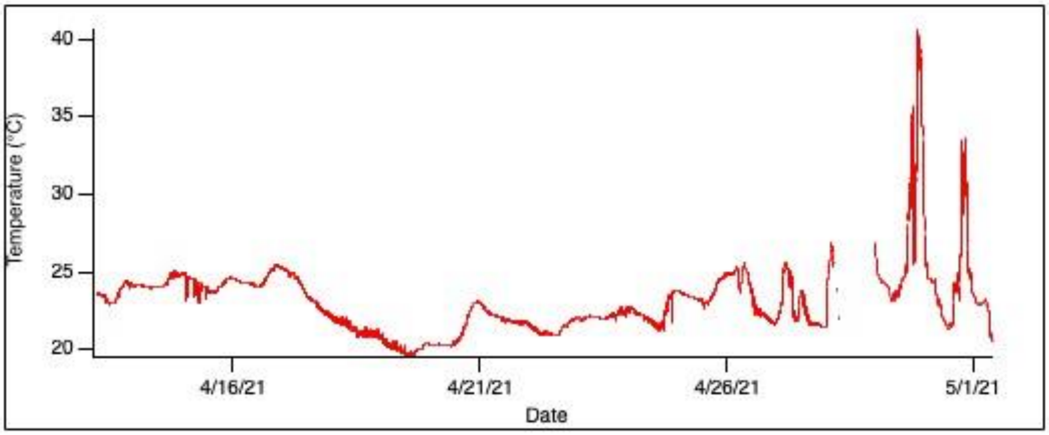

C_lim05: Microwave X-tag transmitted data reveals this individual survived the tagging process and exhibited normal oscillatory diving behavior for 10 days following release. The tag then registers a constant depth for five full days, suggesting a mortality event, before fluctuating between 28-31m for two weeks and finally releasing at its programmed interval. This tag was not pre-programmed to release when constant depth was detected.

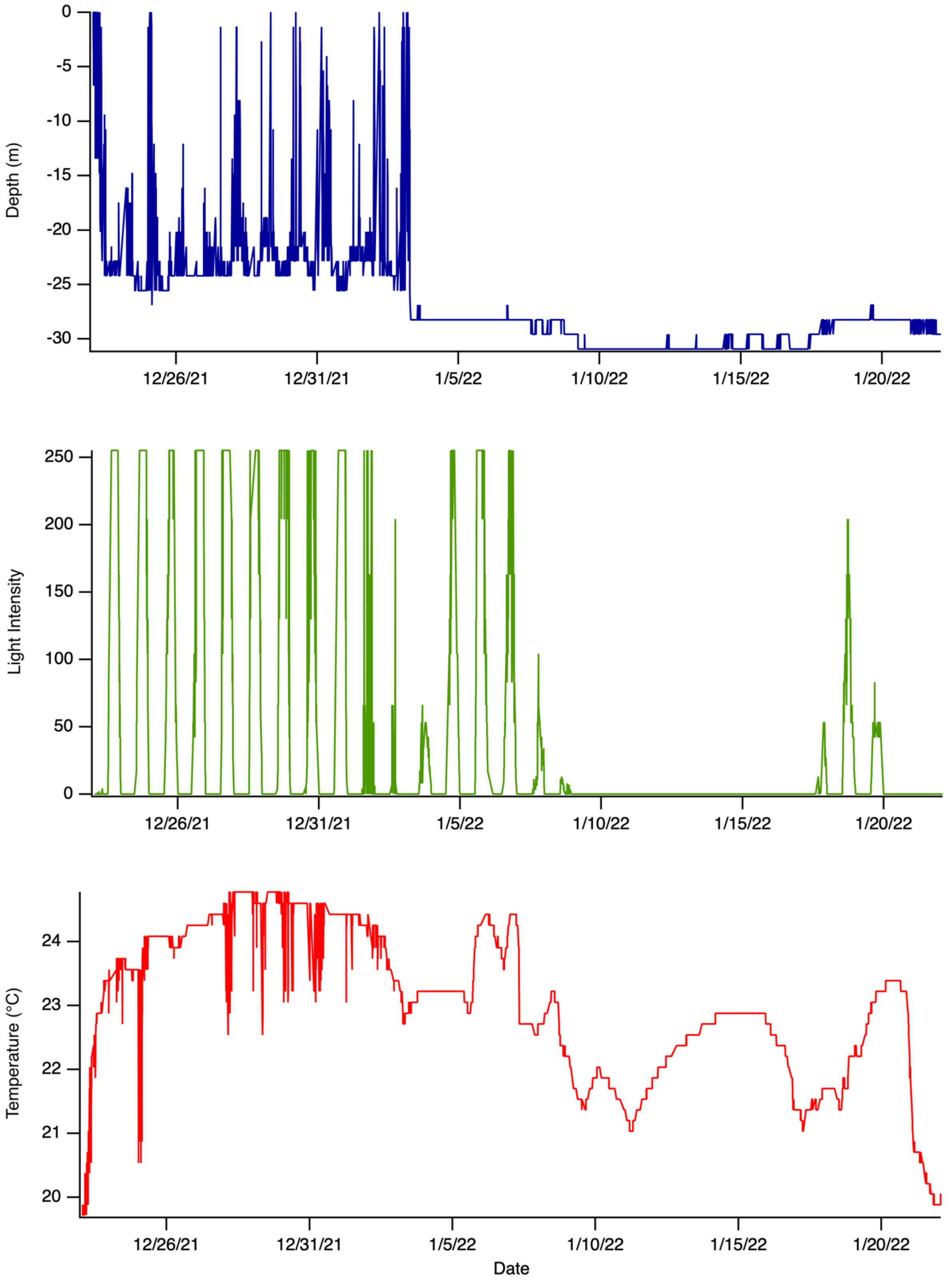

G_cuv01: sPAT tag with 58 days of transmitted data from daily summaries and high-resolution depth data for the final 5 days of recording. Daily summaries reveal normal variations in light, temperature, and depth until 40 days later (9/29) at which point the shark settled at 71m and remained there until recording ceased on 16 October 2020. Normal variations in light, depth, and temperature for this first 40 days indicate **survival**, with a potential natural mortality occurring after tagging.

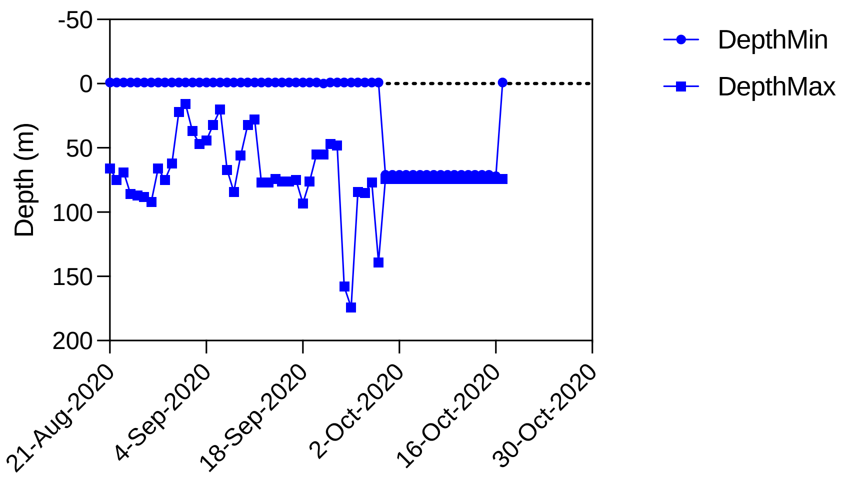

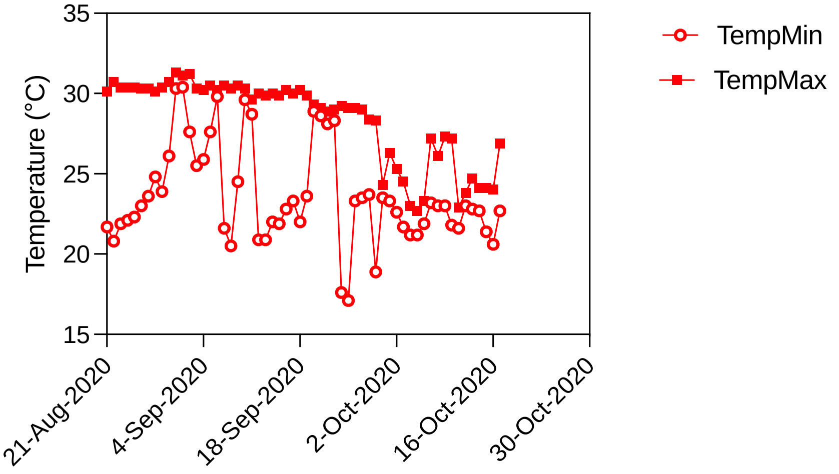

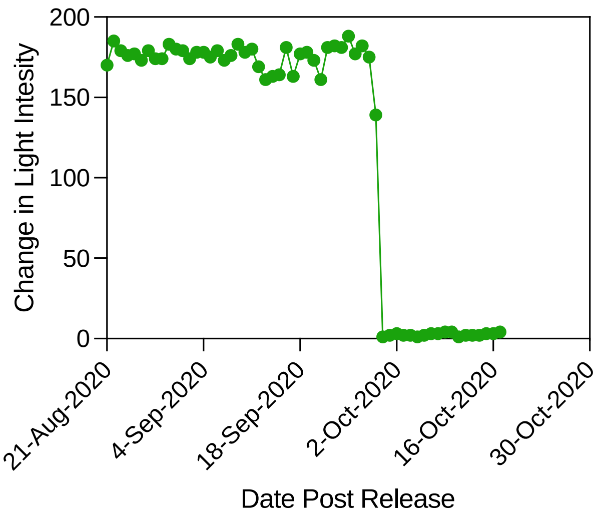

G_cuv05: Mini-PAT tag with 57 days of transmitted data reveal consistent changes in depth, temperature, and light level, indicating **survival.** On 10/22, depth variations cease and depth remains constant for 3 days, initiating the tag release sequence and suggesting mortality. Given almost 2 months of normal movement post-release, this was considered a natural mortality

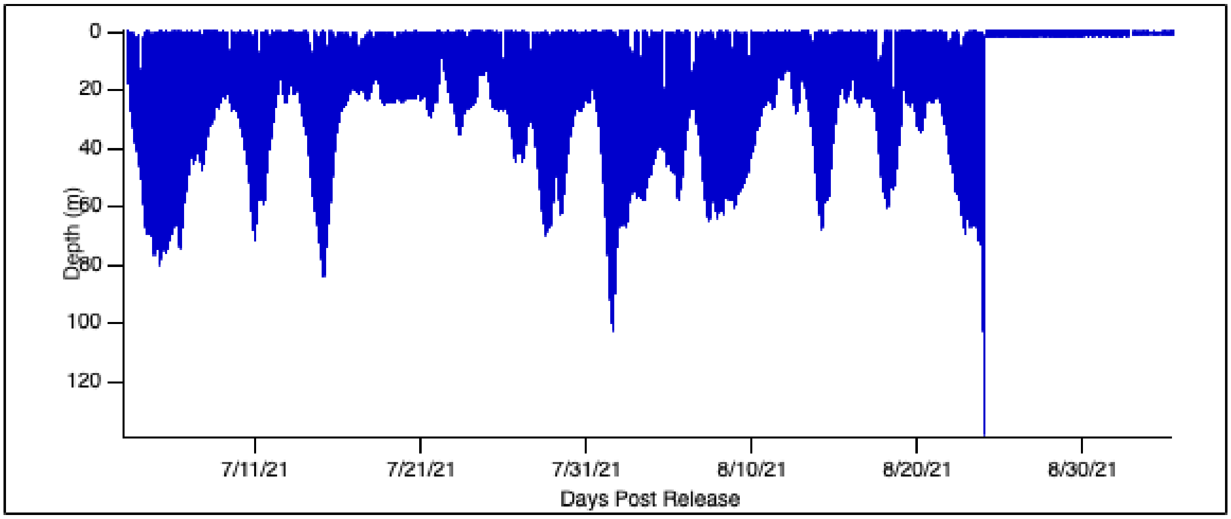

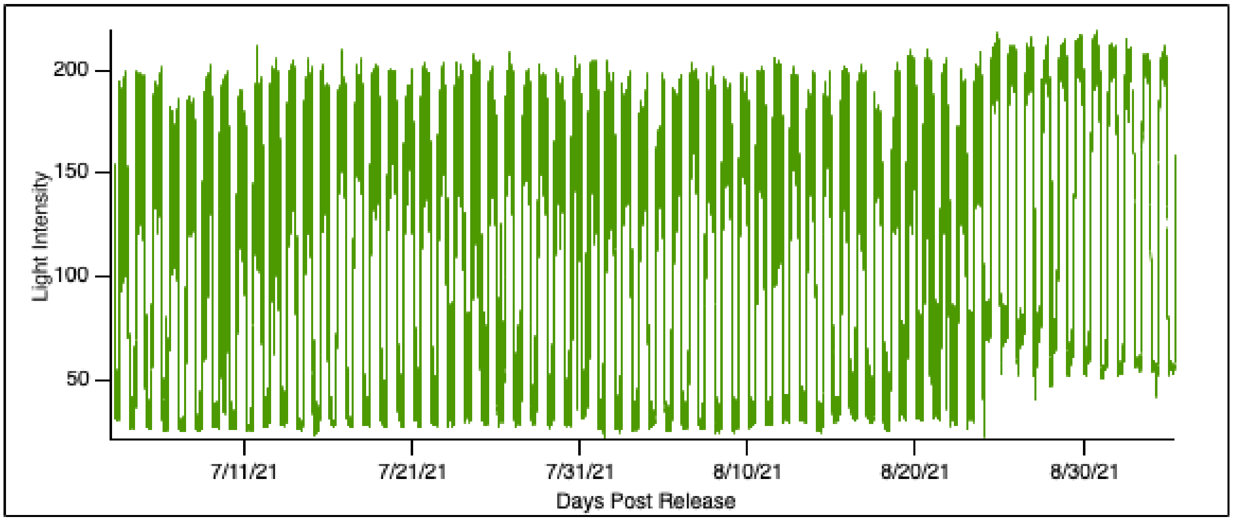

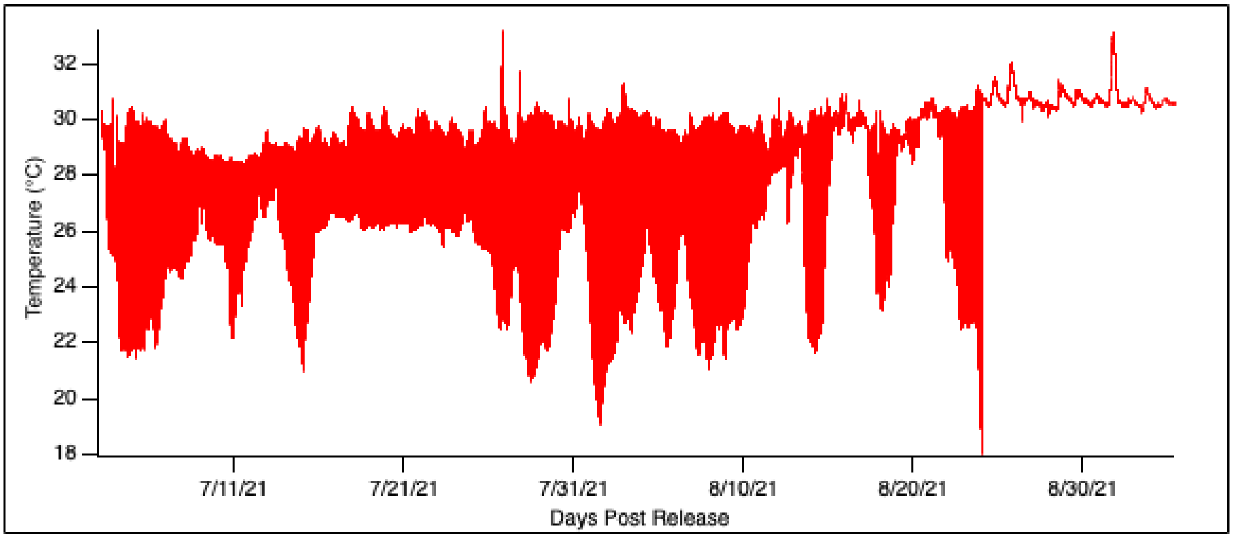

PSAT time series data from confirmed SURVIVORS (N=10)

Temperature, depth, a light level plots of shark post-release survivals. Survival confirmed with consistent changes in light level, depth and temperature, indicating free movement in water column.

C_leu05: PSATLIFE tag with 28 days of transmitted data reveal the shark immediately went to 59m and remained at constant depth for 3.25 hours, indicating a **mortality.** The tag remained at near constant depth for the rest of the deployment, but minimal changes in depth (perhaps due to currents and tidal motion) prevented the release condition to release the tag. The tag released at programmed 28 days, but data transmission was not continuous and patchy with no light level data.

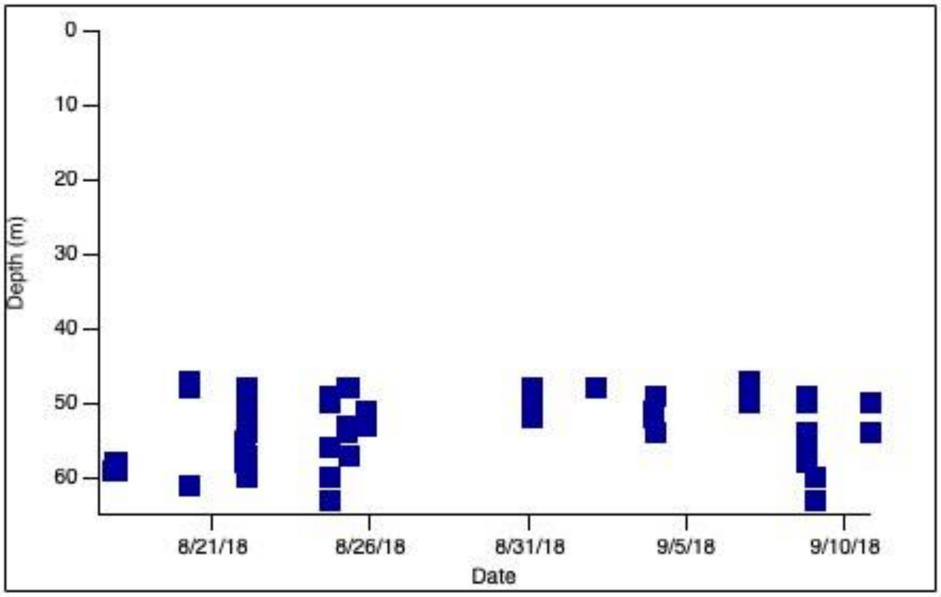

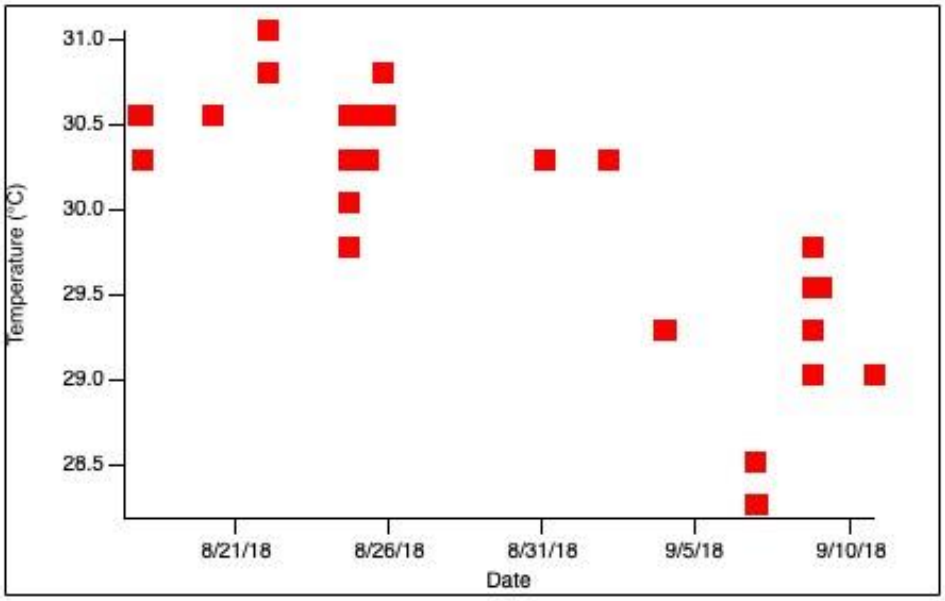

C_leu07: PSATLIFE tag recovered with 28 days of archived data reveal consistent changes in depth, temperature, and light level, indicating **survival.**

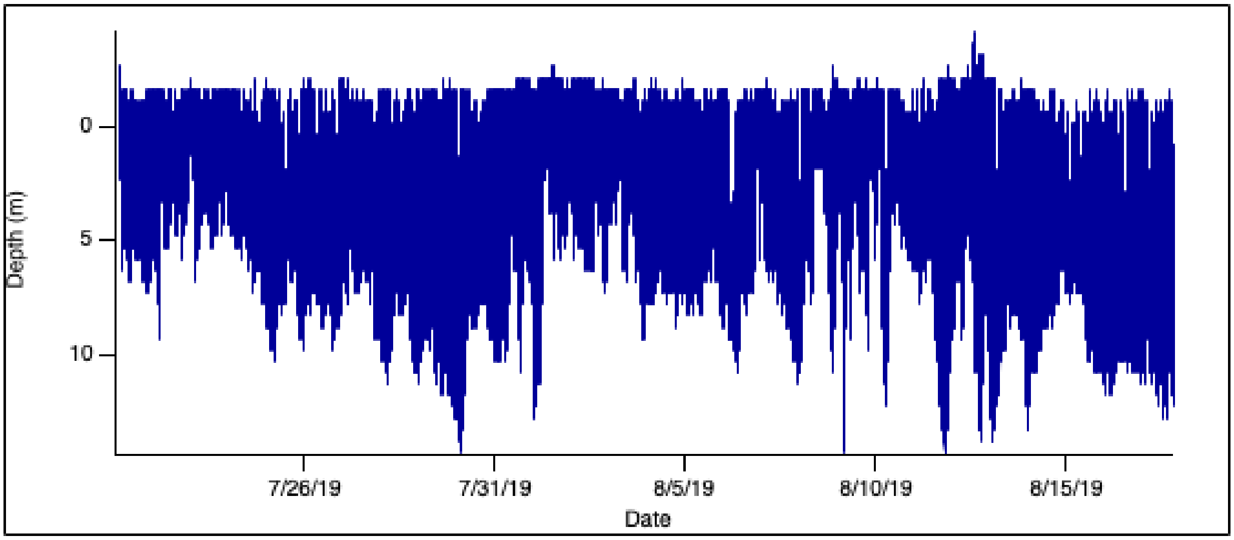

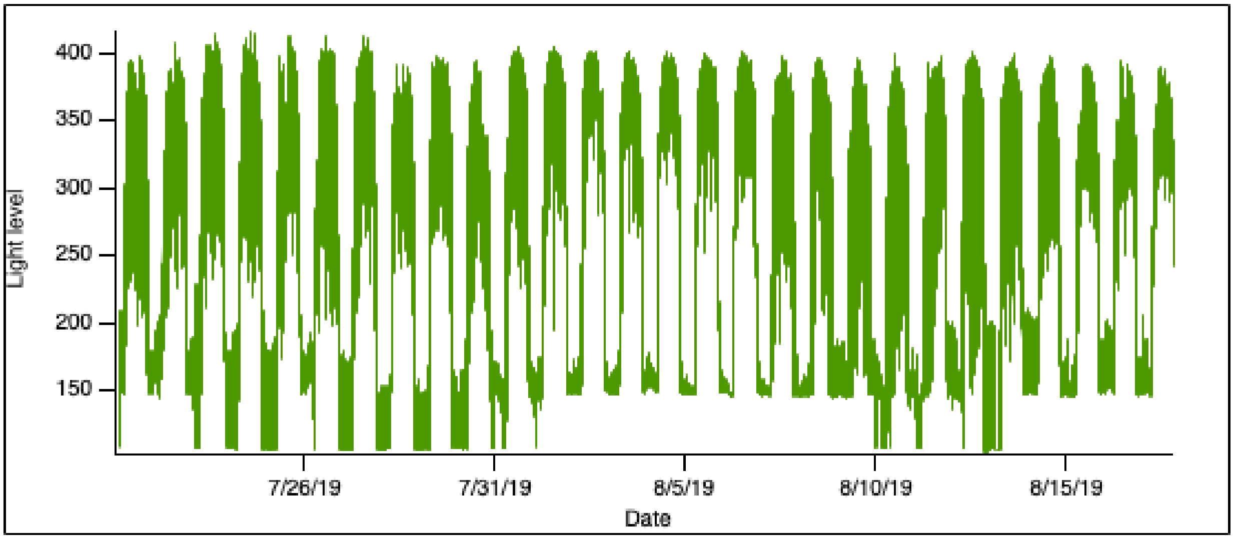

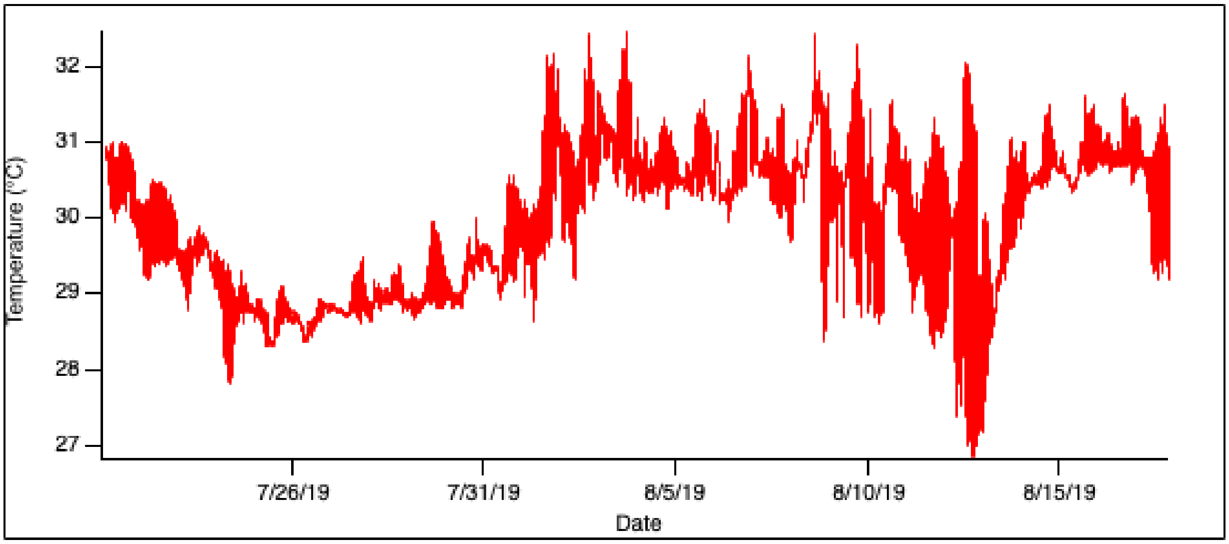

G_cuv02: sPAT tag with 60 days of transmitted data from daily summaries. The shark exhibited depth variation between the surface and about 80 meters for the first 30 days. It then made extremely deep dives from the surface to over 700 m depth for another 30 days before the tag released. The tag on this female tiger released over 1000 km to the east of the tagging location.

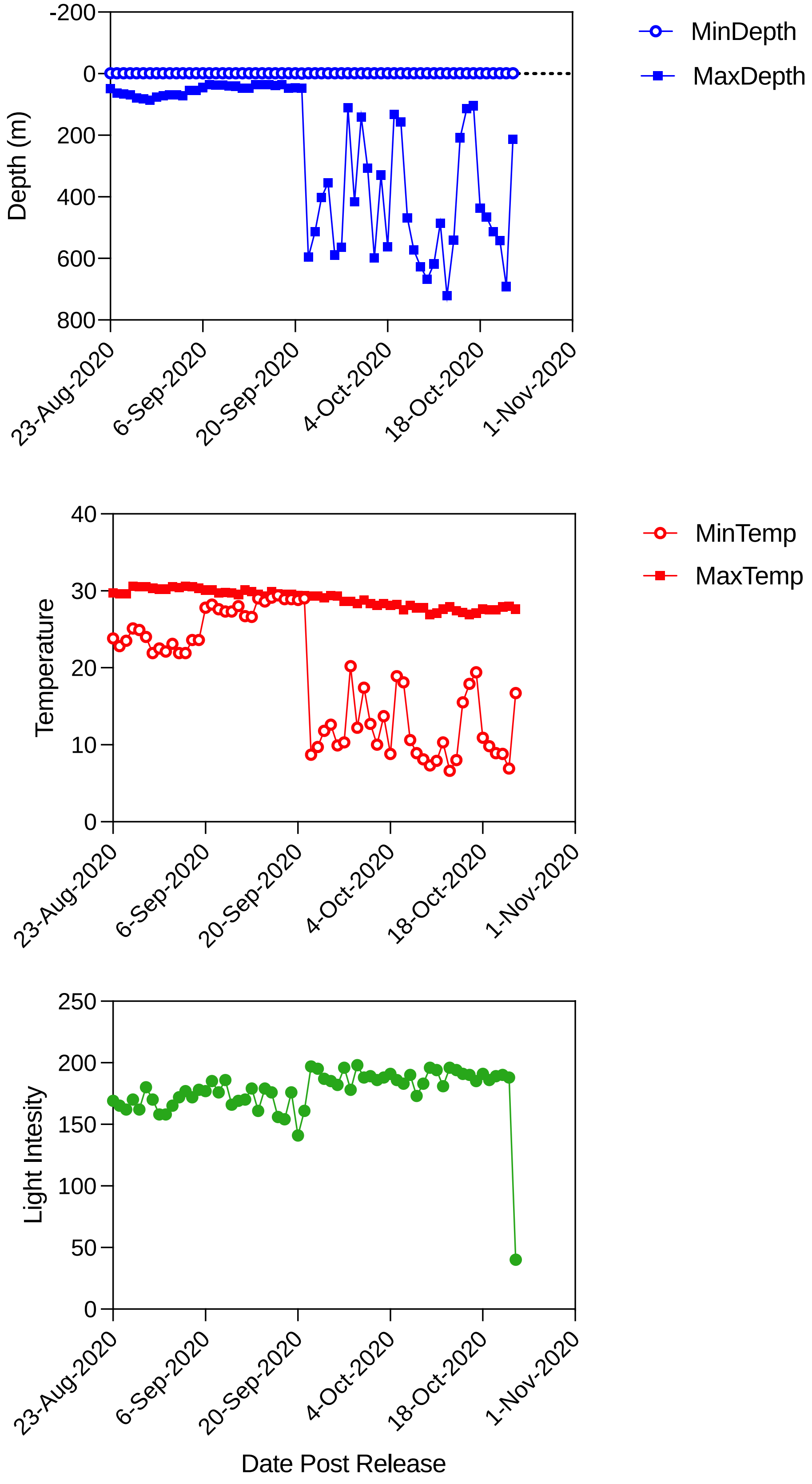

C_leu04: sPAT tag recovered with 10 days of archived data reveal consistent changes in depth, temperature, and light level, indicating **survival.** Daily min/max summaries below

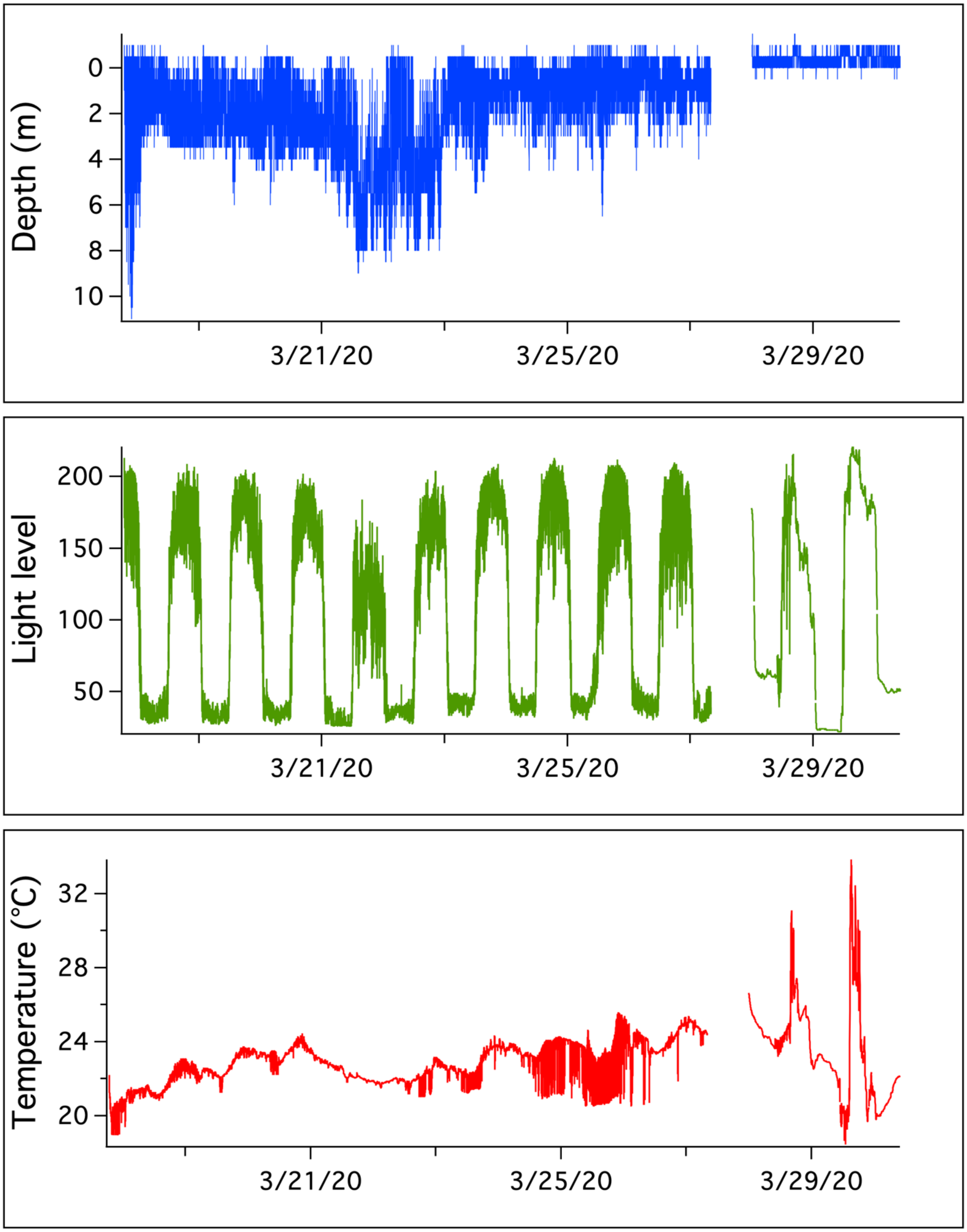

C_leu04: sPAT tag recovered with 10 days of daily changes in depth, temperature, and light indicate **survival**

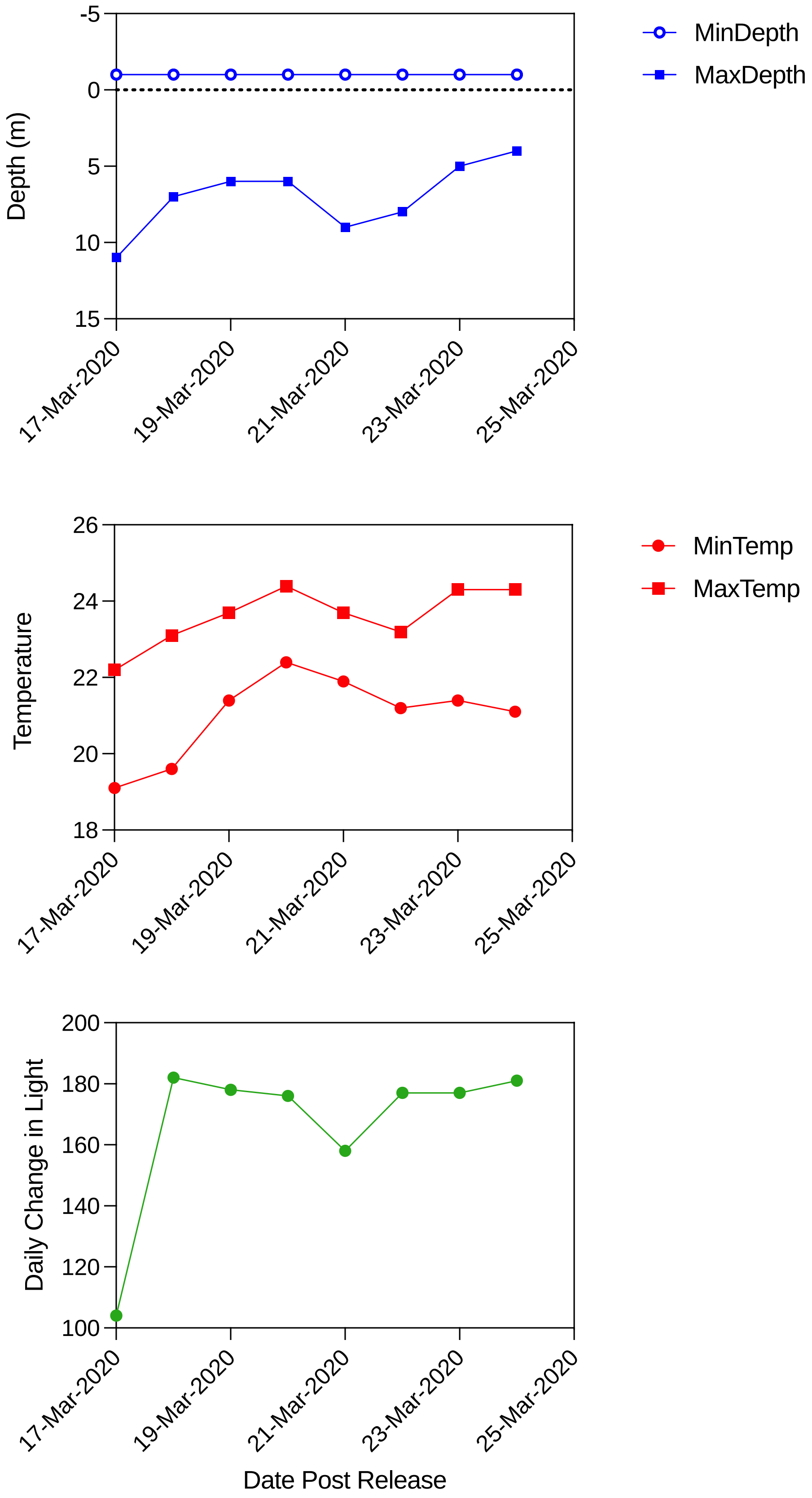

G_cuv03: sPAT tag recovered with 5 days of archived data reveal consistent changes in depth, temperature, and light level, indicating **survival**

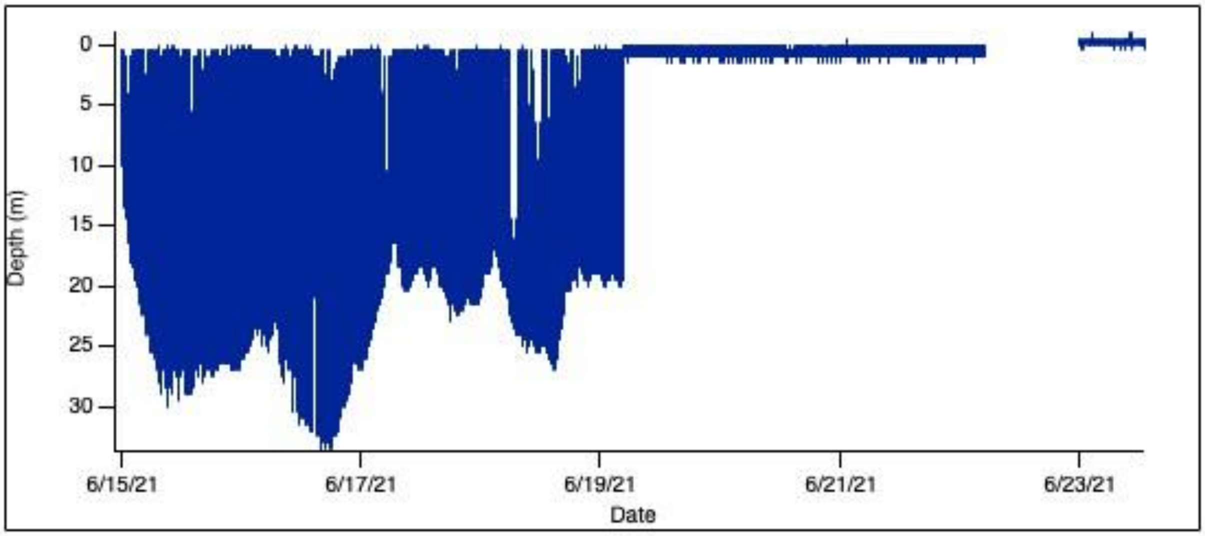

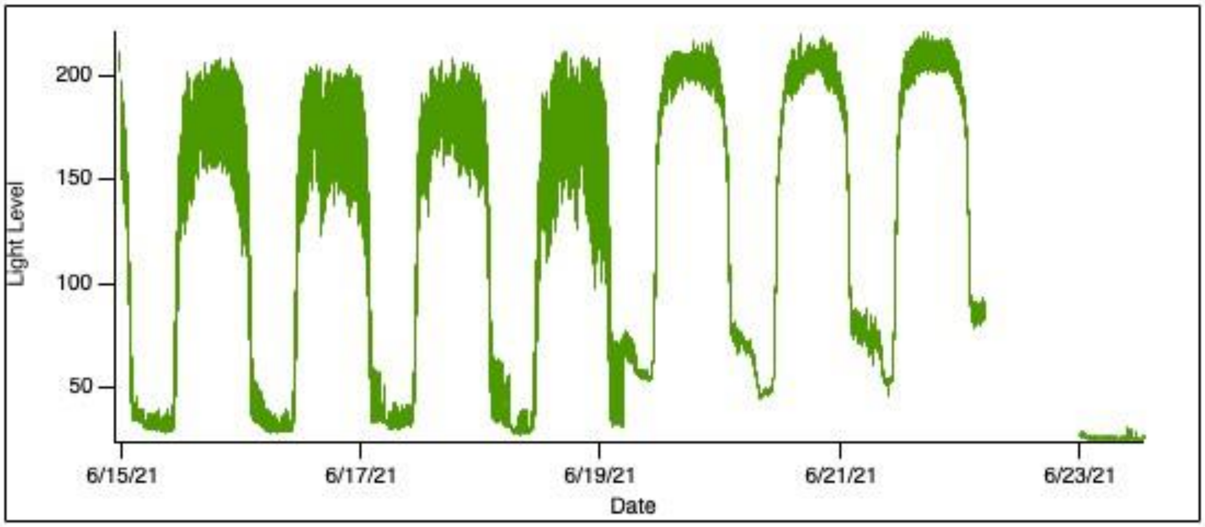

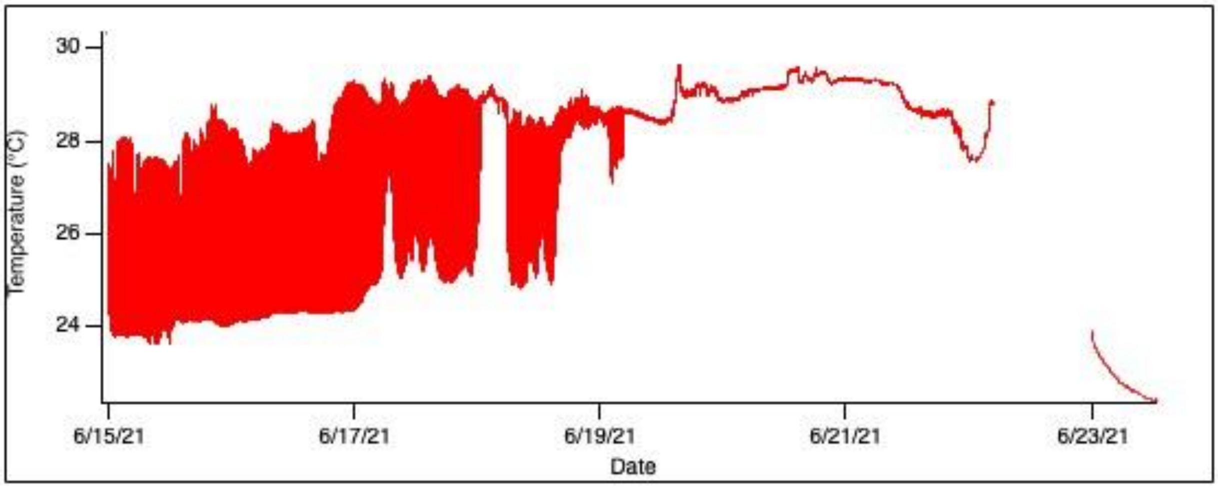

C_lim03: PSATLIFE tag recovered with 28 days of archived data reveal consistent changes in depth, temperature, and light level, indicating **survival.**

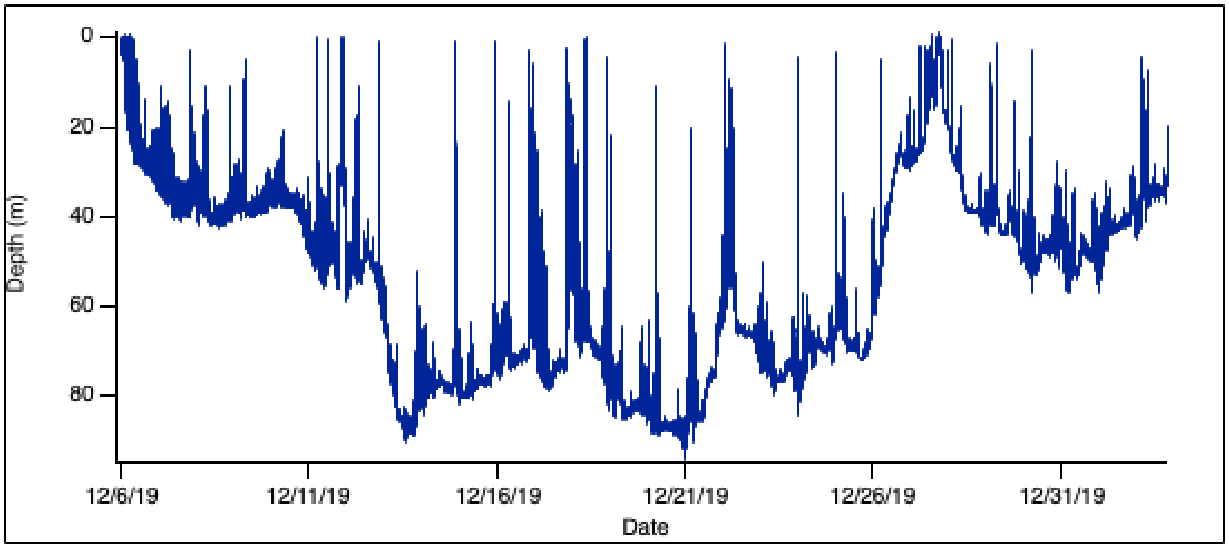

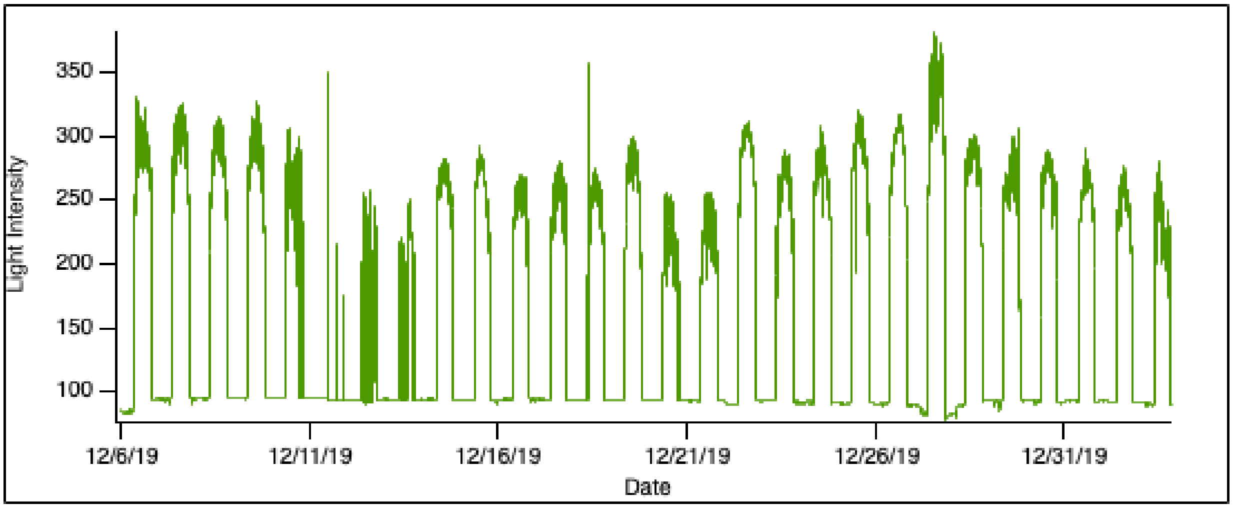

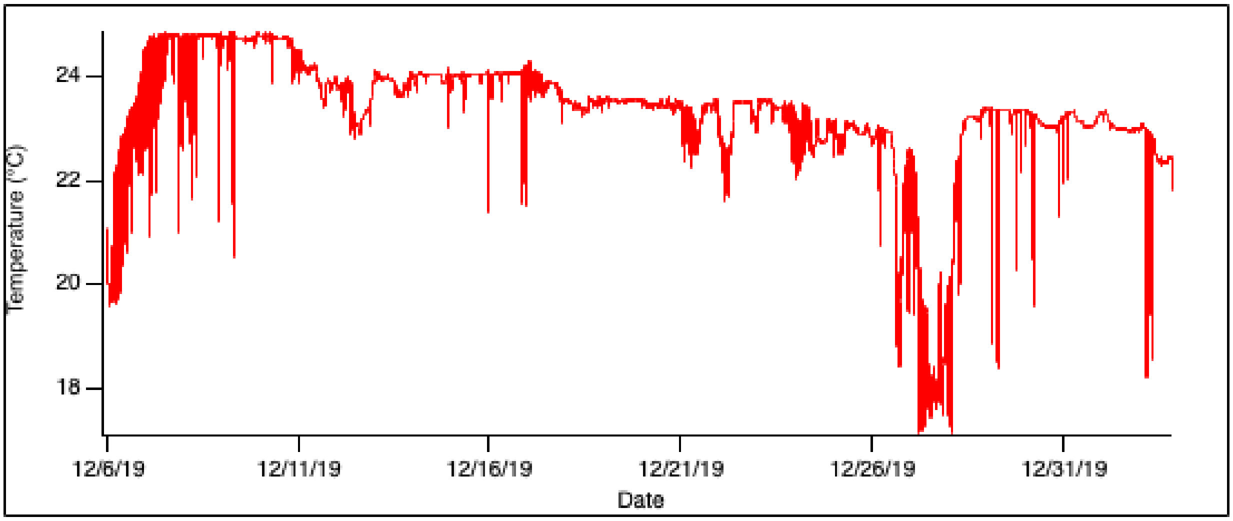

G_cuv04: sPAT tag with 51 days of transmitted data from daily summaries reveal normal variations in light, depth, and temperature, indicating **survival**. Tag released 9 days early..

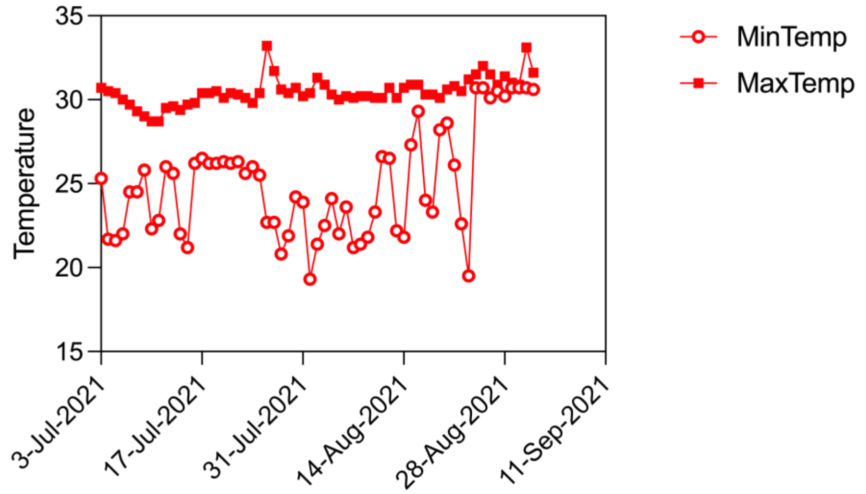

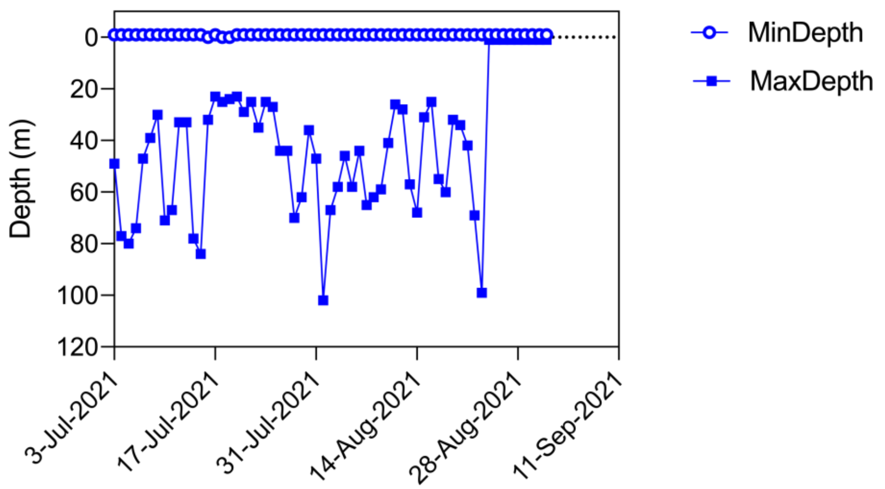

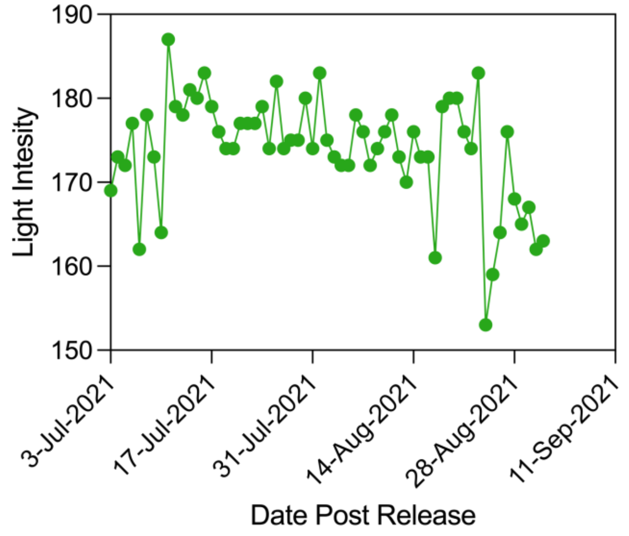

C_leu06: PSATLIFE tag recovered with 28 days of archived data reveal consistent changes in depth, temperature, and light level, indicating **survival.**

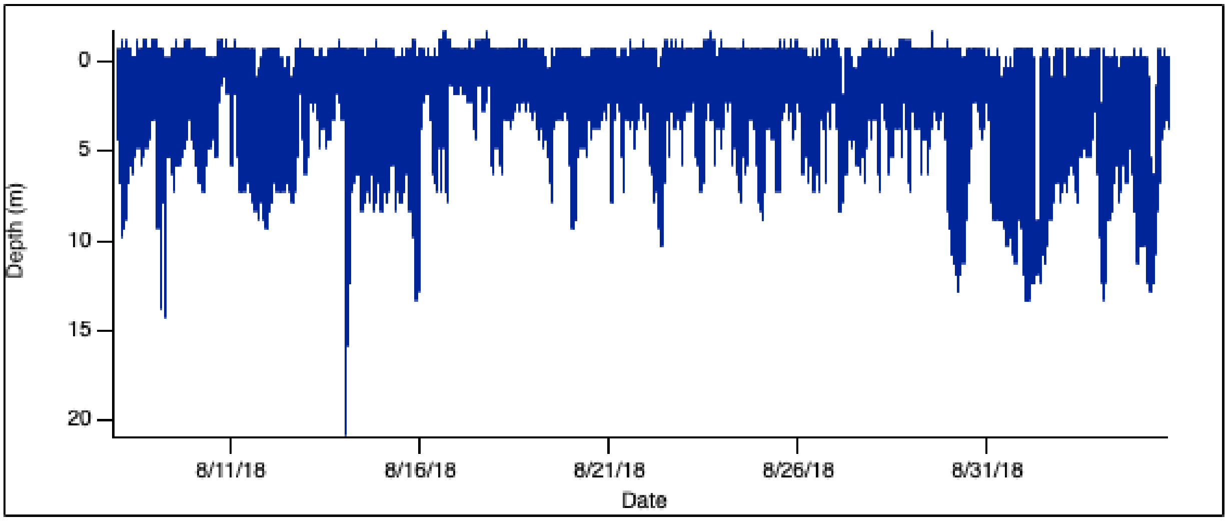

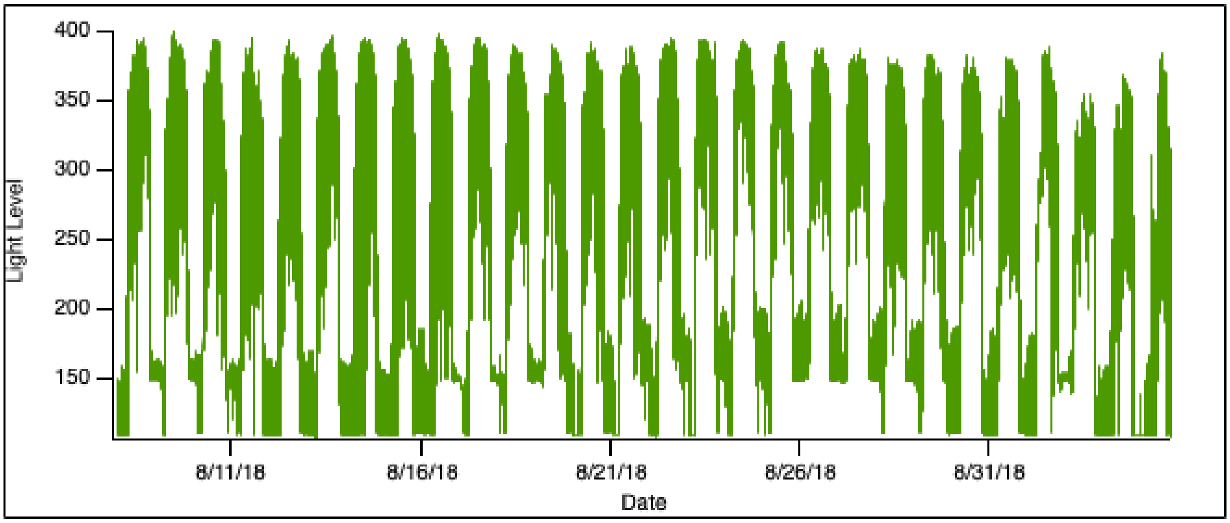

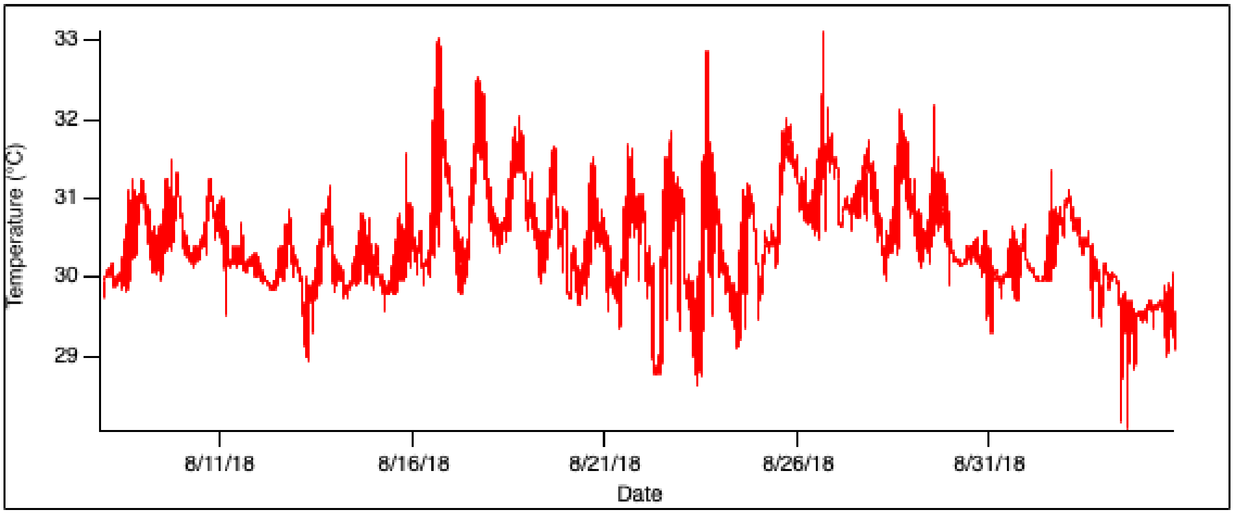

C_leu08: PSATLIFE tag with 28 days of trasnmitted data reveal consistent changes in depth and temperature, indicating **survival.** No data for light level and gaps in the data recovered indicate that data transmission was limited.

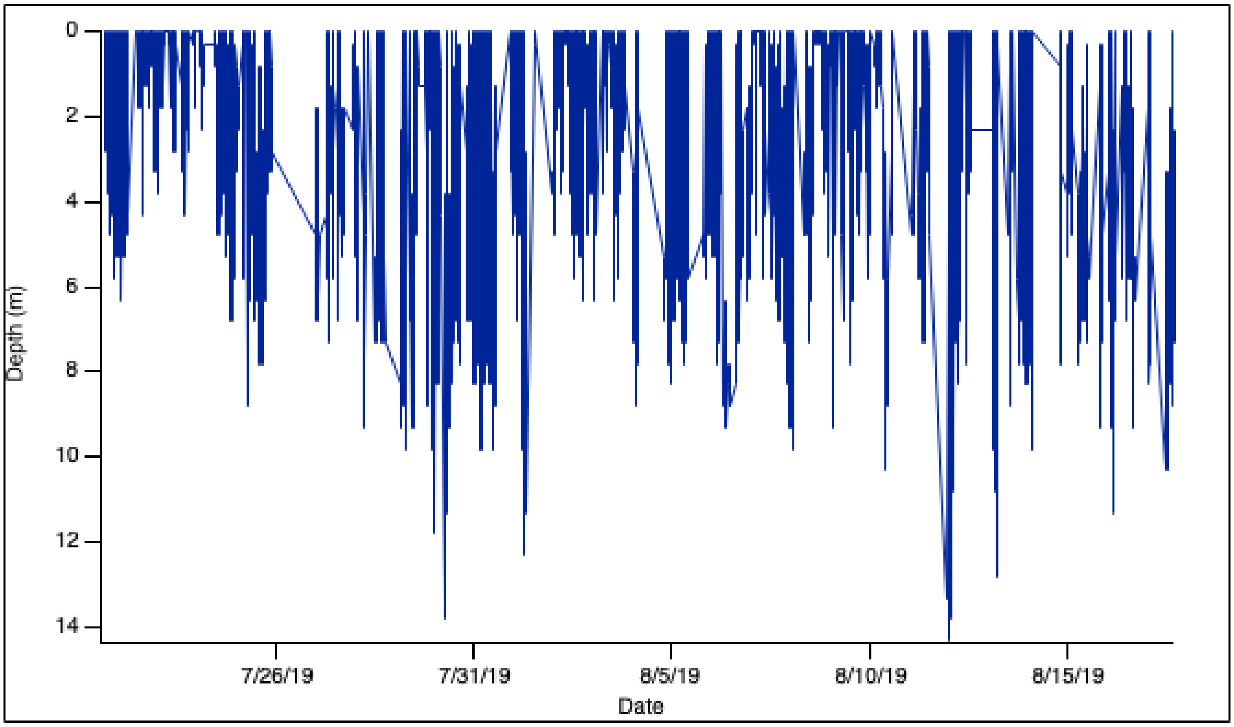

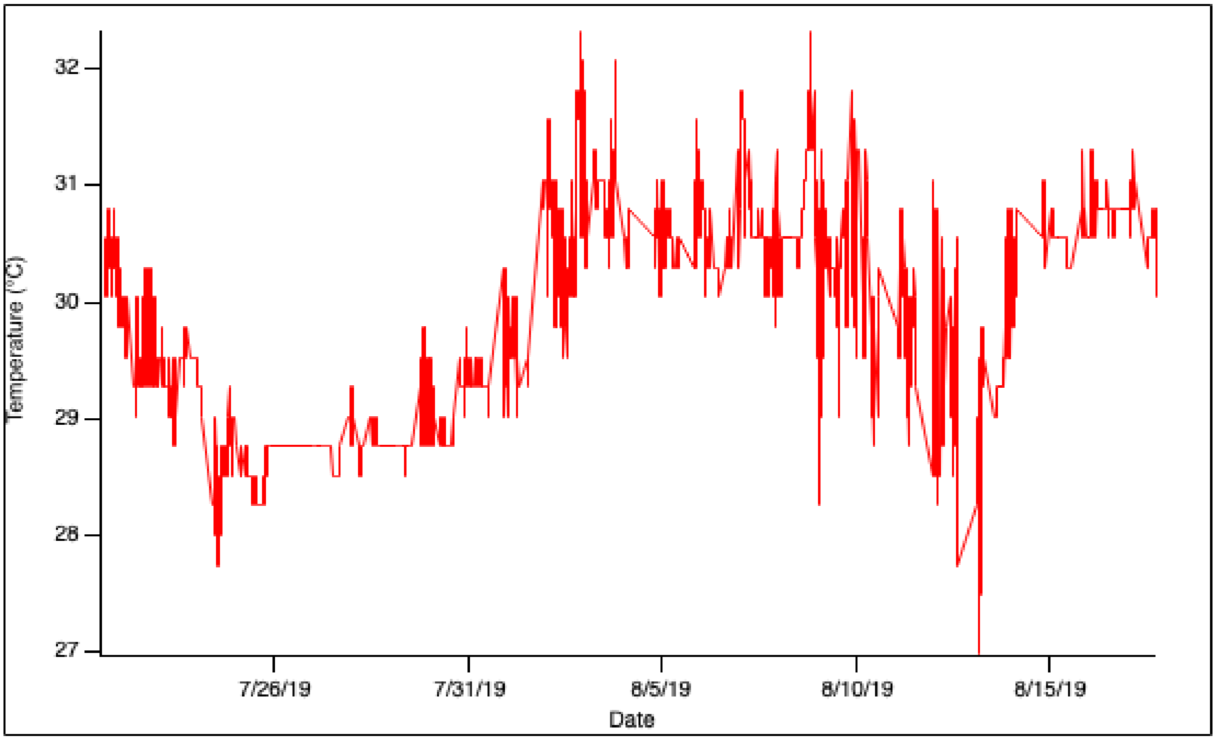

C_leu09: Mini-PAT tag with 28 days of transmitted data reveal consistent changes in depth and temperature, indicating **survival,** though data transmitted by satellite was limited.

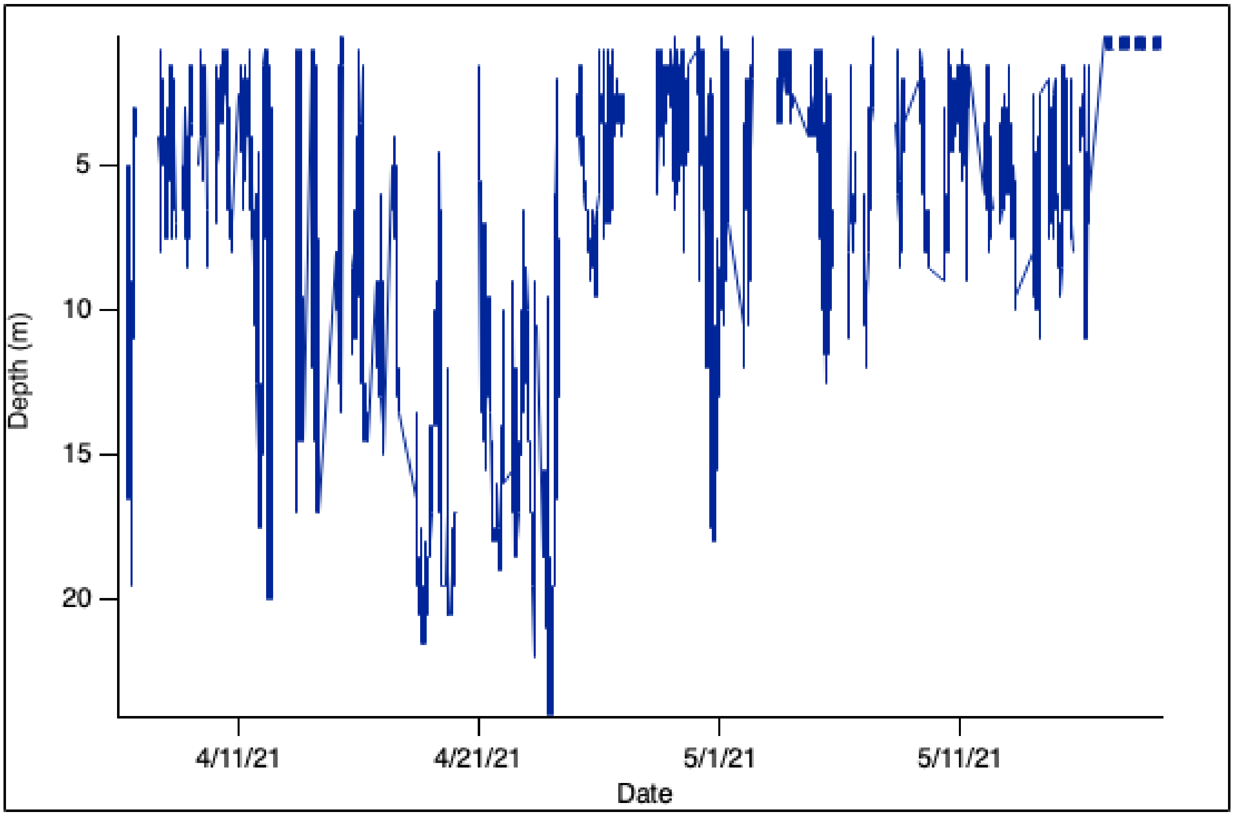

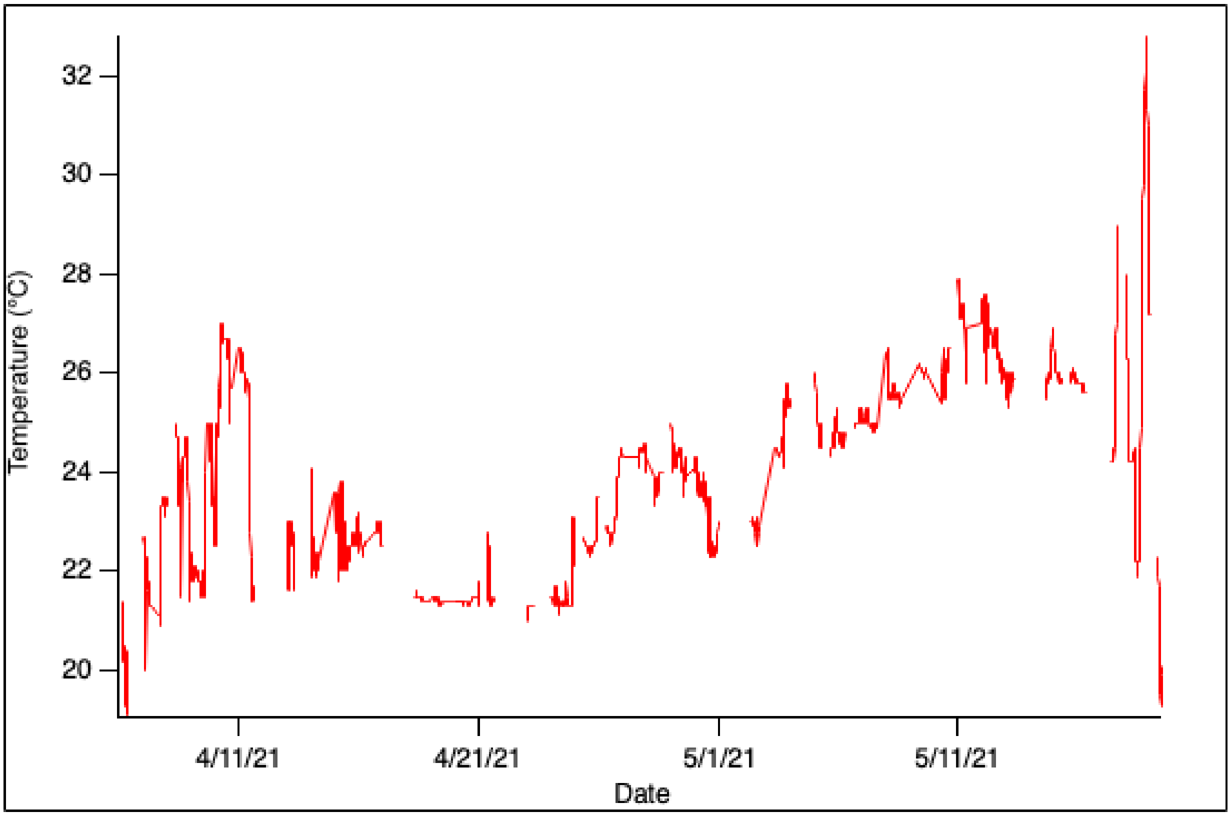

ADL

Time series from confirmed mortalities

C_lim07: After tagging, shark decreased depth gradually before remaining motionless at the bottom indicating **mortality.**

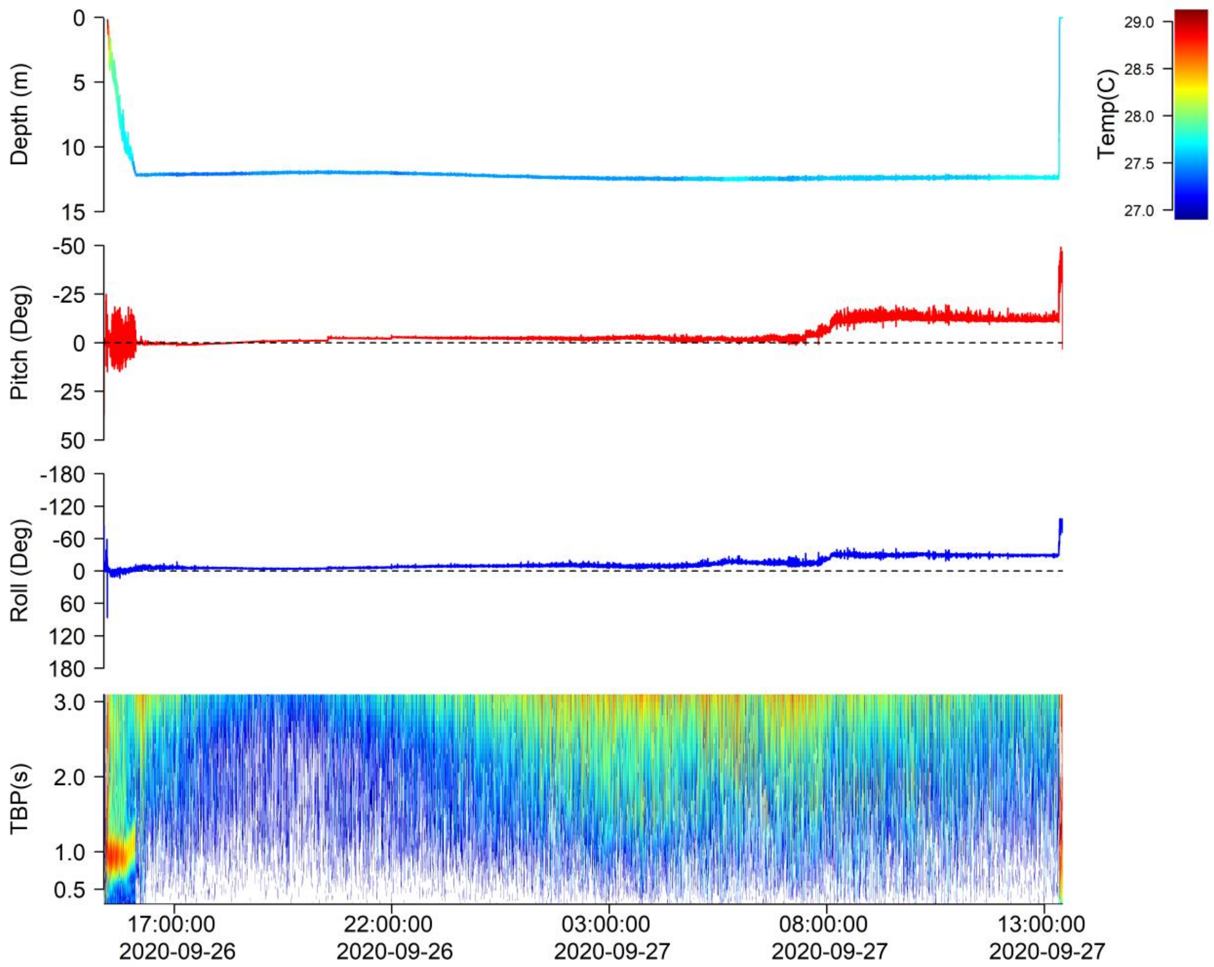

C_lim08: After tagging, shark swam up and down for approximately 5 hours, before remaining motionless on the bottom, indicating **mortality**

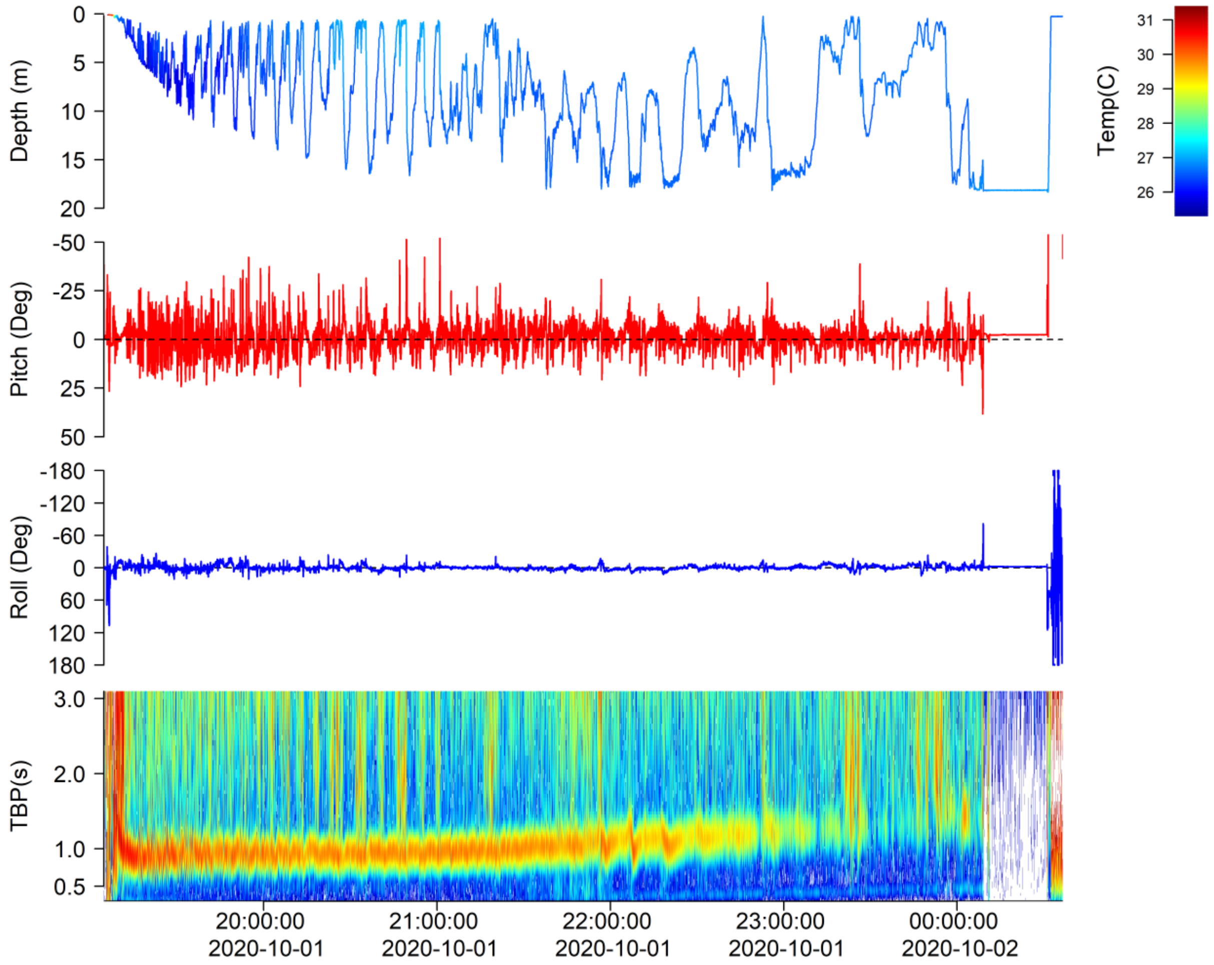

C_lim13: The shark was brought up on the beach and thrashed repeatedly during tagging. The tag was dislodged multiple times during the tagging process resulting in a handling time of 8 minutes. The shark was initially released at 20:38 central but had trouble clearing the first sand bar. The angler grabbed the shark again at 20:40 central, moved it past the first sand bar, and spent a couple of minutes reviving the shark. Shark was released a second time at 20:42 central. At approximately 21:10 central, the shark was seen **dead** in the surf.

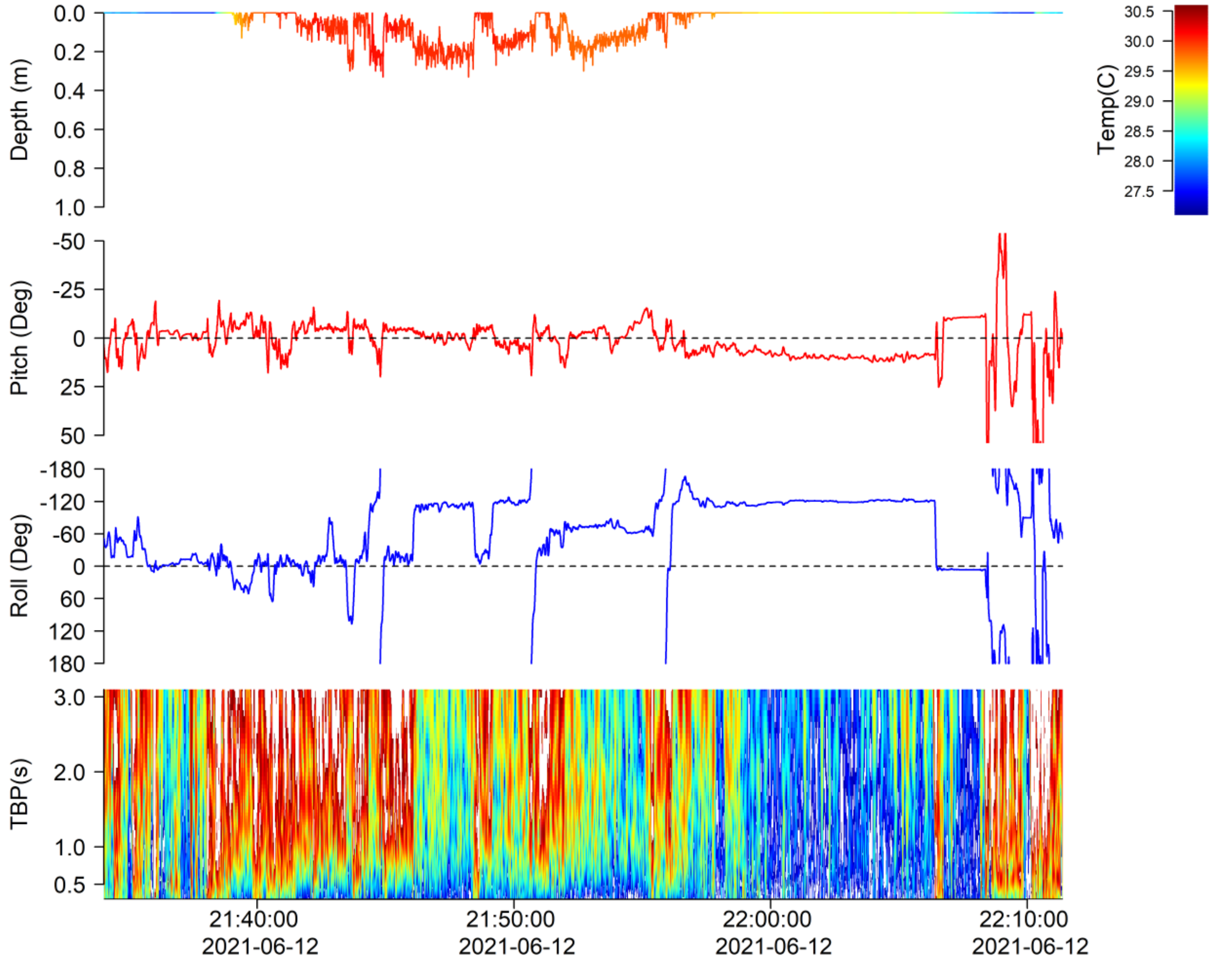

C_lim14: 1 hour after tagging, the shark went to bottom in less than where it remained for 2 hours at constant depth, indicating **mortality.** The tag was then ingested, indicated by low temperature variation for 2 days while depth continued to change. The tag was regurgitated, indicated by large depth decrease up to the surface, where it floated on the surface for 45 minutes before turning off.

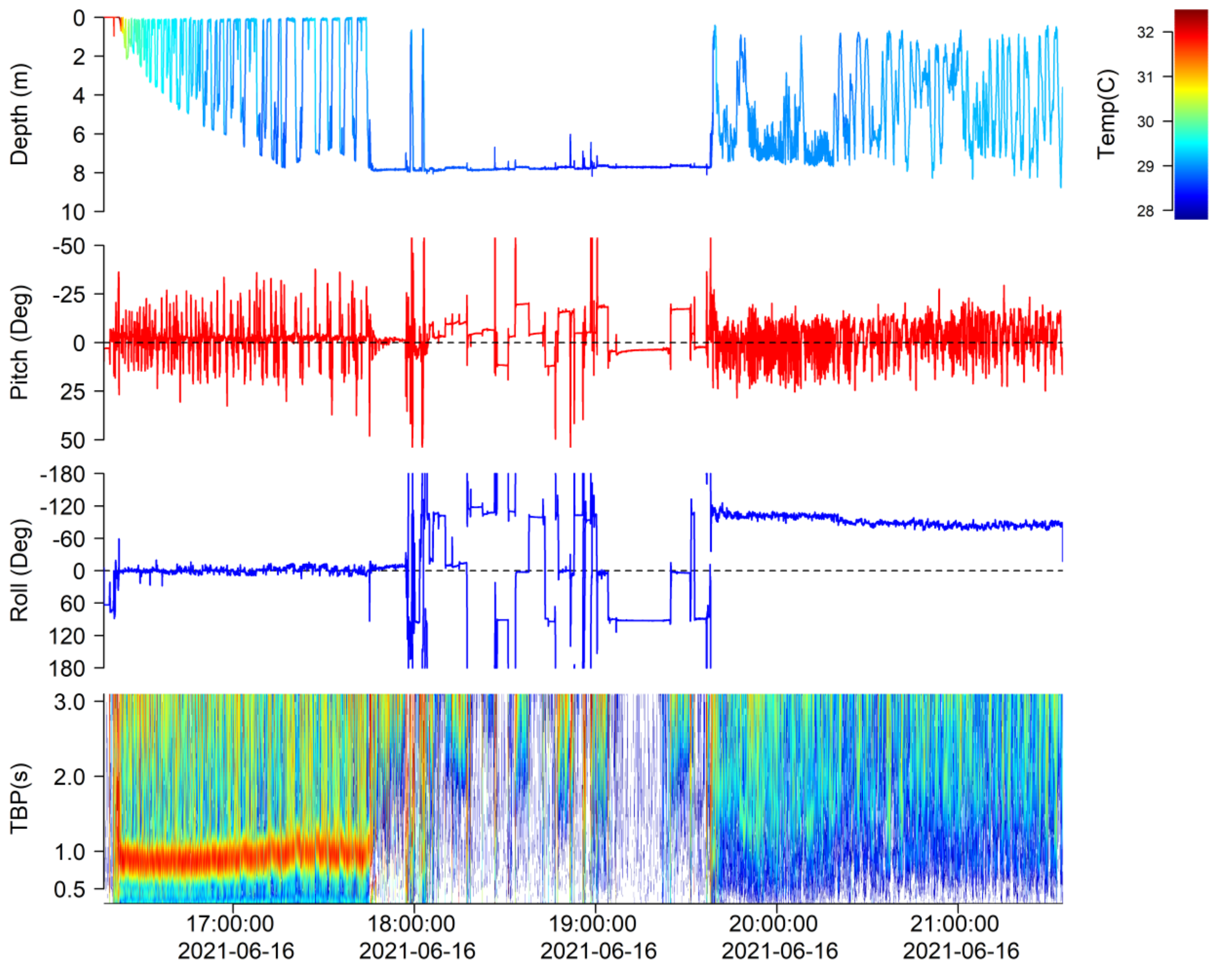

S_mok01: After tagging, shark moved at the surface for approximately 45 minutes, before remaining motionless on the bottom. indicating **mortality**

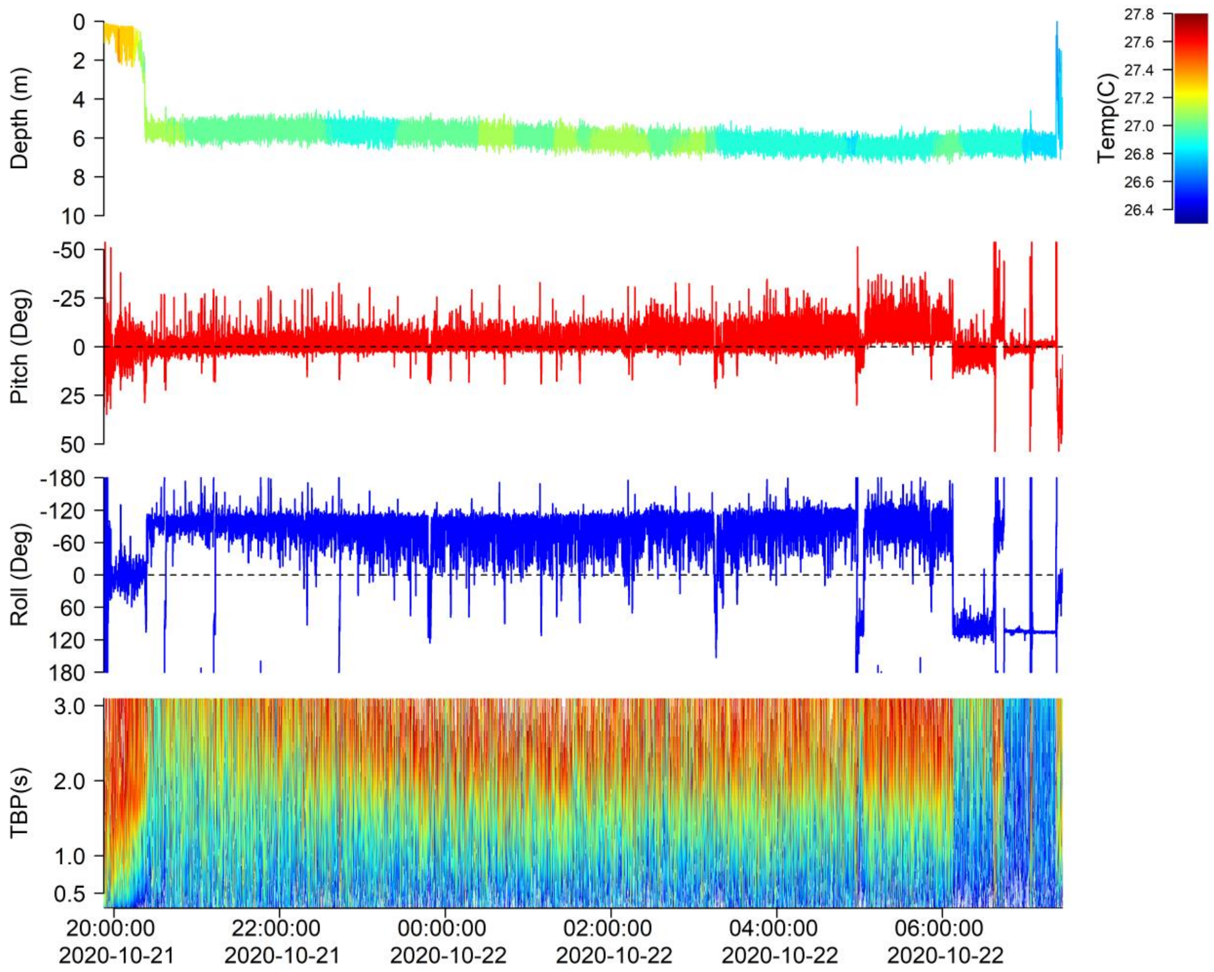

Time series from confirmed survivors

C_lim06: SURVIVE

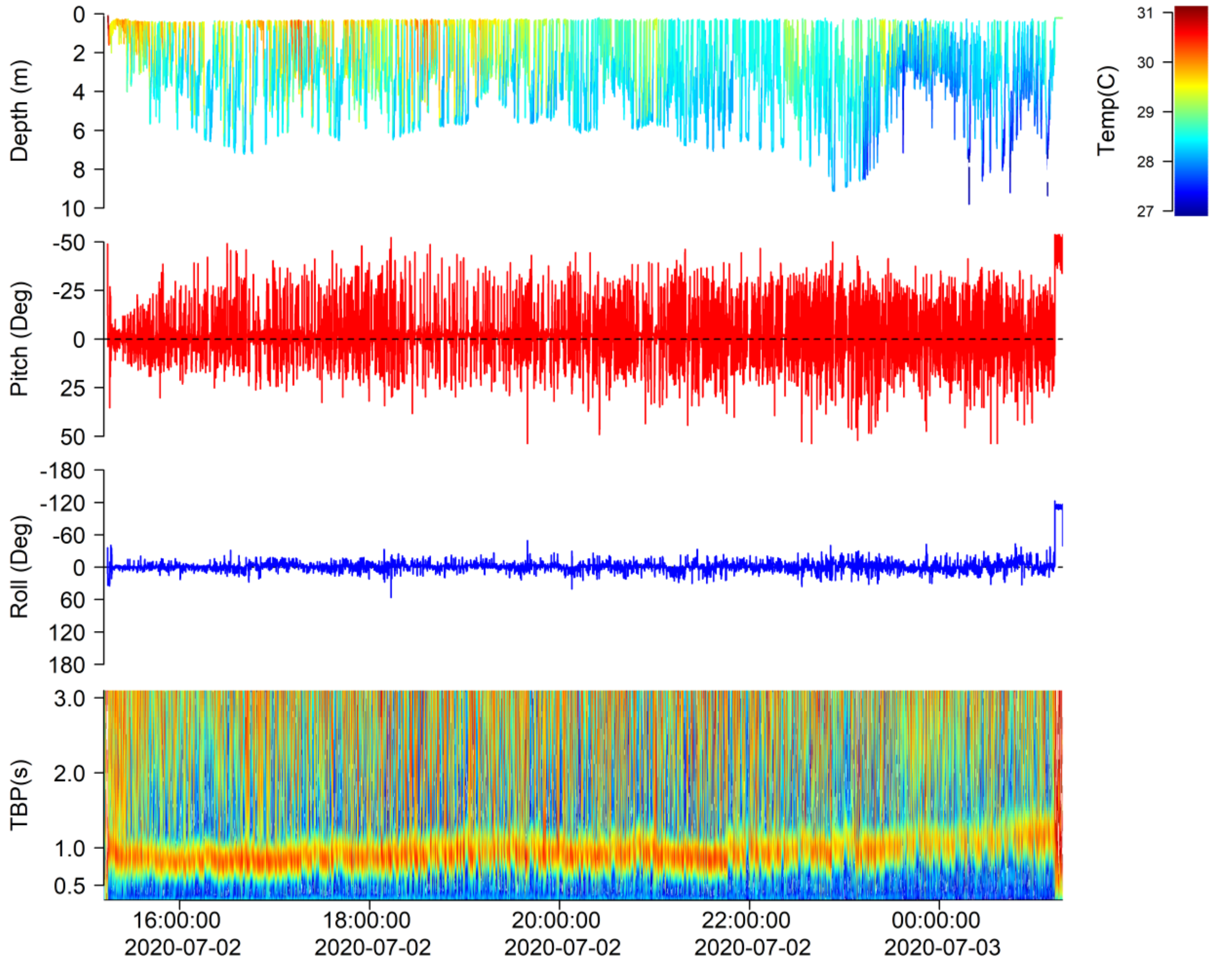

C_lim09: **SURVIVE**

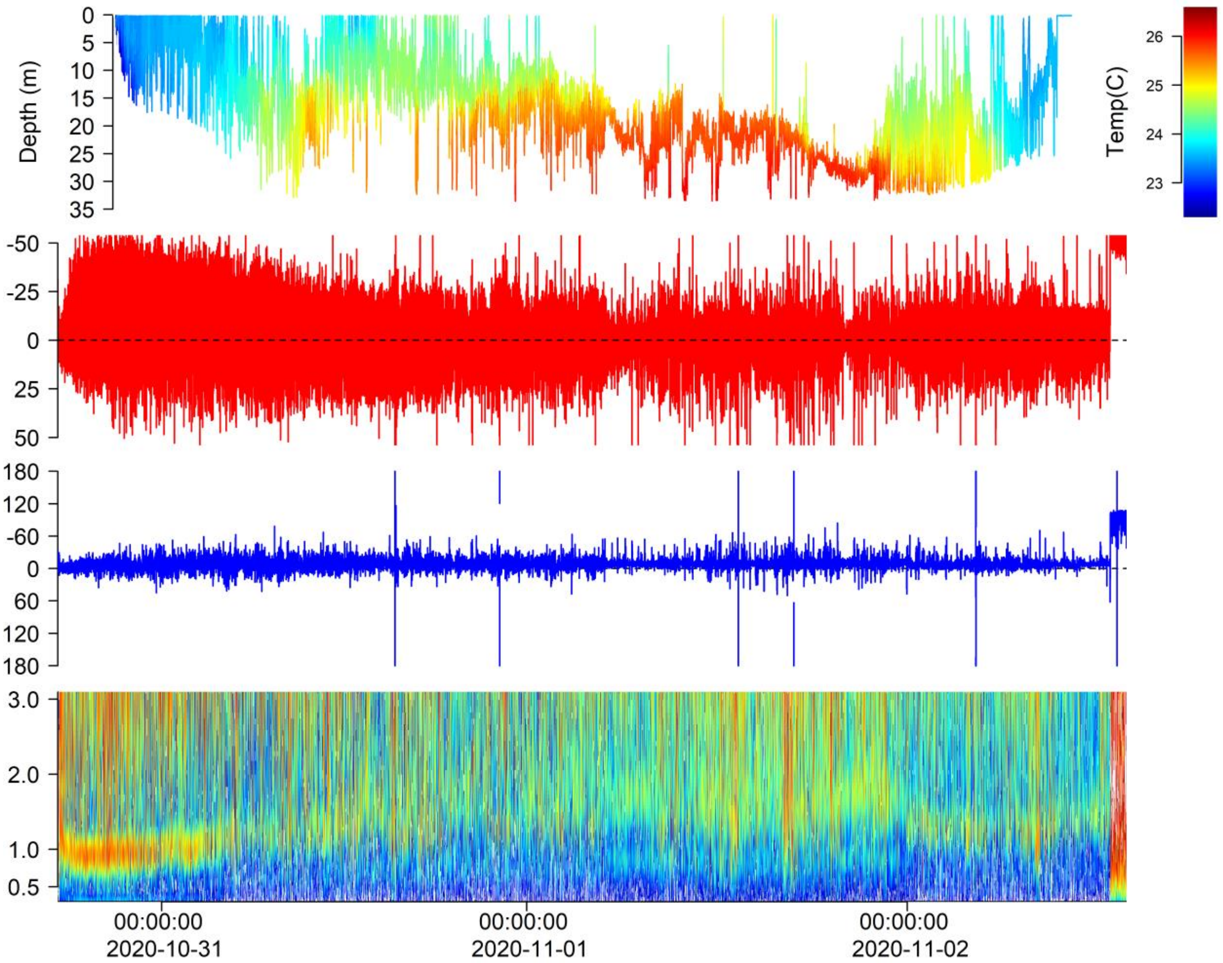

C_lim10: SURVIVE

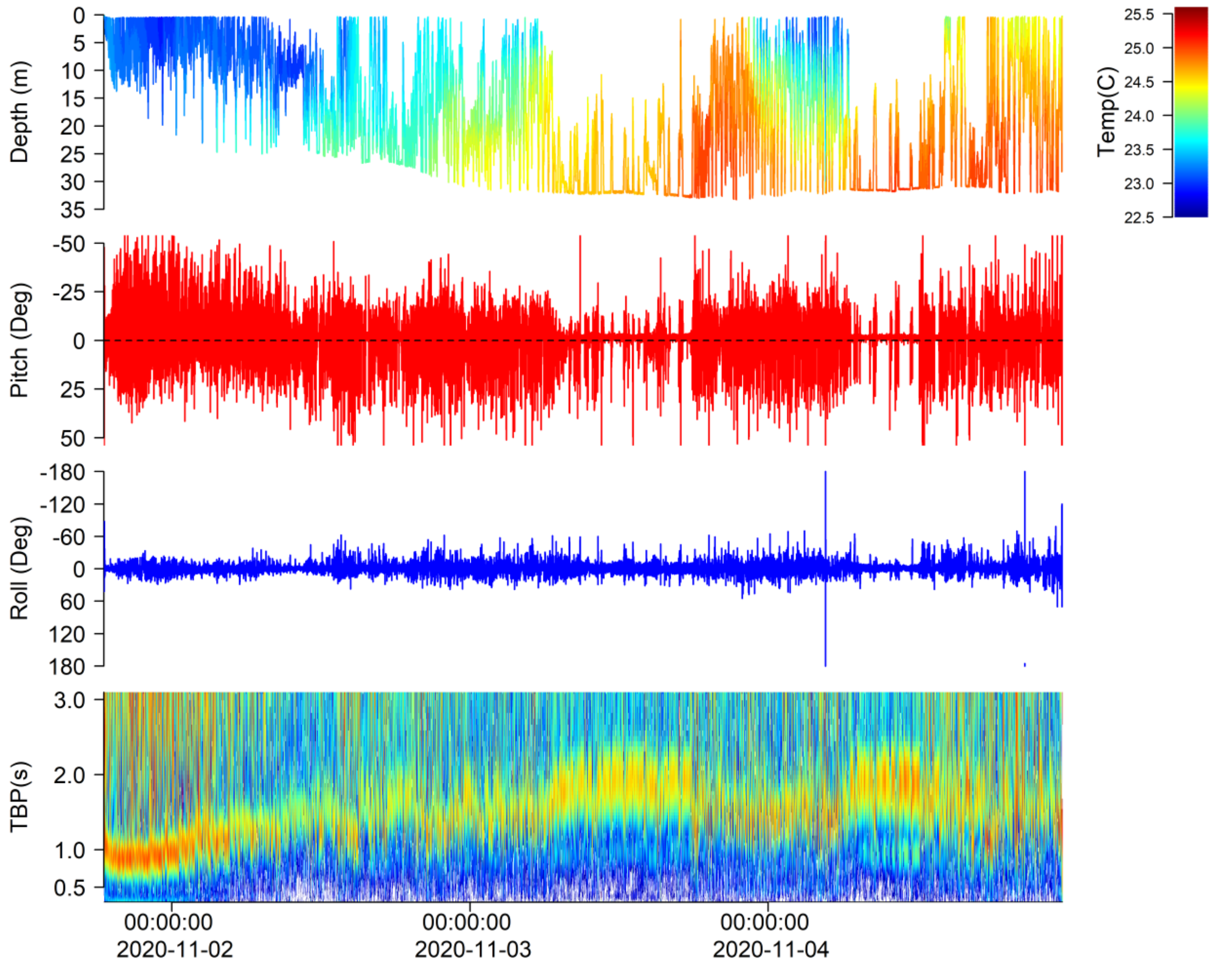

C_lim15: SURVIVE

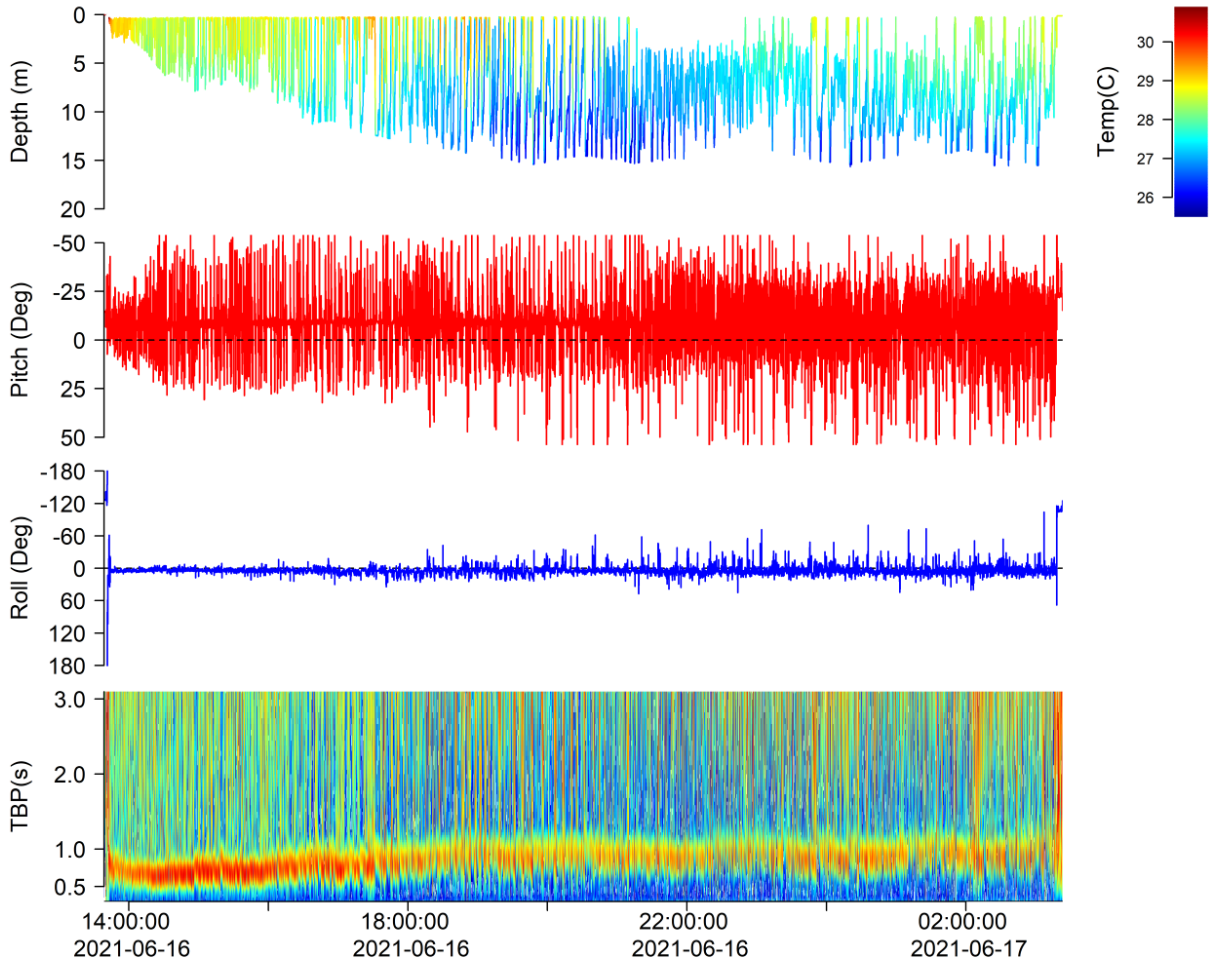

C_leu11: **SURVIVE**

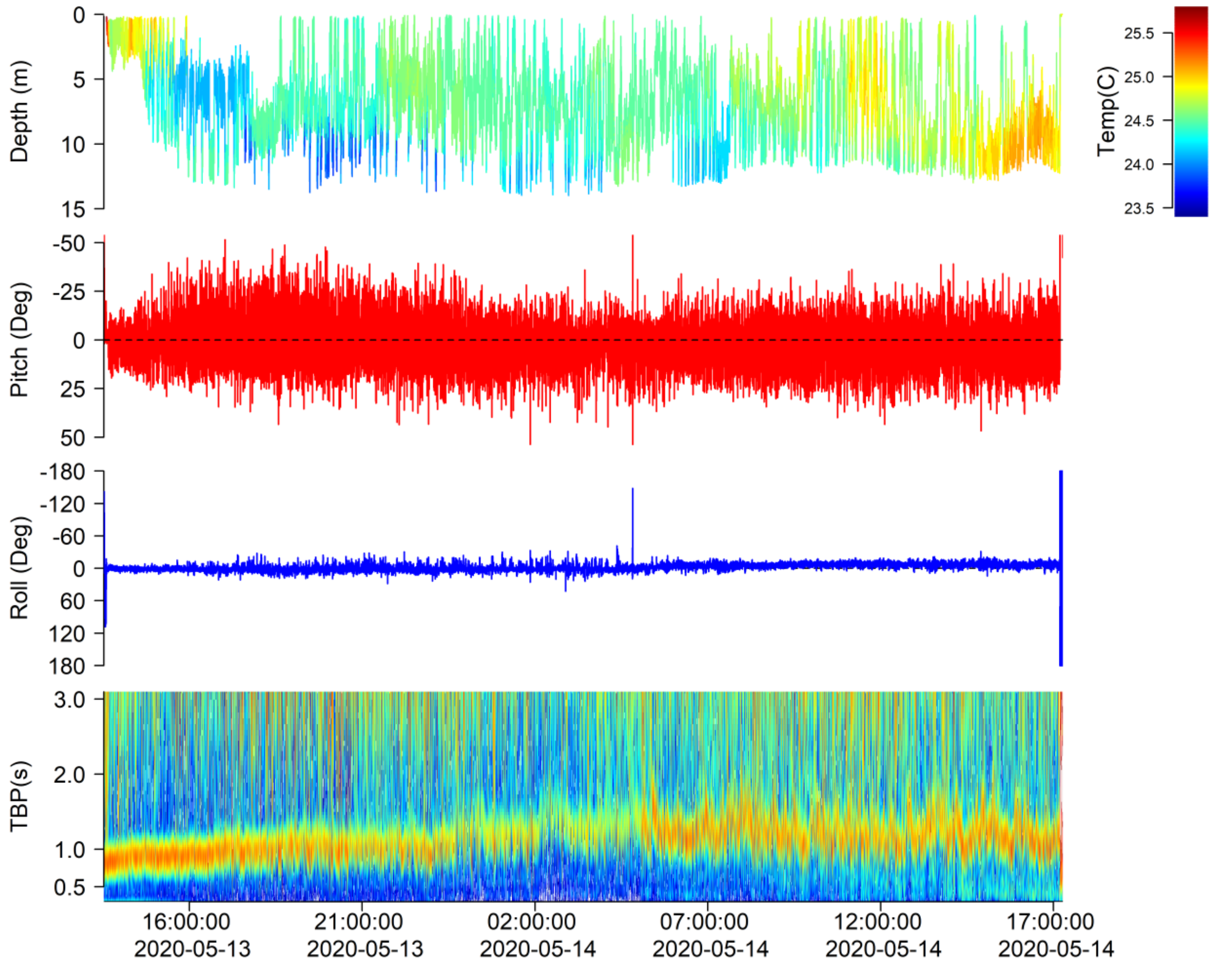

C_leu12: SURVIVE

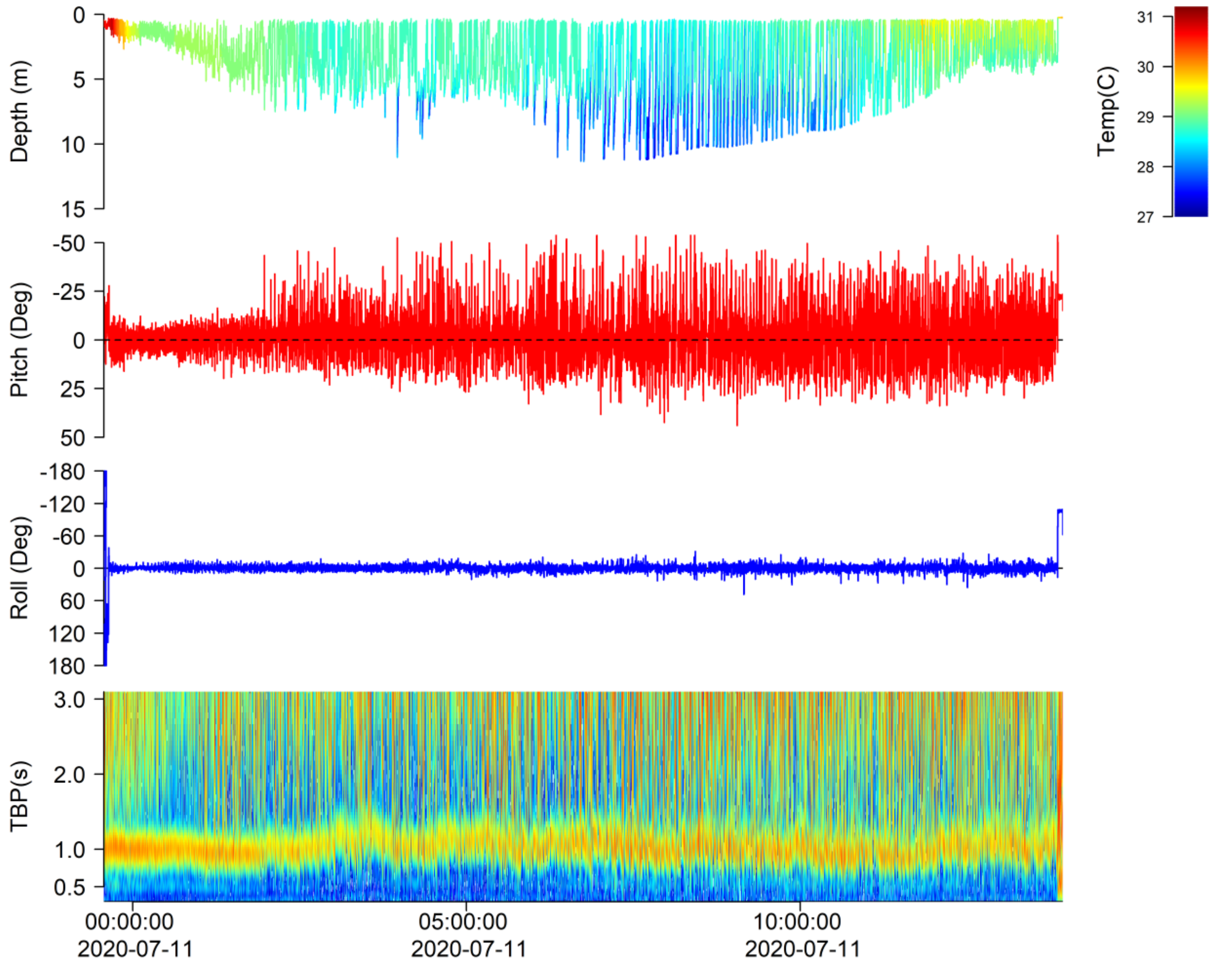

C_leu13: SURVIVE

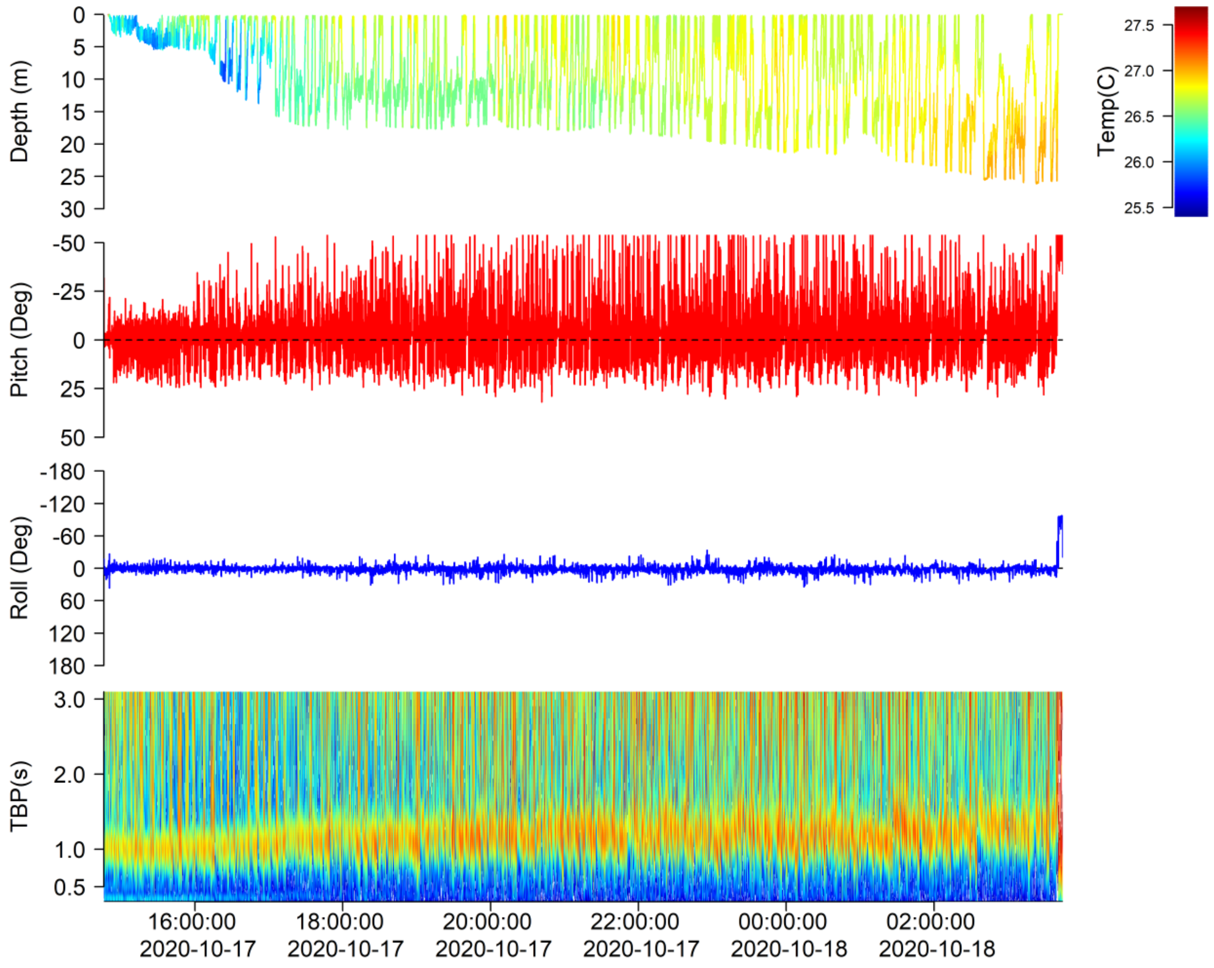

C_leu14: SURVIVE

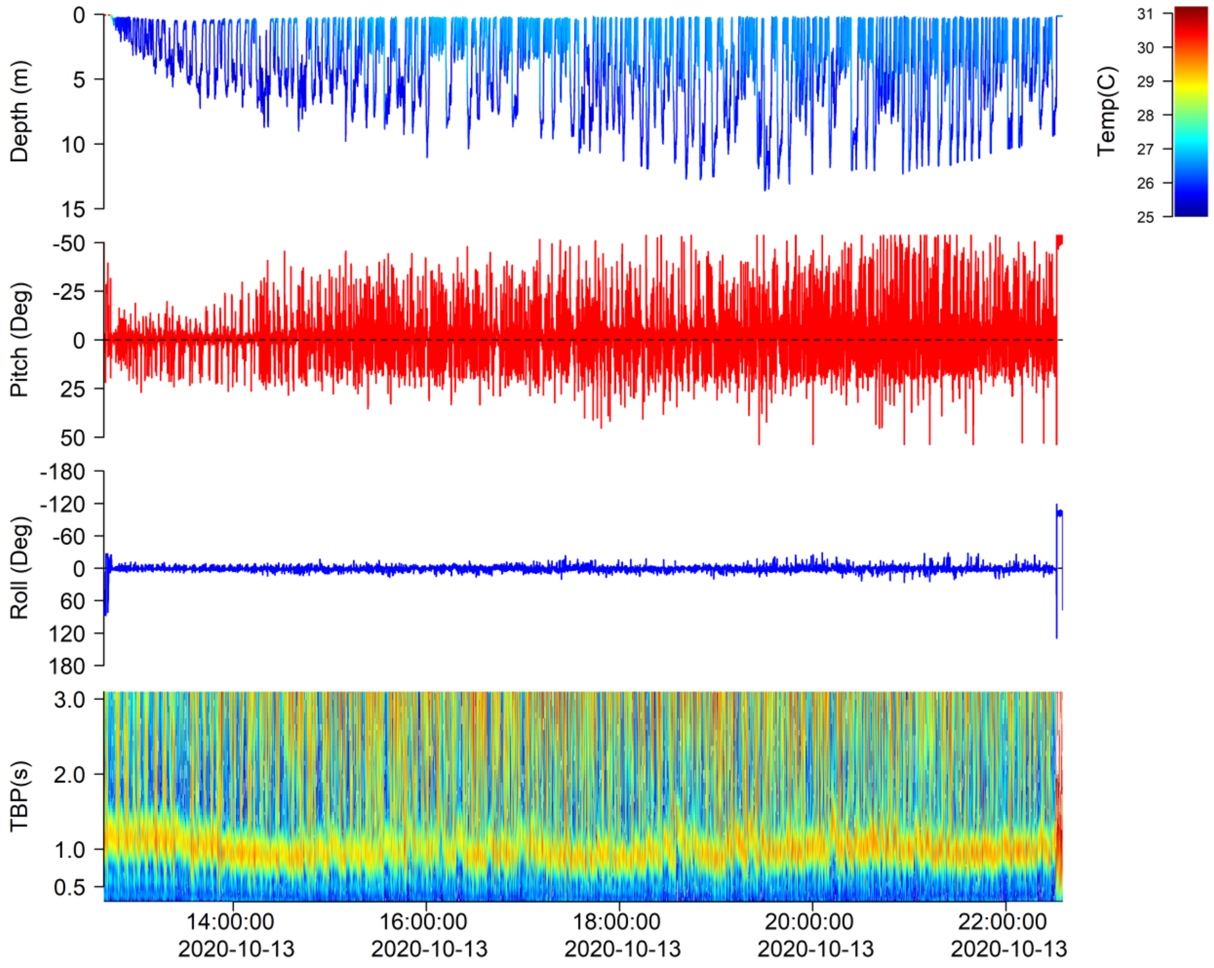

C_leu15: SURVIVE

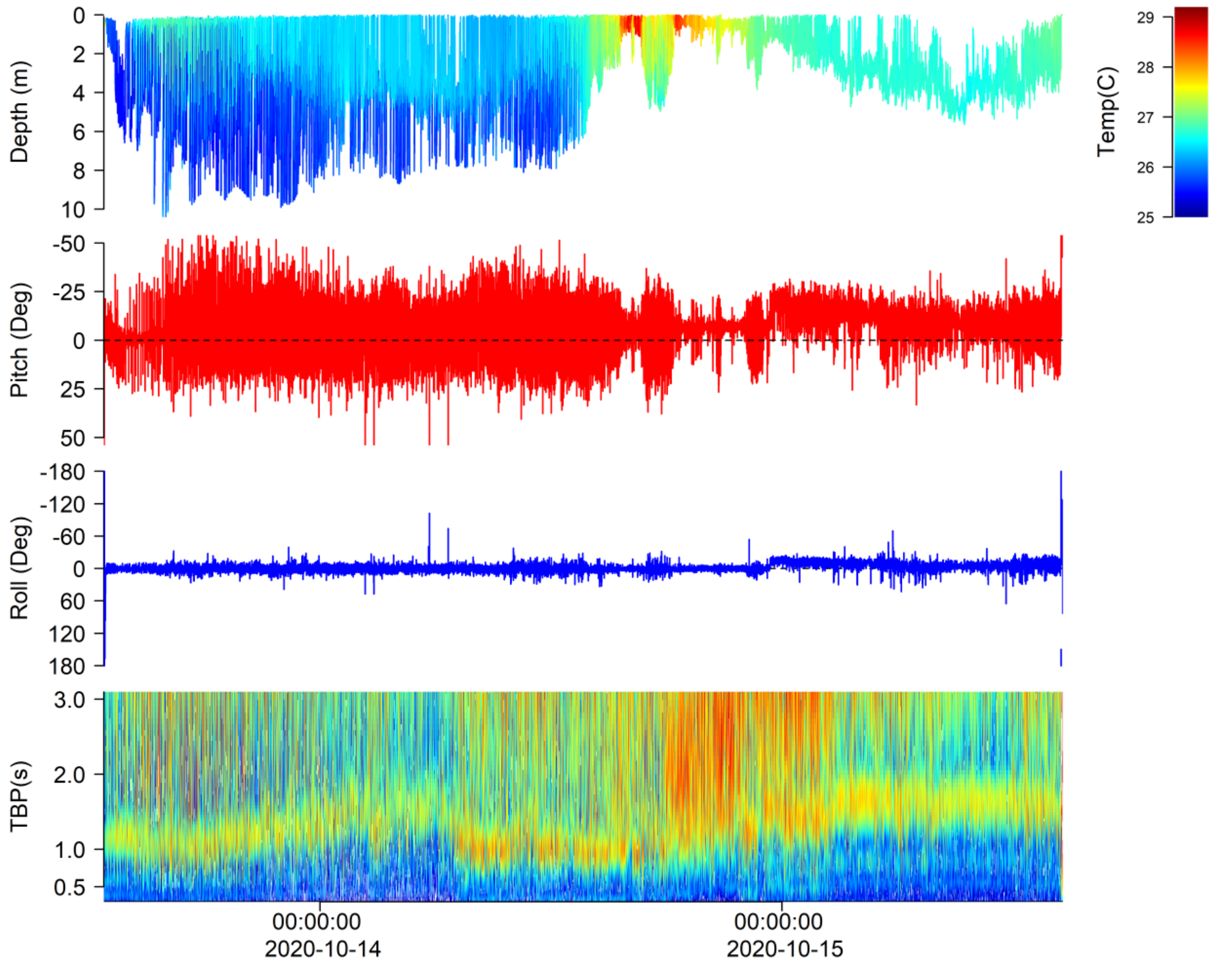

C_leu17: SURVIVE

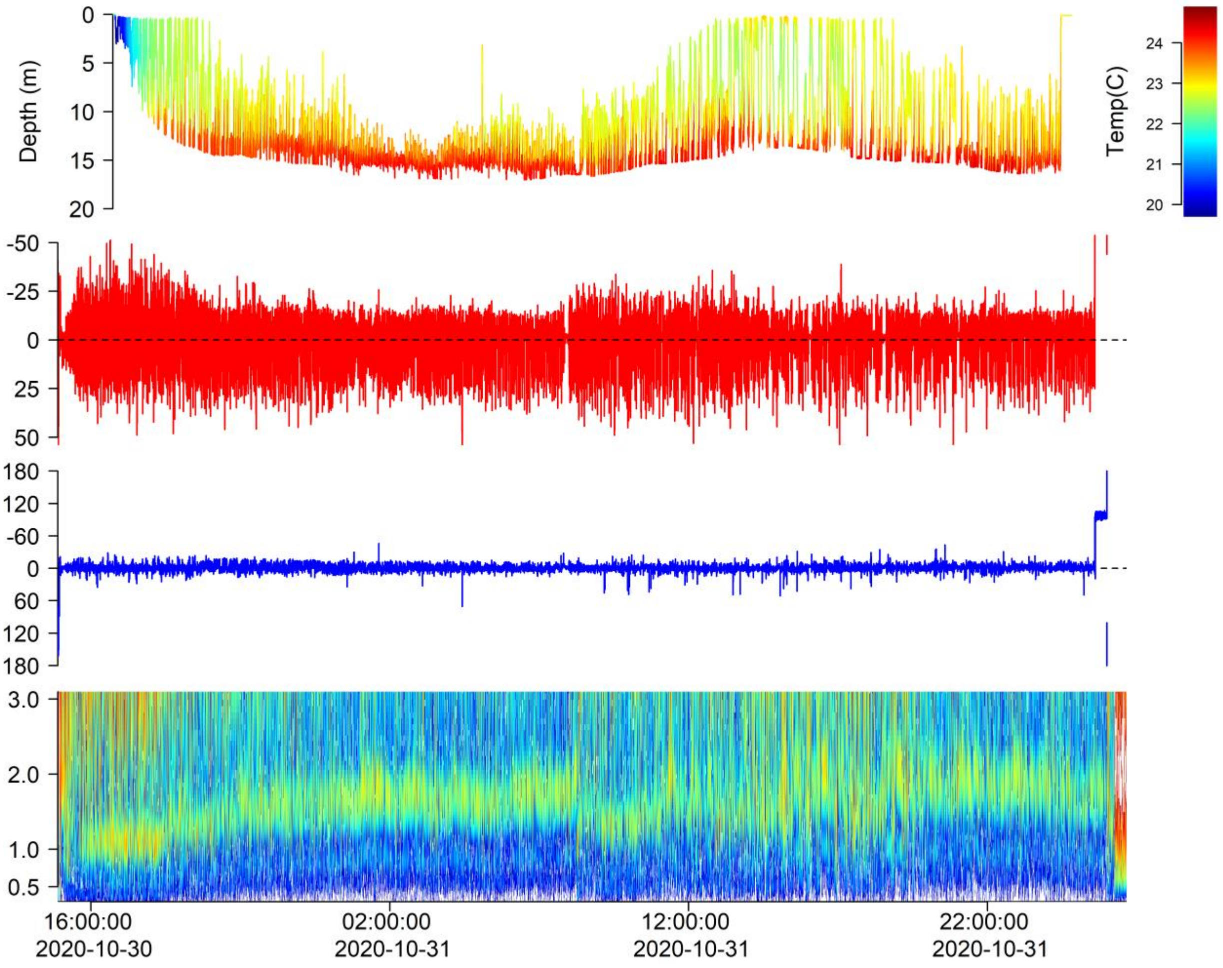

C_leu18: **SURVIVE**

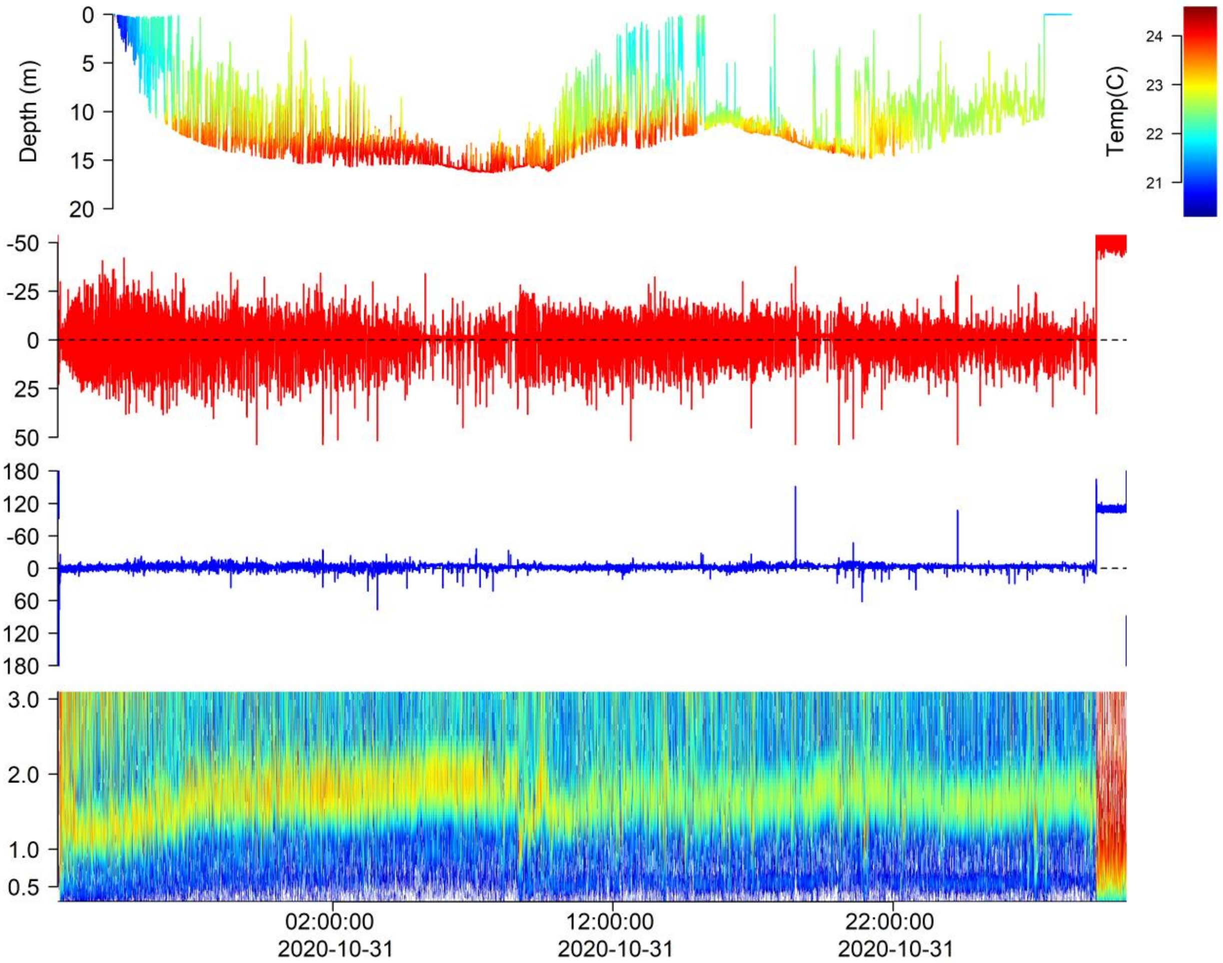

C_leu19: **SURVIVE**

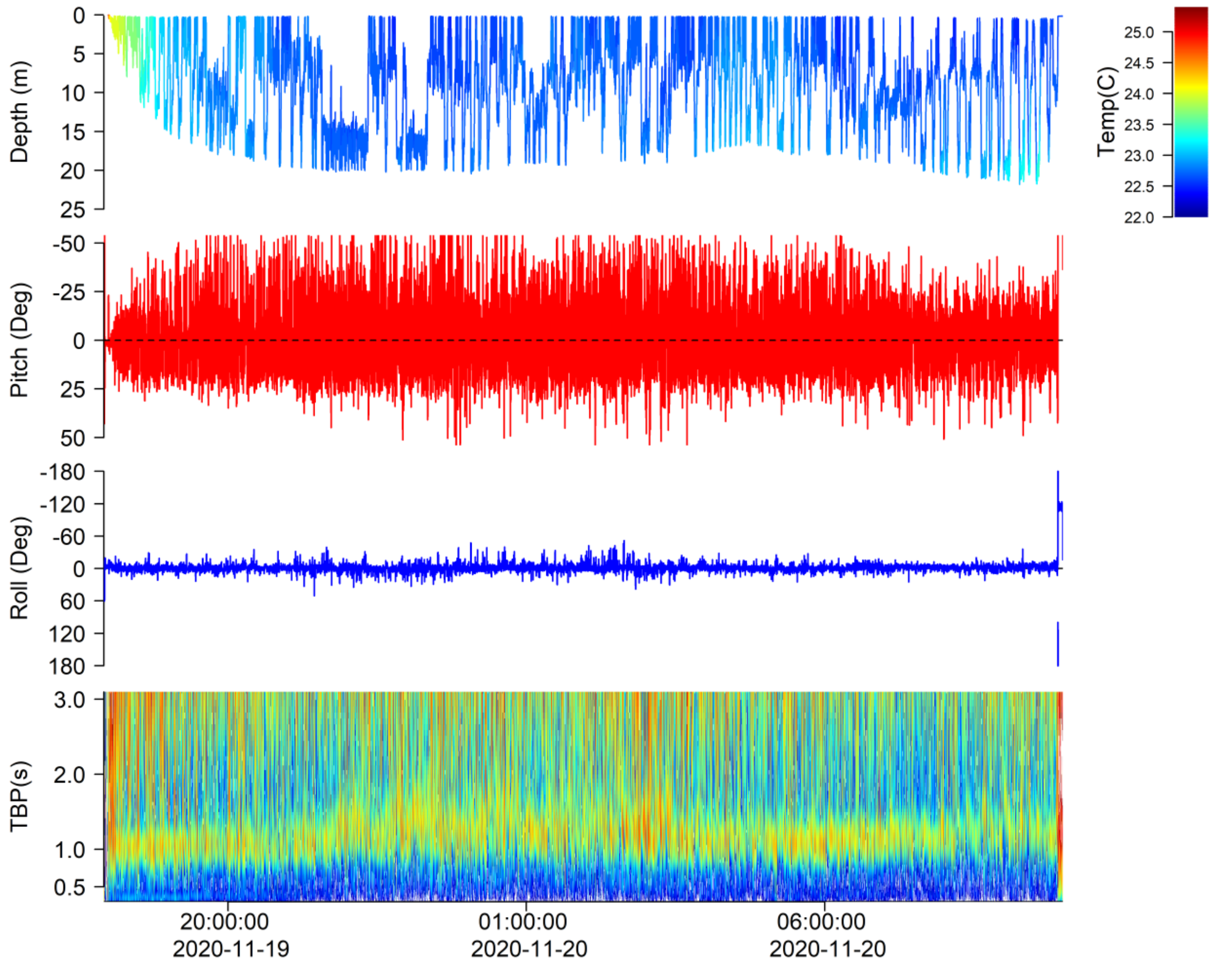

G_cuv06: SURVIVE

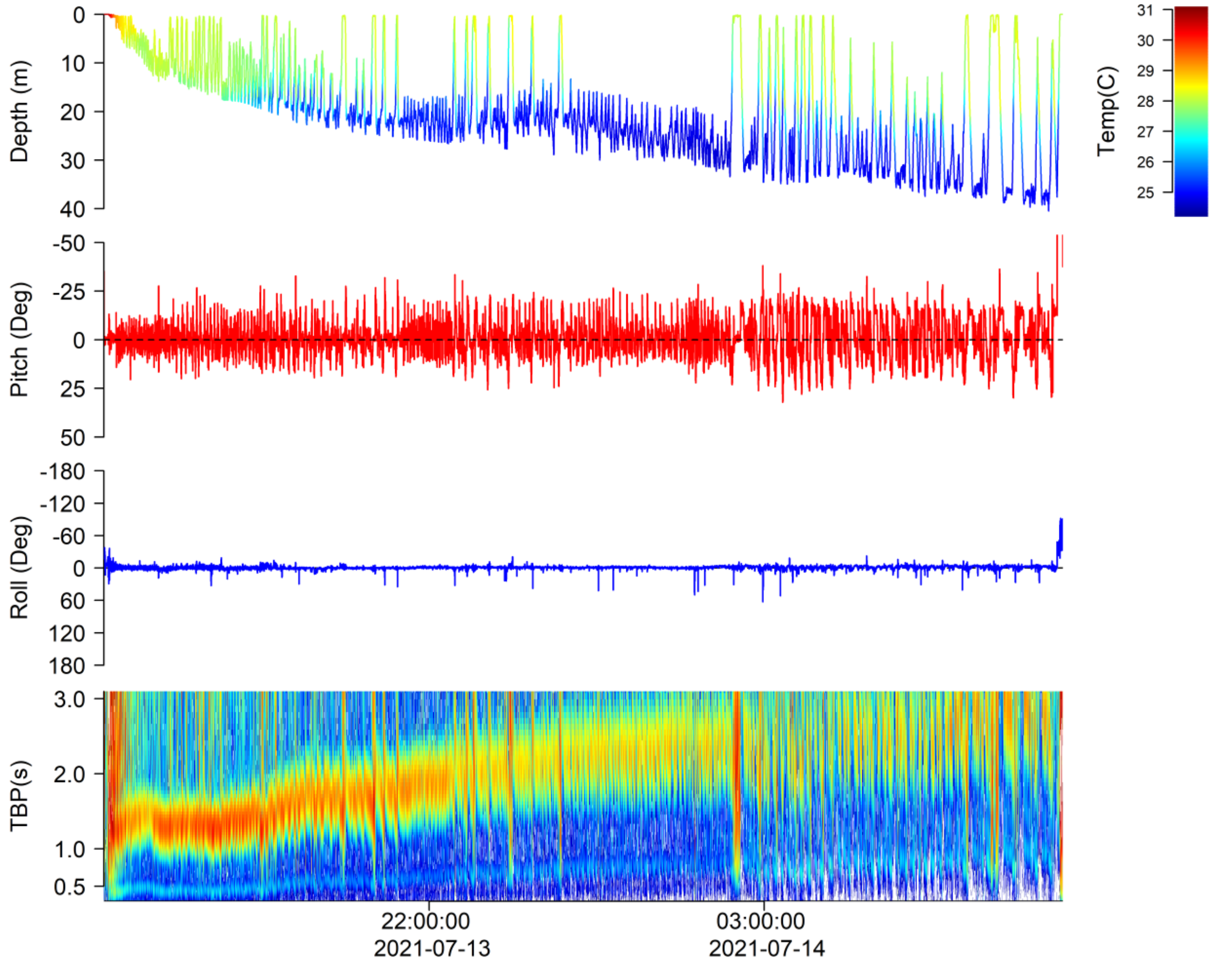

Supplement: S2 File — Includes all time series for all datasets obtained from tagged sharks. (PDF) [file pone.0281441.s002.pdf]
